# Proteogenomic Landscape of Breast Ductal Carcinoma Reveals Tumor Progression Characteristics and Therapeutic Targets

**DOI:** 10.1002/advs.202401041

**Published:** 2024-10-17

**Authors:** Ganfei Xu, Juan Yu, Jiacheng Lyu, Mengna Zhan, Jie Xu, Minjing Huang, Rui Zhao, Yan Li, Jiajun Zhu, Jinwen Feng, Subei Tan, Peng Ran, Zhenghua Su, Xinhua Liu, Jianyuan Zhao, Hongwei Zhang, Chen Xu, Jun Chang, Yingyong Hou, Chen Ding

**Affiliations:** ^1^ State Key Laboratory of Genetic Engineering School of Life Sciences Human Phenome Institute Department of Pathology Zhongshan Hospital, Fudan University Shanghai 200433 China; ^2^ Institute for Developmental and Regenerative Cardiovascular Medicine MOE‐Shanghai Key Laboratory of Children's Environmental Health Xinhua Hospital Shanghai Jiao Tong University School of Medicine Shanghai 200092 China; ^3^ Departments of Cancer Research Institute Affiliated Cancer Hospital of Xinjiang Medical University Xinjiang Key Laboratory of Translational Biomedical Engineering Urumqi 830000 P. R. China

**Keywords:** AKR1C1, breast ductal carcinoma, progression, proteogenomics, sterol hormone receptor

## Abstract

Multi‐omics studies of breast ductal carcinoma (BRDC) have advanced the understanding of the disease's biology and accelerated targeted therapies. However, the temporal order of a series of biological events in the progression of BRDC is still poorly understood. A comprehensive proteogenomic analysis of 224 samples from 168 patients with malignant and benign breast diseases is carried out. Proteogenomic analysis reveals the characteristics of linear multi‐step progression of BRDC, such as tumor protein P53 (*TP53*) mutation‐associated estrogen receptor 1 (ESR1) overexpression is involved in the transition from ductal hyperplasia (DH) to ductal carcinoma in situ (DCIS). 6q21 amplification‐associated nuclear receptor subfamily 3 group C member 1 (NR3C1) overexpression helps DCIS_Pure (pure DCIS, no histologic evidence of invasion) cells avoid immune destruction. The T‐cell lymphoma invasion and metastasis 1, androgen receptor, and aldo‐keto reductase family 1 member C1 (TIAM1‐AR‐AKR1C1) axis promotes cell invasion and migration in DCIS_adjIDC (DCIS regions of invasive cancers). In addition, AKR1C1 is identified as a potential therapeutic target and demonstrated the inhibitory effect of aspirin and dydrogesterone as its inhibitors on tumor cells. The integrative multi‐omics analysis helps to understand the progression of BRDC and provides an opportunity to treat BRDC in different stages.

## Graphical abstract

A proteogenomic analysis of 224 breast ductal carcinoma (BRDC) samples revealed the characteristics of linear multi‐step progression. Aldo‐keto reductase family 1 member C1 (AKR1C1) was identified as a potential therapeutic target and demonstrated the inhibitory effect of aspirin and dydrogesterone as its inhibitors on tumor cells. This analysis helps to understand the progression of BRDC and provides treatment opportunities.

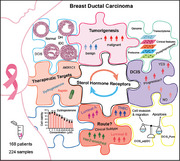

## 1 Introduction

Breast cancer (BC) is one of the most common cancers in women,^[^ ^1^ ^]^ with high heterogeneity in its morphology, molecular expression profile, and clinical course.^[^ ^2^, ^3^, ^4^ ^]^ Breast ductal carcinoma (BRDC) is the most common type of BC and has unique clinical and pathological features. Histopathologically, the classic progression model of human BRDC was a linear multi‐step process that initiates as ductal hyperplasia (DH), progresses to ductal carcinoma in situ (DCIS), and evolves into invasive ductal carcinoma (IDC).^[^ ^5^ ^]^ IDC was classified into four major clinical subtypes: luminal A, luminal B, HER2‐enriched, and TNBC based on histopathological criteria including the expression of hormone receptors (estrogen receptor and/or progesterone receptor) and/or human epidermal growth factor receptor 2 (HER2).^[^ ^6^ ^]^

In the past decade, many studies have characterized the multi‐omics landscape of certain stages of the progression of BRDC. Recent next‐generation sequencing‐based studies, including The Cancer Genome Atlas (TCGA) program, have uncovered the genetic landscape of invasive breast cancers,^[^ ^7^, ^8^, ^9^, ^10^ ^]^ revealing driver mutations in *TP53*, *PIK3CA*, *PTEN*, *BRCA2*, *ESR1*, *GATA3*, *KMT2C*, *NCOR1*, *AKT1*, etc. The Clinical Proteomic Tumor Analysis Consortium (CPTAC) breast cancer study^[^ ^11^ ^]^ reported that the proteogenomic landscape of 122 invasive breast cancers provides insights into clinically relevant biology, including cell cycle dysregulation, tumor immunogenicity, aberrant metabolism, and heterogeneity in therapeutic target expression.

Despite this progress, the temporal order of a series of biological events and the driving mechanisms of turning points in BRDC progression remain largely unknown. For example, 1) although genetic mutations, chromosomal region amplifications, and hormone receptor expression levels were known to be associated with BC development, when they first appear in the progression of BRDC and how they drive the progression are unknown. 2) DCIS represents a heterogeneous group that differs in its biological behavior and risk of progression, only a small percentage (14−53%) of cases progress to IDC, resulting in a major clinical challenge in determining which patients to treat.^[^ ^12^ ^]^ Given the increasing concern that a significant number of women with DCIS are overtreated, the identification of patients at very low risk for progression who may forgo surgery and radiation therapy safely and the selective treatment of those at high risk of developing IDC is of significant interest.^[^ ^13^ ^]^ 3) In addition, although the transition from DCIS to IDC is central to the origin of the malignant phenotype, little is known about the time of onset or the triggering mechanism that switches in situ to overt invasive carcinoma in the human breast.^[^ ^14^ ^]^ 4) Although endocrine therapy marked a new era of hormone receptor‐positive IDC (luminal‐type) treatment, a certain proportion of patients present with resistance to drug therapy, making it much more difficult to control the deterioration of the disease.^[^ ^15^ ^]^ Therefore, sorting out novel therapeutic targets and potential therapeutic agents can help solve the problem of drug resistance in some patients. A comprehensive understanding of the progression of BRDC can help to provide strategies for precise treatment of BRDC in different stages.

In this study, we performed integrated multi‐omics analyses using genomic (*n = *79), transcriptomic (*n = *42), proteomic (*n = *224), and phosphoproteomic (*n = *49) data collected from 224 samples from 168 patients with malignant and benign breast diseases. Our data revealed the characteristics of the linear multi‐step progression of BRDC and provided a resource to explore the temporal order of a series of biological events in the progression of BRDC. In addition, we identified potential therapeutic agents, aspirin and dydrogesterone, that target aldo‐keto reductase family 1 member C1 (AKR1C1) and demonstrated their inhibitory effect on tumor cells. In summary, our study helps to understand the progression of BRDC and provides an opportunity to treat BRDC in different stages.

## 2 Results

### 2.1 Proteogenomic Profiling of BRDC Progression

**Figure 1:**
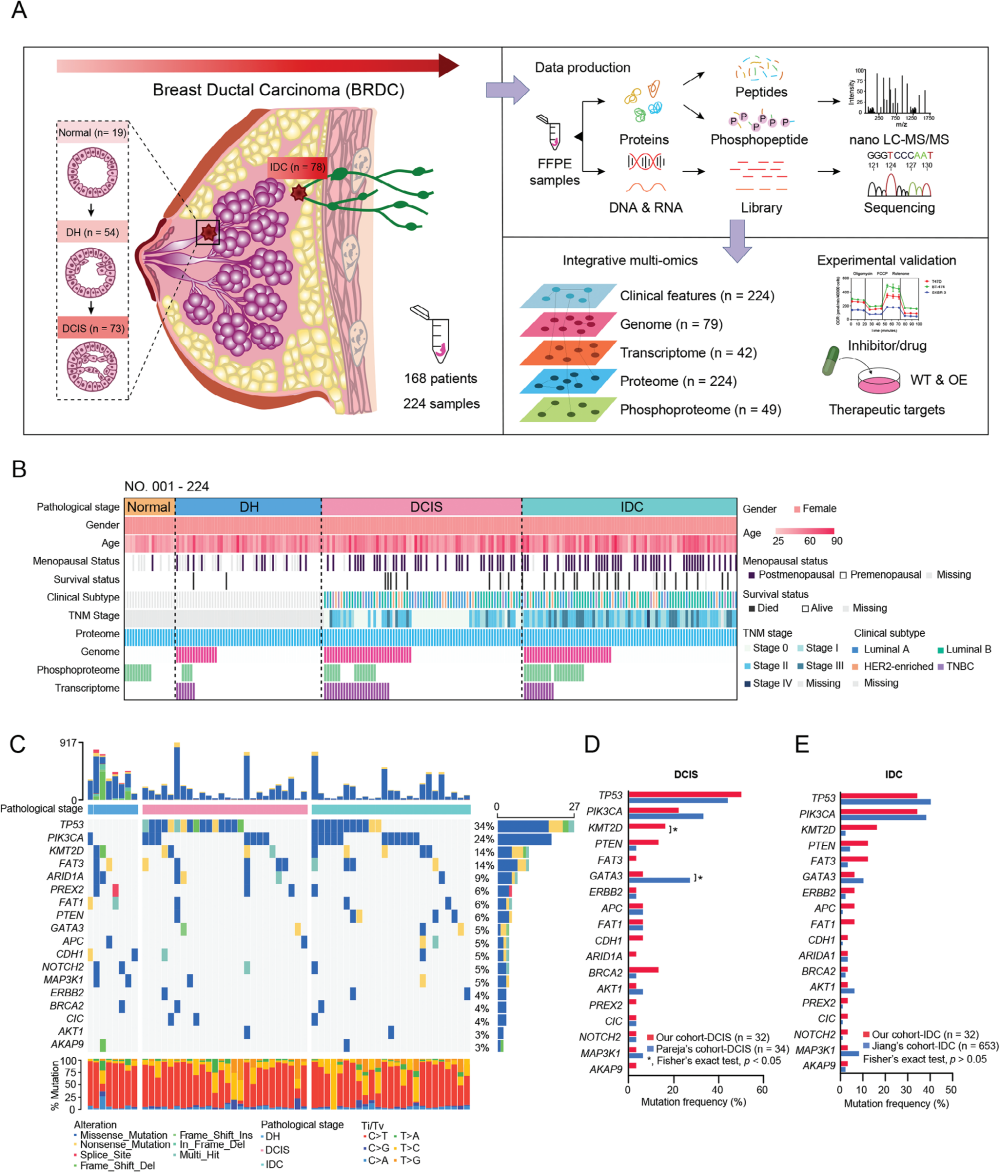
Proteogenomic Profiling of BRDC Progression. A) Overview of the experimental design and the number of samples for proteomics, phosphoproteomics, WES, and RNA‐seq analyses. B) Clinical parameters of the study cohort are indicated in the heatmap. See also Table S1. C) Genetic profile and associated pathological stage of all 79 BRDC progression samples. The bar plot on the top indicates the total number of somatic mutations in each patient. The bar plot at the right represents the distribution and compositions of mutation types in each gene. D) Comparison of frequently mutated genes of DCIS between Pareja's cohort and our cohort. Fisher's exact test, *p < 0.05. E) Comparison of frequently mutated genes of IDC between Jiang's cohort and our cohort. Fisher's exact test, *p* > 0.05.

The present study collected a total of 224 samples from 168 treatment‐naive female patients with malignant and benign breast diseases. Subsequently, 224 samples were classified into 4 progression stages in our cohort by three expert pathologists, including normal ductal epithelial tissue (Normal, *n = *19), ductal hyperplasia (DH, *n = *54), ductal carcinoma in situ (DCIS, *n = *73), and invasive ductal carcinoma (IDC, *n = *78) (Figure S1A, Supporting Information). DH samples included usual ductal hyperplasia (UDH, *n = *37) and atypical ductal hyperplasia (ADH, *n = *17) (Figure S1B, Supporting Information). A schematic of the experimental design is shown in **Figure** **1**A. Clinical information of 168 female patients including age, histological stage, degree of differentiation, TNM stage (AJCC cancer staging system 8th edition), menopausal status, clinical subtype, and status of survival are summarized in Figure 1B and Table S1, Supporting Information.

Breasts are composed of lobules, ducts, adipose, and fibroglandular tissues.^[^ ^16^ ^]^ Histopathologically, the progression of human BRDC was a linear multi‐step process that initiates as normal ductal epithelial tissue lesions, progresses to DCIS, and evolves into IDC. Given the unique nature of adipose tissue in the breast and the different composition of the proteome, breast normal tissues (Normal) were normal ductal epithelial tissues from 19 patients with mammary benign disease in our study. For accurate sampling, hematoxylin and eosin (H&E) stained slides were reviewed and evaluated independently by three expert pathologists, and the areas of Normal and BRDC samples were delineated (Figure S1A, Supporting Information). In addition, when the pathologist evaluated DCIS samples, all DCIS samples were stained with a panel of myoepithelial markers (Calcium‐binding protein (Calponin), tumor protein P63 (P63), and smooth muscle myosin heavy chain (SMMHC)) immunostaining, and the results confirmed that the myoepithelium was intact (Figure S1C, Supporting Information). Further, under the guidance of a pathologist, laser capture microdissection (LCM)^[^ ^17^ ^]^ was applied to dissect the sections of samples precisely, which is generally used to improve the purity (Figure S1D,E, Supporting Information).^[^ ^18^ ^]^

To further rule out interference of breast adipose tissue in Normal samples, we reviewed proteomic data from our cohort. We obtained adipose tissue‐specific proteomic data from the Human Protein Atlas (HPA) (https://www.proteinatlas.org), and adiponectin (ADIPOQ), putative aquaporin‐7‐like protein 3 (AL845331.1), cell death inducing DFFA like effector a (CIDEA), cell death inducing DFFA like effector c (CIDEC), fatty acid binding protein 4 (FABP4), leptin (LEP), lipase E (LIPE), perilipin 1 (PLIN1), and trafficking regulator of GLUT4 (TRARG1) were defined as breast adipose tissue‐specific proteins in the HPA dataset. Among them, AL845331.1, CIDEC, and LEP were not identified at all stages. CIDEA was identified in IDC, but not in Normal, DH, and DCIS (Figure S1E,F, Supporting Information). The protein abundances of FABP4, LIPE, ADIPOQ, PLIN1, TRARG1, and CIDEA in different stages were comparable, respectively. There was no significant difference in the abundance of breast adipose tissue‐specific proteins in Normal samples compared with other stages of BRDC samples (Figure S1E,F, Supporting Information). These results showed that the proteome of the Normal samples was not disturbed by breast adipose tissue.

Additionally, previously published BC studies (TCGA cohort,^[^ ^9^ ^]^ CPTAC cohort,^[^ ^11^, ^19^ ^]^ and FUSCC cohort^[^ ^20^ ^]^) focused on invasive breast cancer, while our cohort included more pre‐invasive and advanced breast cancer. Proteomics analysis was conducted on 224 samples (Normal (*n = *19), DH (*n = *54), DCIS (*n = *73), and IDC (*n = *78)) using a label‐free quantification strategy based on mass spectrometry (MS).^[^ ^21^ ^]^ A phosphoproteomics analysis was conducted on 49 samples (Normal (*n = *10), DH (*n = *4), DCIS (*n = *14), and IDC (*n = *21)) using a Fe‐NTA enrichment strategy. Whole‐exome sequencing (WES) was carried out on 79 samples (DH (*n = *15), DCIS (*n = *32), and IDC (*n = *32)) to detect any possible genetic variants in the cancer genome. In addition, RNA sequencing was carried out on 42 samples (DH (*n = *7), DCIS (*n = *24) and IDC (*n = *11)). This study reveals the progression characteristics of BRDC at the multi‐omics level.

WES data led to a 131.1‐fold mean target coverage and the identification of 14885 genetic variation events. To understand when the known high‐frequency mutation genes of BC first appear in the progression of BRDC and how they drive progress, we mapped 18 high‐frequency mutations (*TP53*, *PIK3CA*, *KMT2D*, *FAT3*, *ARID1A*, *GATA3*, *PREX2*, *APC*, *ERBB2*, *FAT1*, *CDH1*, *NOTCH2*, *PTEN*, *BRCA2*, *CIC*, *MAP3K1*, *AKT1*, and *AKAP9*) (Figure 1C). Notably, the mutation frequency of the *TP53* gene in the entire cohort was 34%, with significant mutations beginning in the DCIS stage (Figure 1C). Compared with Pareja's DCIS cohort,^[^ ^22^ ^]^ we observed that high‐frequency mutations in our DCIS cohort were almost consistent with that in Pareja's cohort, including *TP53* (DCIS cohort in this study versus Pareja's DCIS cohort, 50% versus 44%; *p* > 0.05). (Figure 1D, Table S2, Supporting Information). Compared with Jiang's IDC cohort (*n = *653),^[^ ^23^ ^]^ we also observed that the known high‐frequency mutations of BC in our IDC cohort were almost consistent with that in Jiang's IDC cohort, including *TP53* (IDC cohort in this study versus Jiang's IDC cohort, 34% versus 39.8%; *p* > 0.05) (Figure 1E, Table S2, Supporting Information). These results further demonstrated that genome sequencing data in this study was reliable. To investigate which mutational processes operate in the BRDC progression, we used the sigminer approach^[^ ^24^ ^]^ to categorize mutational signatures. Signatures 30 and 42 were enriched in the DH stage, signatures 2, 6, 7a, and 30 were enriched in the DCIS stage, and signatures 7a, 7b, 10b, and 30 were enriched in the IDC stage (Figure S2A, Supporting Information). Among them, mutational signatures 2, 6, and 30 have been previously reported in breast cancer.^[^ ^7^ ^]^ These results suggested that mutational signatures that have not yet been reported deserve further study.

RNA sequencing (RNA‐seq) analysis identified 12563 genes with fragments per kilobase of transcript per million fragments mapped (FPKM) of more than 1. In addition, we calculated the correlation between 6619, 5488, and 7340 mRNA‐protein pairs for DH, DCIS, and IDC, respectively. The median correlation values of DH, DCIS, and IDC were 0.31, 0.21, and 0.25, respectively (Figure S2B, Supporting Information). This result is similar to that of previous studies investigating clear cell renal cell carcinoma (ccRCC) and urothelial carcinoma (UC).^[^ ^25^, ^26^ ^]^ It is possible that the observed low correlation between mRNA and protein levels could be due to temporal and spatial differences in mRNA and protein synthesis, as well as the low correlation between mRNA and protein half‐lives, and the impact of post‐transcriptional and post‐translational regulation.^[^ ^27^, ^28^ ^]^

For the proteomics and phosphoproteomics data analysis, we used the HEK293T cell line as quality control samples to monitor the MS stability. A Spearman's correlation coefficient was calculated for all quality control runs of HEK293T cell samples. These results showed an average correlation of 0.92 and 0.91 for the proteome and phosphoproteome respectively, demonstrating the consistent stability of the MS platform (Figure S2C,D, Supporting Information). To evaluate the reliability of the results of the cohort samples, all 224 BRDC progression samples were mixed as a BRDC samples pool. These results showed the average correlation coefficient among the repeat runs of the BRDC samples pool was 0.93 and 0.93 for the proteome and phosphoproteome, respectively, suggesting the reliability of the results of the cohort samples (Figure S2E,F, Supporting Information). In addition, we performed the quality assessment analysis of samples, and found that the gradually decreased Spearman's correlation coefficient of the proteomes and phosphoproteomes in four histopathological stages reflected the increased tumor heterogeneity during the carcinogenesis of BRDC, highlighting the importance of exploring molecular characteristics in BRDC progression (Figure S2G,H, Supporting Information). The density plot of the normalized intensities of the proteins and phosphoproteins identified in each sample showed that all samples passed the quality control with an expected unimodal distribution (dip statistic test) (Figure S2I,J, Supporting Information). Label‐free quantification measurement of all patient samples resulted in a total of 15032 protein groups with a 1% false discovery rate (FDR) at the protein and peptide levels (Figure S2K, Supporting Information). In addition, the reference proteome is highly dynamic, spanning about five orders of magnitudes measured by the protein abundances (Figure S2L, Supporting Information). On average, the BRDC proteome had 8178 protein groups per sample, ranging from a minimum of 5970 in DCIS to a maximum of 10323 in IDC (Figure S2M, Supporting Information). Phosphoproteomics analysis was conducted on 49 samples revealing 4256 phosphoproteins and 11 597 phosphosites (Experimental Section). For data analysis, we applied K‐NN imputation to impute the missing values of proteins and phosphorylation sites, which having more than 50% missing data were excluded to ensure that each sample had enough data for imputation (Experimental Section). Our study has established a proteogenomic landscape of BRDC progression.

### 2.2 *TP53* Mutation‐Associated ESR1 Overexpression was Involved in Tumorigenesis of BRDC

**Figure 2:**
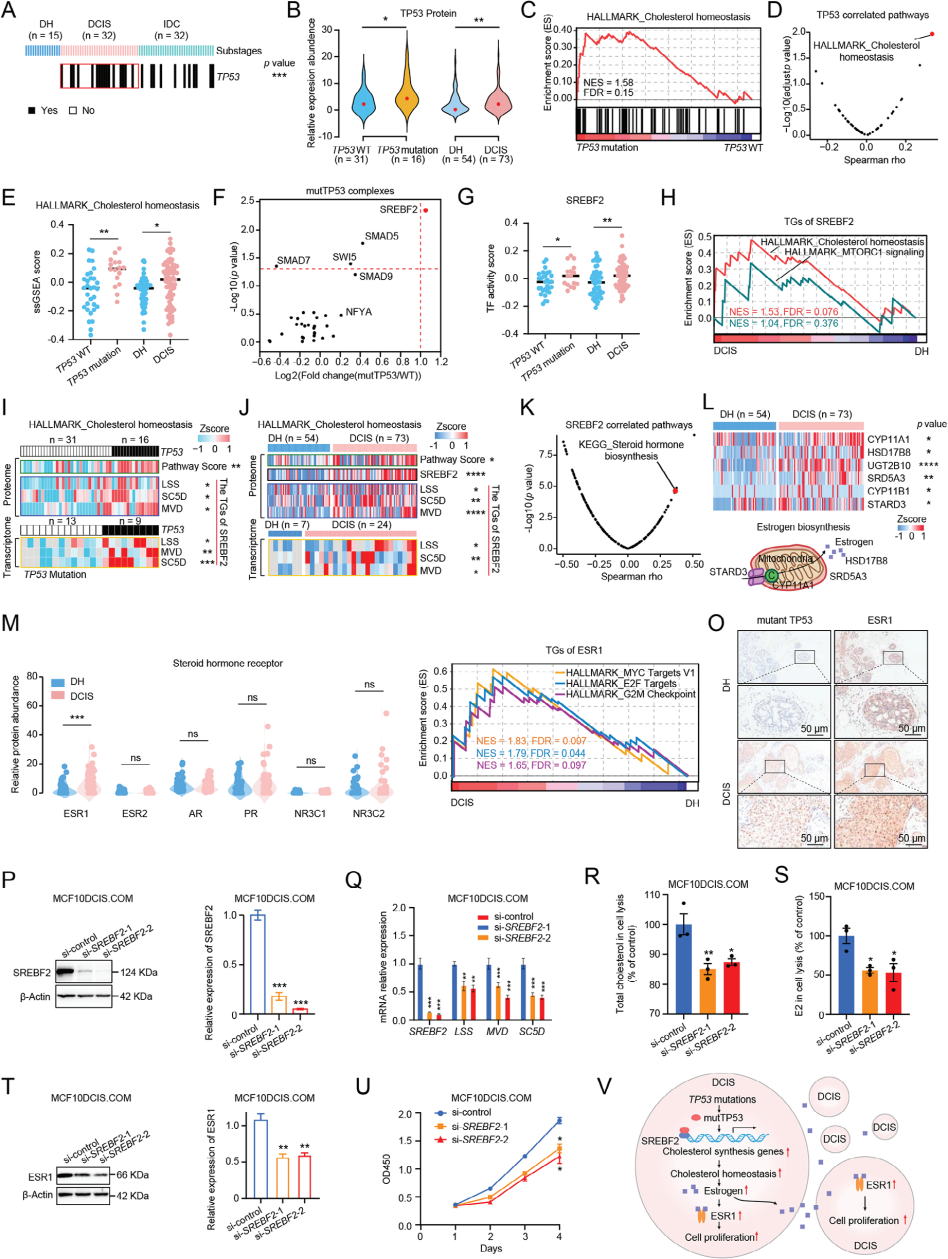
*TP53* Mutation‐Associated ESR1 Overexpression was Involved in Tumorigenesis of BRDC. A) The *TP53* mutation in the different stages of BRDC progression. Fisher's exact test, ***p < 0.001. B) Violin diagram illustrating TP53 protein expression in the *TP53* wild‐type group (*n = *31), the *TP53* mutation group (*n = *16), DH (*n = *54), and DCIS (*n = *73). Student's *t*‐test, *p < 0.05, **p < 0.01. C) Gene set enrichment analysis (GSEA) analysis of gene signatures of cholesterol homeostasis in the *TP53* mutation group (*n = *16) and the *TP53* wild‐type group (*n = *31). D) Volcano plot showing the correlation between the TP53 expression level and the pathway scores by ssGSEA. The one highlighted in red represents the cholesterol homeostasis pathway. The *p* value was calculated by Spearman's correlation test. E) Comparison of the cholesterol homeostasis pathway scores between the *TP53* wild‐type (*n = *31) and *TP53* mutation (*n = *16) groups, and between DH (*n = *54) and DCIS (*n = *73). Student's *t*‐test, *p < 0.05, ** *p* < 0.01. F) The protein expression levels of mutant TP53 complex between the *TP53* mutation (*n = *16) and *TP53* wild‐type (*n = *31) groups. The one highlighted in red represents SREBF2. G) Comparison of the transcription activity of SREBF2 between the *TP53* wild‐type (*n = *31) and *TP53* mutation (*n = *16) groups, and between DH (*n = *54) and DCIS (*n = *73). Student's *t*‐test, *p < 0.05, ** *p* < 0.01. H) GSEA showed that the TGs of SREBF2 were involved in the cholesterol homeostasis pathway in DCIS. I) Heatmaps showing the pathway scores of the cholesterol homeostasis (green box), the protein expression levels of the TGs of SREBF2 (blue box), and the mRNA levels of the TGs of SREBF2 (yellow box) in the *TP53* mutation and *TP53* wild‐type groups. Student's *t*‐test, *p < 0.05, **p < 0.01, ***p < 0.001. J) Heatmaps showing the pathway scores of the cholesterol homeostasis (green box), the protein expression level of SREBF2 (black box), the protein expression levels of the TGs of SREBF2 (blue box), and the mRNA levels of the TGs of SREBF2 (yellow box) in DCIS and DH. Student's *t*‐test, *p < 0.05, **p < 0.01, ****p < 0.0001. K) Volcano plot showing the correlation between the pathway scores and SREBF2 expression level. The one highlighted in red represents the steroid hormone biosynthesis pathway. The *p* value was calculated by Spearman's correlation test. L) Heatmap showing the protein expression levels of the estrogen synthesis‐related proteins in DH (*n = *54) and DCIS (*n = *73) (upper). Schematic diagram of estrogen synthesis in mitochondria (bottom). Student's *t*‐test, *p < 0.05, **p < 0.01, ****p < 0.0001. M) Violin diagram illustrating the protein expression levels of sterol hormone receptors in DH (*n = *54) and DCIS (*n = *73). Student's *t*‐test, ns, not significant, ***p < 0.001. N) GSEA showed that the TGs of ESR1 were involved in the proliferation‐related pathways in DCIS. O) Representative IHC images of TP53 and ESR1 on DH and DCIS tissues. Scale bars = 50 µm. P) A representative western blot analysis of SREBF2 in *SRBEF2*‐knockdown MCF10DCIS.COM cells (left). Quantified western blot results (right). Student's *t*‐test, ***p < 0.001. Q) Real‐time PCR analysis of *SREBF2*, *LSS*, *MVD*, and *SC5D* in *SRBEF2*‐knockdown MCF10DCIS.COM cells. Student's *t*‐test, **p < 0.01, ***p < 0.001. R) The cholesterol content in cell lysis of *SRBEF2*‐knockdown MCF10DCIS.COM cells. Student's *t*‐test, *p < 0.05, **p < 0.01. S) The E2 content in cell lysis of *SRBEF2*‐knockdown MCF10DCIS.COM cells. Student's *t*‐test, *p < 0.05. T) A representative western blot analysis of ESR1 in *SRBEF2*‐knockdown MCF10DCIS.COM cells (left). Quantified western blot results (right). Student's *t*‐test, **p < 0.01. U) Cell proliferation associated with various treatments (*n = *3 repeats per group). Student's *t*‐test, *p < 0.05. V) A brief model depicting the functional impact of *TP53* mutation‐associated ESR1 Overexpression.

To elucidate the first step in the progression from DH to DCIS, we investigated the neo‐mutations in the progression of BRDC. We found that *TP53* mutation was high‐frequency event of BRDC, and occurred as early as in the tumorigenesis stage with a frequency of 50% in DCIS, whereas no *TP53* mutation was observed in DH (DCIS versus DH, Fisher's exact test, *p* < 0.0001) (**Figures** 1C and **2**A).

Next, the expression of TP53 in the *TP53* mutation group was significantly higher than that in the *TP53* wild‐type group (Figure 2B, Table S3, Supporting Information). Consistently, the expression of TP53 in DCIS was significantly higher than that in DH (Figure 2B, Table S3, Supporting Information). Further, gene set enrichment analysis (GSEA) revealed that cholesterol homeostasis was enriched in the *TP53* mutation group compared with the *TP53* wild‐type group (NES = 1.58, FDR = 0.15) (Figure 2C). In addition, the correlation analysis results showed that the enrichment score of the cholesterol homeostasis pathway had a significantly positive correlation with the expression of TP53 (Spearman rho = 0.33, *p* = 1.21e‐04) (Figure 2D, Table S3, Supporting Information). Pathway scores based on global proteomics data obtained by single‐sample gene set variation analysis (ssGSEA)^[^ ^29^, ^30^ ^]^ demonstrated the cholesterol homeostasis pathway was significantly upregulated in the *TP53* mutation group and DCIS compared with the wild‐type *TP53* group and DH, respectively (Student's *t*‐test, *p* < 0.05) (Figure 2E). Taken together, our results suggested that mutant TP53 may be involved in the regulation of cholesterol homeostasis.

Previous studies suggest that several genes can specifically bind to mutant TP53 and contribute to regulating malignant phenotypes.^[^ ^31^ ^]^ Further, we collected a set of proteins that forms a complex with the mutant TP53, in which the expression of sterol regulatory element binding transcription factor 2 (SREBF2) was significantly higher in the *TP53* mutation group than in the *TP53* wild‐type group (Figure 2F). We further inferred the transcriptional activity of SREBF2 in the *TP53* mutation group and DCIS based on RNA‐seq data using the master regulator inference algorithm (MARINa) compiled in the R package “viper”. The results demonstrated that the transcriptional activities of SREBF2 in the *TP53* mutation group and DCIS were higher than that in the wild‐type *TP53* group and DH, respectively (Figure 2G). To further evaluate the function of transcriptional factor SREBF2 in the *TP53* mutation group and DCIS, we performed GSEA using the target genes (TGs) of SREBF2 from the ENCODE transcription factor targets dataset^[^ ^32^ ^]^ (Table S3, Supporting Information). In agreement with the previous results, GSEA showed enrichment of the cholesterol homeostasis pathway in the *TP53* mutation group (NES = 1.62, FDR = 0.053) and DCIS (NES = 1.53, FDR = 0.076) (Figure 2H and Figure S3A, Supporting Information). From another perspective, we analyzed the relationship between specific transcription factors (TFs) and TGs, and their dynamic changes during the BRDC progression. It is worth mentioning that the specific TFs of DCIS, such as sterol regulatory element binding transcription factor 1 (SREBF1), SREBF2, and estrogen receptor 1 (ESR1) were mainly involved in the steroid metabolic process (Figure S3B, Supporting Information). Subsequently, we found that compared with the wild‐type *TP53* group and DH, cholesterol biosynthesis‐related proteins ((lanosterol synthase (LSS), sterol‐C5‐desaturase (SC5D), and mevalonate diphosphate decarboxylase (MVD)) were upregulated in the *TP53* mutation group and DCIS both in mRNA and protein levels (Student's *t*‐test, *p* < 0.05) (Figure 2I,J). These results showed that mutant TP53 was involved in the regulation of cholesterol homeostasis together with SREBF2 in DCIS.

The cellular cholesterol level reflects the dynamic balance between biosynthesis, transport, and esterification—a process in which cholesterol is converted to bile acids, vitamins, and steroid hormones eventually.^[^ ^33^, ^34^, ^35^ ^]^ The correlation analysis results showed a significantly positive correlation between SREBF2 and the steroid hormone biosynthesis enrichment score (Spearman rho = 0.37, *p* = 2.35e‐05), suggesting that synthetic cholesterol may be metabolized into steroid hormones in DCIS (Figure 2K). Subsequently, we found that estrogen synthesis‐related proteins were upregulated in the *TP53* mutation group and DCIS (Figure 2L and Figure S3C, Supporting Information). These results suggested that intracellular cholesterol may be transported to mitochondria via StAR‐related lipid transfer domain containing 3 (STARD3), and metabolized as estrogen (Figure 2L).^[^ ^34^ ^]^ It was another piece of evidence, among 6 steroid hormone receptors (ESR1, estrogen receptor 2 (ESR2), androgen receptor (AR), progesterone receptor (PGR), nuclear receptor subfamily 3 group C member 1 (NR3C1), and nuclear receptor subfamily 3 group C member 2 (NR3C2)), only ESR1 activity and expression level in the *TP53* mutation group and DCIS were higher than that in the wild‐type *TP53* group and DH (Student's *t*‐test, *p* < 0.05) (Figure 2M and Figure S3D, Supporting Information). To further evaluate the function of transcriptional factor ESR1 in DCIS, we performed GSEA using the TGs of ESR1 from the CHEA transcription factor targets dataset^[^ ^32^ ^]^ (Table S3, Supporting Information). We found that cell proliferation‐related pathways were upregulated in the wild‐type *TP53* group and DCIS (Figure 2N and Figure S3E, Supporting Information). Furthermore, the multigene proliferation score (MGPS) of the *TP53* mutation group and DCIS was significantly higher than that of the wild‐type *TP53* group and DH (Student's *t*‐test, *p* < 0.05) (Figure S3F, Supporting Information). In addition, we used phosphoproteomic data to analyze the relationship between kinases and substrates, and their dynamic changes during the BRDC progression. It is worth mentioning that the upregulated kinases in DCIS (DCIS versus Normal, *p* < 0.05), such as calcium dependent protein kinase 2 alpha (CAMK2A), cyclin dependent kinase 2 (CDK2), mitogen‐activated protein kinase 1 (MAPK1), P21 activated kinase (PAK4), protein kinase AMP‐activated catalytic subunit aipha 2 (PRKAA2), protein kinase CAMP‐activated catalytic subunit alpha (PRKACA), were mainly participated in the cell cycle, while the upregulated kinases in DH and IDC were mainly involved in response to hormone and regulation of cytoskeleton organization, respectively (Figure S3G, Supporting Information). The above findings reflected the consistency of phosphorylation signal transduction and their functional enrichment. Among these, a specific upregulated kinase in DCIS (DCIS versus Normal, *p* < 0.05, DH versus Normal, *p* > 0.05, IDC versus Normal, *p* > 0.05), PRKAA2, was a target of an approved inhibitor (Figure S3H, Supporting Information). In addition, we examined the expression levels of mutant TP53 and ESR1 in our cohort by immunohistochemistry (IHC). It was confirmed that the expression of mutant TP53 and ESR1 in DCIS was higher than that in DH (Student's *t*‐test, *p* < 0.05) (Figure 2O and Figure S3I, Supporting Information).

To verify whether SREBF2 is involved in cholesterol synthesis and thus regulates ESR1 expression, the siRNA against *SRBEF2* was applied to knock down the expression of SRBEF2 in MCF10DCIS.COM cells. The efficiency of *SRBEF2* knockdown cells was verified through mRNA and protein expression levels (Figure 2P,Q). Next, we detected the transcriptional levels of the cholesterol synthesis genes (LSS, SC5D, and MVD). These results showed that compared with wild‐type MCF10DCIS.COM cells, the transcriptional levels of the cholesterol synthesis‐related proteins (LSS, SC5D, and MVD) in *SRBEF2‐*knockdown MCF10DCIS.COM cells were significantly down‐regulated (Figure 2Q). To verify the effect of *SREBF2* knockdown on cholesterol and its downstream estrogen synthesis levels, we detected the synthesis level of cholesterol and its downstream estrogen in *SRBEF2*‐knockdown MCF10DCIS.COM cells by ELISA assay. We found that the synthesis levels of cholesterol and its downstream estrogen were decreased in MCF10DCIS.COM cells after *SREBF2* knockdown (Figure 2R,S). These results suggested that SREBF2 was involved in the synthesis of cholesterol and its downstream estrogen. It has been reported that estrogen can enhance the expression of ESR1.^[^ ^36^ ^]^ Further, we found that compared with wild‐type MCF10DCIS.COM cells, the expression level of ESR1 was significantly down‐regulated in *SRBEF2*‐knockdown MCF10DCIS.COM cells (Figure 2T). In addition, compared with wild‐type MCF10DCIS.COM cells, we found that the cell proliferation of *SRBEF2*‐knockdown MCF10DCIS.COM cells was significantly inhibited (Figure 2U). These results further supported our conclusion, SRBEF2 activation leads to an increase in cholesterol synthesis genes and consequently the rise of ESR1, and further promotes cell proliferation.

Altogether, these results suggested that *TP53* mutation increases ESR1 activity and expression, and further promotes cell proliferation, resulting in carcinogenesis (Figure 2V).

### 2.3 6q21 Amplification‐Associated NR3C1 Overexpression was Involved in Immune Escape of Tumor Cells in DCIS_Pure

DCIS represents a heterogeneous group that differs in its biological behavior and risk of progression, only a small percentage (14−53%) of cases progress to IDC. The histological similarities lead to a major clinical challenge in determining which patients to treat.^[^ ^12^ ^]^ To clarify the heterogeneity of DCIS, combined with the histopathological information, we divided the 73 DCIS samples in our cohort into two subgroups: DCIS_Pure (pure DCIS, no histologic evidence of invasion) (*n = *30) and DCIS_adjIDC (DCIS adjacent to IDC, DCIS regions of invasive cancers) (*n = *43) (Figure S4A, Supporting Information). We showed representative hematoxylin and eosin (H&E)‐stained slides of DCIS_Pure and DCIS_adjIDC samples (Figure S4B, Supporting Information). As for the histological grade, there was no significant difference between the DCIS_Pure and DCIS_adjIDC subgroups (Figure S4C, Supporting Information). Interestingly, principal component analysis (PCA) of transcriptomic, proteomic, and phosphoproteomic data separated DCIS_Pure, DCIS_adjIDC, and IDC samples (Figure S4D‐F, Supporting Information), revealed the molecular differences among them.

**Figure 3:**
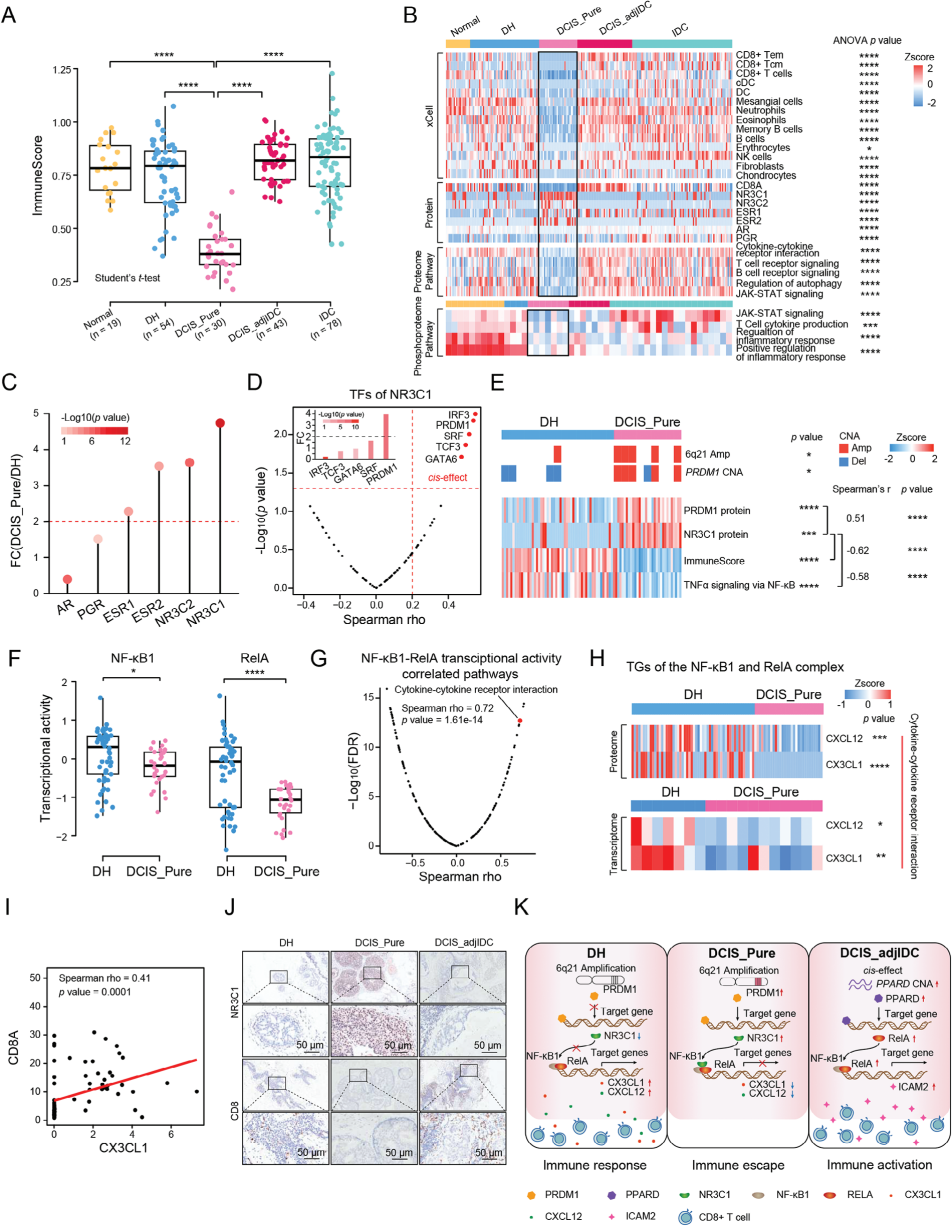
6q21 Amplification‐Associated NR3C1 Overexpression was Involved in Immune Escape of Tumor Cells in DCIS_Pure. A) Boxplot illustrating the immune scores across different stages of BRDC progression: Normal (*n = *19), DH (*n = *54), DCIS_Pure (*n = *30), DCIS_adjIDC (*n = *43), and IDC (*n = *78). The box represents the interquartile range (IQR) from the first (Q1) to the third quartile (Q3) of the distribution, and the line inside the box represents the median. The whiskers extend from Q1 to Q3 to the endpoints, which are defined as the most extreme data points within Q1 − 1.5 × IQR and Q3 + 1.5 × IQR, respectively. Student's *t*‐test, ****p < 0.0001. B) Heatmap illustrating cell type compositions and activities of selected individual genes/proteins and pathways across the BRDC progression stages. The heatmap in the first section illustrated the immune/stromal signatures from xCell. The heatmap in the second section illustrates the protein abundances of sterol hormone receptors and immune‐related marker CD8A. ssGSEA scores based on global proteomic and phosphoproteomic data for biological pathways upregulated in different progression stages of BRDC are illustrated in the remaining sections. ANOVA test, *p < 0.05, ***p < 0.001, ****p < 0.0001. C) Comparison of the protein expression levels of sterol hormone receptors between the DH (*n = *54) and DCIS_Pure (*n = *30). The *p* value was calculated by Student's *t*‐test. D) Volcano plot showing the correlation between the protein expression levels of NR3C1 TFs and their corresponding CNA. The red points show the *cis*‐effect TFs (Spearman's rho > 0.2, *p* < 0.05). Histogram showing the fold changes of the protein expression levels of the *cis*‐effect TFs in DCIS_Pure compared with DH. Student's *t*‐test (upper left corner). E) Heatmaps showing copy number alteration (CNA) of *PRDM1*, the protein abundances of PRDM1 and NR3C1, the immune score, and the pathway score of TNF‐α signaling via NF‐ĸB in DH and DCIS_Pure. Student's *t*‐test, *p < 0.05, ***p < 0.001, ****p < 0.0001. F) Boxplot showing the transcriptional activities of NF‐ĸB1 and RelA in DH (*n = *54) and DCIS_Pure (*n = *30). Student's *t*‐test, *p < 0.05, ****p < 0.0001. G) Volcano plot showing the correlations between the transcription factor activity of NF‐ĸB1 and RelA and the pathway scores by ssGSEA. The one highlighted in red represents the pathway of cytokine‐cytokine receptor interaction (Spearman's rho = 0.72, *p* = 1.61e‐14). H) Heatmaps showing the protein expression levels of CXCL12 and CX3CL1 (upper) and the mRNA levels of CXCL12 and CX3CL1 (bottom) in DH and DCIS_Pure. Student's *t*‐test, *p < 0.05, **p < 0.01, ***p < 0.001, ****p < 0.0001. I) Spearman‐rank correlation of the protein expression levels of CX3CL1 and CD8A. J) Representative IHC images of NR3C1 and CD8 on DH, DCIS_Pure, and DCIS_adjIDC tissues. Scale bars = 50 µm. K) The paradigm of the immune alteration among the DH, DCIS_Pure, and DCIS_adjIDC.

Next, the tumor microenvironment component in our cohort was studied using xCell based on proteomic data. Notably, DCIS_Pure displayed the lowest immune score (**Figure** **3**A; Table S4, Supporting Information). In DCIS_Pure, the abundance of CD8+ Tem, CD8+ Tcm, and CD8+ T cells was at rock bottom, which was consistent with the expression level of CD8+ T cells marker CD8A (ANOVA test, *p* < 0.0001; DCIS_Pure versus DH, Student's *t*‐test, *p* <0.0001; DCIS_Pure versus DCIS_adjIDC, Student's *t*‐test, *p* <0.0001) (Figure 3B, Table S4, Supporting Information). Proteomics and phosphoproteomics analysis showed that inflammatory response‐related pathways were significantly upregulated in Normal compared with DH, DCIS, and IDC, respectively (Student's *t*‐test, *p* < 0.05) (Figure 3B). These results were consistent with the results of Aran et al.,^[^ ^37^ ^]^ suggesting that our phosphoproteome sequencing data was reliable. Further, we found that the immune‐related pathways were down‐regulated in DCIS_Pure, including cytokine‐cytokine receptor interaction, T cell receptor signaling, B cell receptor signaling, and JAK‐STAT signaling (ANOVA test, *p* < 0.0001; DCIS_Pure versus DH, Student's *t*‐test, *p* < 0.0001; DCIS_Pure versus DCIS_adjIDC, Student's *t*‐test, *p* < 0.0001) (Figure 3B). These results suggested that DCIS_Pure was in the phase of immune quiescence in BRDC progression.

In contrast, the steroid hormone receptor NR3C1 was at its peak both on the mRNA and protein levels (FC^[DCIS_Pure/DH]^ = 4.7, *p* = 1.75e‐12; FC^[DCIS_Pure/DCIS_adjIDC]^ = 125.8, *p* = 2.72e‐26) (Figure 3B,C, and Figure S4G, Supporting Information). It has been reported that NR3C1 is an important down‐regulator of inflammation.^[^ ^38^, ^39^, ^40^, ^41^ ^]^ It is noteworthy that there was a significant negative correlation between the expression of NR3C1 and immune score (Spearman rho = −0.62, *p* = 2.83e‐10), suggesting that NR3C1 might be involved in immune quiescence in DCIS_Pure (Figure S4H, Supporting Information).

Genomic alterations that affect gene expression levels at the same locus are said to act in *cis*, whereas an impact of another locus is defined as a *trans*‐effect.^[^ ^42^, ^43^, ^44^ ^]^ To pinpoint the dramatic upregulation of NR3C1 in DCIS_Pure, we investigated TFs of NR3C1 from the Transcription Factor Target Gene Database (http://tfbsdb.systemsbiology.net) (Table S4, Supporting Information). A total of 5 TFs (*IRF3*, *PRDM1*, *SRF*, *TCF3*, and *GATA6*) of NR3C1 showed *cis*‐effect between their protein expression level and copy number alteration (CNA), correspondingly (Spearman rho > 0.2, *p* < 0.05) (Figure 3D). Among them, we identified a *cis*‐effect of PR‐domain containing 1 (PRDM1) in 6q21 (Spearman rho = 0.54, *p* = 6.59e‐3), and its protein expression level in DCIS_Pure was higher than in DH (FC^[DCIS_Pure/DH]^ = 3.9, *p* = 1.8e‐4) (Figure 3D,E). Furthermore, we found a significant positive correlation between the protein expression of NR3C1 and PRDM1 (Spearman rho = 0.51, *p* = 7.29e‐7) (Figure 3E).

To further explore how NR3C1 was involved in immune silencing in DCIS_Pure, we first performed a correlation analysis. The results showed that the enrichment score of the TNF‐*α* signaling via the NF‐κB pathway had a significantly negative correlation with the expression of NR3C1 (Spearman rho = −0.58, *p* = 8.0e‐9) (Figure S4I, Supporting Information). The transcriptional activity analysis showed that the transcriptional activities of nuclear factor kappa B subunit 1 (NF*κ*B1) and nuclear factor NF‐kappa‐B P65 subunit (RelA) in the TNF‐*α* signaling via NF‐κB pathway were decreased significantly in DCIS_Pure (Figure 3F). The transcriptional activity score of NF*κ*B1 and RelA complex showed a significant positive correlation with the immune score (Figure S4J, Supporting Information) (Spearman rho = 0.66, *p* = 6.51e‐12) and the enrichment score of cytokine‐cytokine receptor interaction pathway (Spearman rho = 0.72, *p* = 1.62e‐14) (Figure 3G and Figure S4K, Supporting Information). The cytokine‐cytokine receptor interaction pathway was downregulated in DCIS_Pure (Figure S4L, Supporting Information). Further, cytokines C‐X‐C motif chemokine ligand 12 (CXCL12) and C‐X3‐C motif chemokine ligand 1 (CX3CL1), the TGs of NF*κ*B1 and RelA, were significantly downregulated both on the mRNA and protein levels in DCIS_Pure (Figure 3H). Previous studies have reported that CXCL12 and CX3CL1 could chemotaxis CD8+ T cells.^[^ ^45^, ^46^, ^47^, ^48^, ^49^ ^]^ In our proteomic data, CXCL12 and CX3CL1 were significantly positively correlated with CD8 subunit alpha (CD8A) (Figure 3I and Figure S4M‐O, Supporting Information), and CD8A was downregulated in DCIS_Pure (Figure 3B and FIgure S4P, Supporting Information).

Interestingly, we observed that the immune score was increased in DH and DCIS_adjIDC than that in DCIS_Pure, suggesting the immune escape in DCIS_Pure and immune activation in DCIS_adjIDC (Figure 3A). To explore why the immune activation in DCIS_adjIDC, we performed the *cis*‐effect analysis and found *PPARD* was the cis‐effect gene, leading to the protein (peroxisome proliferator‐activated receptor delta, PPARD) encoded by PPARD was up‐regulated in DCIS_adjIDC compared with DH (Figure S4Q, Supporting Information). Notably, PPARD, a transcription factor, was involved in immune regulation. It was reported that RelA was the TG of PPARD. In our dataset, we observed that the expression level of RelA had a significantly positive correlation with the expression level of PPARD (Figure S4R, Supporting Information). Consistently, the transcriptional level of RelA in DCIS_adjIDC was higher than that in DH (Figure S4S, Supporting Information). It was reported that intercellular adhesion molecule 2 (ICAM2) was the TG of RelA. In our dataset, we observed that the expression level of ICAM2 had a significantly positive correlation with the expression level of RelA (Figure S4T, Supporting Information). Further, the expression level of ICAM2 was up‐regulated in DCIS_adjIDC compared with DH (Figure S4U, Supporting Information). In addition, the expression level of ICAM2 had a significantly positive correlation with the expression level of CD8A, suggesting that ICAM2‐mediated adhesive interactions are important for immune activation in DCIS_adjIDC (Figure S4V, Supporting Information). These results indicated the genomic alteration impacts the immune microenvironment in DCIS_adjIDC by activating PPARD and facilitating the transcription of the immune‐related genes.

On the other hand, the IHC evidenced that the abundance of CD8+ T cells was lower in DCIS_Pure than in DH and DCIS_adjIDC, while the expression level of NR3C1 was higher in DCIS_Pure than in DH and DCIS_adjIDC (Figure 3J and Figure S4W, Supporting Information). In conclusion, from DH to DCIS_Pure, 6q21 gain increases PRDM1 expression and further upregulate NR3C1 expression, resulting in the downregulation expression of CX3CL1 and CXCL12 by inhibiting the pathway of TNF‐*α* signaling via NF*κ*B. The downregulated expression of CX3CL1 and CXCL12 leads to the reduction of their chemotaxis to CD8+ T cells, thus resulting in immune escape of tumor cells in DCIS_Pure (Figure 3K).

**Figure 4:**
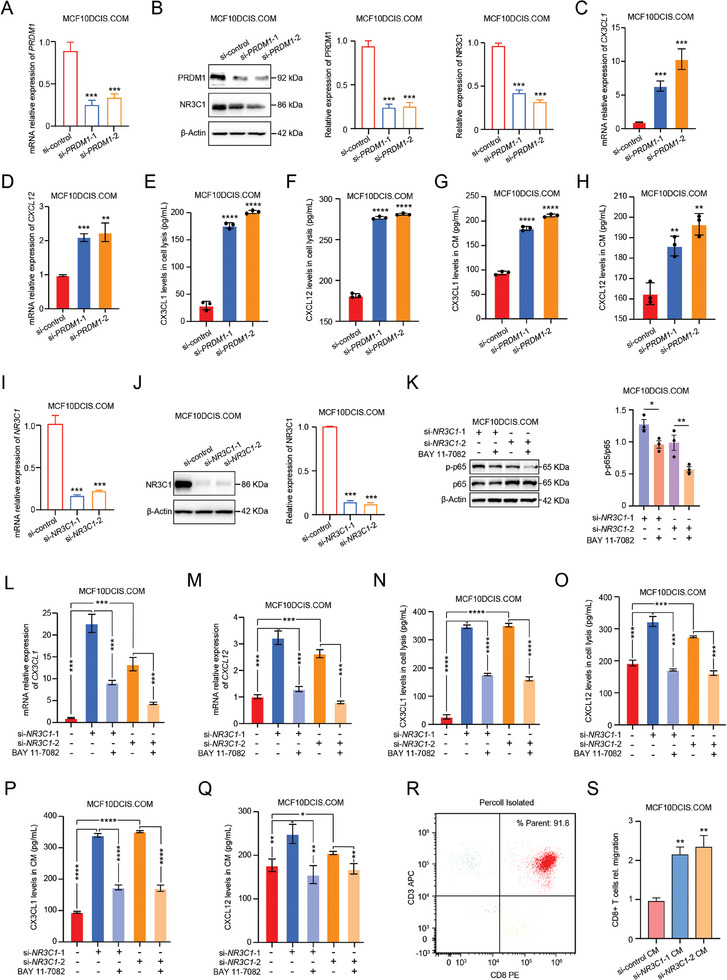
NR3C1 overexpression was involved in immune escape of tumor cells. A) Real‐time PCR analysis of *PRDM1* in *PRDM1*‐knockdown MCF10DCIS.COM cells. Student's *t*‐test, ***p < 0.001. B) A representative western blot analysis of PRDM1 and NR3C1 in *PRDM1*‐knockdown MCF10DCIS.COM cells (left). Quantified western blot results (middle and right). Student's *t*‐test, ***p < 0.001. C,D) Real‐time PCR analysis of *CX3CL1* and *CXCL12* in *PRDM1*‐knockdown MCF10DCIS.COM cells. Student's *t*‐test, **p < 0.01, ***p < 0.001. E,F) ELISA analysis of the protein levels of CX3CL1 and CXCL12 in cell lysate supernatant of *PRDM1*‐knockdown MCF10DCIS.COM cells. Student's *t*‐test, ****p < 0.0001. G,H) ELISA analysis of the protein levels of CX3CL1 and CXCL12 in CM of *PRDM1*‐knockdown MCF10DCIS.COM cells. Student's *t*‐test, ****p < 0.0001. I) Real‐time PCR analysis of *NR3C1* in *PRDM1*‐knockdown MCF10DCIS.COM cells. Student's *t*‐test, ***p < 0.001. J) A representative western blot analysis of NR3C1 in *NR3C1*‐knockdown MCF10DCIS.COM cells (left). Quantified western blot results (middle and right). Student's *t*‐test, ***p < 0.001. K) A representative western blot analysis of p‐p65 in *NR3C1*‐knockdown MCF10DCIS.COM cells or treatment with 10 µm BAY 11–7082 (left). Quantified western blot results (right). Student's *t*‐test, *p < 0.05, **p < 0.01. L,M) Real‐time PCR analysis of *CX3CL1* and *CXCL12* in *NR3C1*‐knockdown MCF10DCIS.COM cells or treatment with 10 µm BAY 11–7082. Student's *t*‐test, ***p < 0.001. N,O) ELISA analysis of the protein levels of CX3CL1 and CXCL12 in cell lysate supernatant of *NR3C1*‐knockdown MCF10DCIS.COM cells or treatment with 10 µm BAY 11–7082. Student's *t*‐test, ***p < 0.001, ****p < 0.0001. P,Q) ELISA analysis of the protein levels of CX3CL1 and CXCL12 in CM of *NR3C1*‐knockdown MCF10DCIS.COM cells or treatment with 10 µm BAY 11–7082. Student's *t*‐test, *p < 0.05, ****p < 0.0001. R) The purity of the isolated CD8+ T cells from PBMCs of BC patients were determined by flow cytometry. S) Two‐chamber migration of CD8+ T cells recruited by CM of *NR3C1*‐knockdown MCF10DCIS.COM cells for 12 h. Student's *t*‐test, **p < 0.01.

To verify whether PRDM1 regulates the NR3C1 expression, the siRNA against *PRDM1* was applied to knock down the expression of PRDM1 in MCF10DCIS.COM cells. The efficiency of *PRDM1* knockdown cells was verified through mRNA and protein expression levels (**Figure** **4**A,B). To verify whether PRDM1 regulates the expression of NR3C1, we detected the expression level of NR3C1. We found that the expression level of NR3C1 was significantly decreased after *PRDM1* knockdown (Figure 4B). These results suggested that *PRDM1* knockdown reduced the expression level of NR3C1. Next, to verify the effect of *PRDM1* knockdown on the expression of levels CX3CL1 and CXCL12, we detected the transcriptional levels and protein expression levels of CX3CL1 and CXCL12. These results showed that compared with wild‐type MCF10DCIS.COM cells, the transcriptional levels and protein expression of CX3CL1 and CXCL12 were significantly up‐regulated in *PRDM1*‐knockdown MCF10DCIS.COM cells (Figure 4C–F). In addition, we detected the release levels of CX3CL1 and CXCL12 in *PRDM1*‐knockdown MCF10DCIS.COM cells. We found that the expression levels of CX3CL1 and CXCL12 were increased in the culture medium (CM) after *PRDM1* knockdown (Figure 4G,H).

To verify NR3C1 regulates the down‐regulated expression of CX3CL1 and CXCL12 by inhibiting the pathway of TNF‐α signaling via NF‐κB, the siRNA against *NR3C1* was applied to knock down the expression of NR3C1 in MCF10DCIS.COM cells. The efficiency of *NR3C1* knockdown cells was verified on the mRNA and protein levels (Figure 4I,J). In addition, we observed that the NF‐κB signaling was down‐regulated in *NR3C1*‐knockdown MCF10DCIS.COM cells after treatment with NF‐κB inhibitor BAY 11–7082 (10 µm), as shown by p65 phosphorylation levels (Figure 4K). Next, we detected the transcriptional levels and protein expression levels of CX3CL1 and CXCL12. These results showed that compared with wild‐type MCF10DCIS.COM cells, the transcriptional levels and protein expression levels of CX3CL1 and CXCL12 were significantly up‐regulated in *NR3C1*‐knockdown MCF10DCIS.COM cells (Figure 4L–O). However, the transcriptional levels and protein expression levels of CX3CL1 and CXCL12 were significantly down‐regulated in *NR3C1*‐knockdown MCF10DCIS.COM cells after treatment with BAY 11–7082 (10 µm), suggesting that the up‐regulation of CX3CL1 and CXCL12 caused by the knockdown of *NR3C1* was diminished by the NF‐κB inhibitor BAY 11–7082 (Figure 4L–O). In addition, we detected the release levels of CX3CL1 and CXCL12 in *NR3C1*‐knockdown MCF10DCIS.COM cells. We found that the expression levels of CX3CL1 and CXCL12 were increased in CM after *NR3C1* knockdown (Figure 4P,Q). However, we found that the release levels of CX3CL1 and CXCL12 were decreased in CM of *NR3C1*‐knockdown MCF10DCIS.COM cells after treatment with BAY 11–7082 (10 µm) (Figure 4P,Q). These results further approved that NR3C1 down‐regulated the expression levels of CX3CL1 and CXCL12 by inhibiting the pathway of TNF‐α signaling via NF‐κB.

In addition, previous studies have reported that CXCL12 and CX3CL1 could chemotaxis CD8+ T cells.^[^ ^45^, ^46^, ^47^, ^48^, ^49^ ^]^ In our proteomic data, CXCL12 and CX3CL1 were significantly positively correlated with CD8A, suggesting that CXCL12 and CX3CL1 may be chemotaxis CD8+ T cells. To further verify the above hypothesis, we isolated CD8+ T cells from the peripheral blood mononuclear cells (PBMCs) of BC patients. The purity of the isolated CD8+ T cells was determined by flow cytometry (Figure 4R). Then, we performed two‐chamber CD8+ T cells migration assays. We found that compared with the CM of wild‐type MCF10DCIS.COM cells, the *NR3C1*‐knockdown MCF10DCIS.COM cells CM were able to attract more CD8+ T cells (Figure 4S). These results further approved that the downregulated expression of CX3CL1 and CXCL12 leads to the reduction of their chemotaxis to CD8+ T cells, thus resulting in immune escape of tumor cells in DCIS_Pure.

### 2.4 Proteogenomic Analysis Indicated the TIAM1‐AR‐AKR1C1 Axis Promoted Cell Invasion and Migration in DCIS_adjIDC

**Figure 5:**
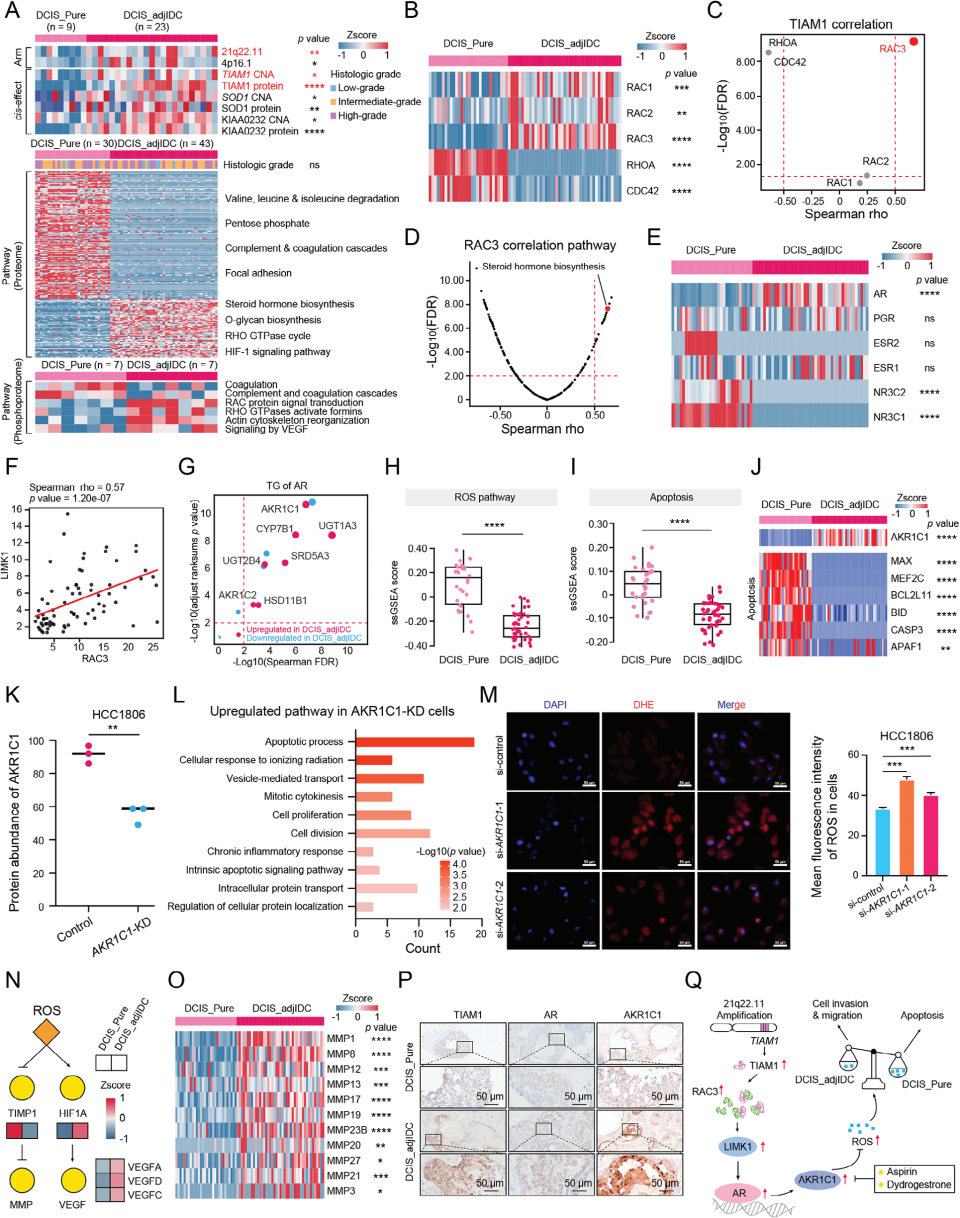
Proteogenomic Analysis Indicated the TIAM1‐AR‐AKR1C1 Axis Promoted Cell Invasion and Migration in DCIS_adjIDC. A) Heatmap depicting the multi‐omics alteration between DCIS_Pure and DCIS_adjIDC. The top panel showed the significant focal‐level CNA events and *cis*‐effect genes on the above focal between DCIS_Pure and DCIS_adjIDC (Rank sums test, *p < 0.05, **p < 0.01, ****p < 0.0001). The middle and bottom panels show the significantly enriched pathway and its participated genes between DCIS_Pure and DCIS_adjIDC (Rank sums test, *p* < 0.05). B) Heatmap showing the protein expression of Rho‐like GTPases between DCIS_Pure (*n = *30) and DCIS_adjIDC (*n = *43). Rank sums test, **p < 0.01, ***p < 0.001, ****p < 0.0001. C) The Spearman's correlation coefficient between guanylate exchange factor TIAM1 and Rho‐like GTPases. The one highlighted in red represents RAC3. D) Volcano plot showing the correlation between the RAC3 expression level and the pathway scores by ssGSEA. The one highlighted in red represents the steroid hormone biosynthesis pathway. The *p* value was calculated by Spearman's correlation test. E) Heatmap depicting the protein expression levels of sterol hormone receptors in DCIS_Pure (*n = *30) and DCIS_adjIDC (*n = *43). Rank sums test, ns, not significant, ****p < 0.0001. F) Spearman‐rank correlation of the protein expression levels of RAC3 and its downstream LIMK1. G) The TGs of AR. The x‐axis indicates the significance of the correlation between AR and its TG, and the y‐axis denotes the *p* value of the protein expression levels of TGs from Rank sums test in DCIS_adjIDC compared with DCIS_Pure. The red dots represent the up‐regulated proteins in DCIS_adjIDC, and the blue dots represent the down‐regulated proteins in DCIS_adjIDC. The dot size indicates the fold change of the protein expression levels in DCIS_adjIDC compared with DCIS_Pure. H) Comparison of the ROS pathway scores DCIS_Pure (*n = *30) and DCIS_adjIDC (*n = *43). Rank sums test, ****p < 0.0001. I) Comparison of the apoptosis pathway scores DCIS_Pure (*n = *30) and DCIS_adjIDC (*n = *43). Rank sums test, ****p < 0.0001. J) Heatmap showing the expression levels of apoptosis‐related proteins in DCIS_Pure (*n = *30) and DCIS_adjIDC (*n = *43). Rank sums test, **p < 0.01, ****p < 0.0001. K) The protein expression level of AKR1C1 in *AKR1C1*‐knockdown HCC1806 cells. Student's *t*‐test, **p < 0.01. L) The up‐regulated pathways in HCC1806 cells after knockdown *AKR1C1*. The *p* value was calculated by Fisher's exact test. M) DHE‐staining (left) and quantitative analysis (right) of the level of ROS in HCC1806 cells after knockdown *AKR1C1*. Scale bars = 50 µm. Student's *t*‐test, ***p < 0.001. N) ROS regulation of invasion and metastasis. ROS might induce DCIS_adjIDC cells invasion and migration via induction of transcription factor HIF‐1α, leaded to the upregulation of VEGF, and inhibition of tissue inhibitor of metalloproteinase 1 (TIMP1). O) Heatmap showing the expression levels of MMPs in DCIS_Pure (*n = *30) and DCIS_adjIDC (*n = *43). Rank sums test, *p < 0.05, **p < 0.01, ***p < 0.001, ****p < 0.0001. P) Representative IHC images of TIAM1, AR, and AKR1C1 on DCIS_Pure and DCIS_adjIDC tissues. Scale bars = 50 µm. Q) The diagram showing the TIAM1‐AR‐AKR1C1 alteration in DCIS_adjIDC and its effects on cell invasion and migration.

To further investigate how DCIS_adjIDC possessed the invasive ability and developed into IDC, we screened the differentially expressed molecules (FC (DCIS_adjIDC/DCIS_Pure) >2 or < 1/2, Wilcoxon rank‐sum test, *p* < 0.05) (**Figure** **5**A, Table S5, Supporting Information) between the DCIS_Pure and DCIS_adjIDC. Pathway enrichment analysis of differentially expressed molecules showed that DCIS_adjIDC‐enriched molecules were involved in steroid hormone biosynthesis, O‐glycan biosynthesis, RHO GTPase cycle, and HIF‐1 signaling pathway, whereas molecules enriched in DCIS_Pure mainly participated in the regulation of valine, leucine, and isoleucine degradation, pentose phosphate, complement and coagulation cascades, and focal adhesion (Figure 5A, Table S5, Supporting Information).

To determine the divergence of genomic drivers in DCIS_Pure and DCIS_adjIDC, we compared the differences in genomic variations between them. At the focal event level, chromosome gains, such as chromosome 21q22.11 and 4p16.1 (Fisher's exact test, *p* < 0.05) gains, were more predominant in DCIS_adjIDC (*n = *23) than in DCIS_Pure (*n = *9) (Fisher's exact test, *p* < 0.05) (Figure 5A). T‐cell lymphoma invasion and metastasis 1 (TIAM1) in 21q22.11, significantly positive *cis*‐effect, were upregulated in DCIS_adjIDC compared with DCIS_Pure (FC (DCIS_adjIDC/DCIS_Pure) > 2, Wilcoxon rank‐sum test, *p* < 0.05) (Figure 5A). Further, we found that the significantly positive *cis*‐effect of *TIAM1* was also observed on the mRNA and protein levels in the CPTAC BC cohort, respectively^[^ ^11^ ^]^ (Spearman's rho > 0.3, *p* < 0.05) (Figure S5A, Supporting Information). *TIAM1* encodes a large protein that functions as a GDP to GTP exchange factor for Rho GTPases or Rho‐like GTPases, such as RAC family small GTPase 1 (RAC1), RAC family small GTPase 2 (RAC2), RAC family small GTPase 3 (RAC3), RAS homolog family member A (RHOA), and cell division cycle 42 (CDC42).^[^ ^50^, ^51^ ^]^ Among them, the RAC family members were upregulated in DCIS_adjIDC, while RHOA and CDC42 were upregulated in DCIS_Pure (Figure 5B). The expression level of TIAM1 was significantly positive correlated with that of RAC3 (Spearman rho = 0.66, *p* = 6.51e‐12), but not with RAC1 (Spearman rho = 0.09, *p* = 8.02e‐2) and RAC2 (Spearman rho = 0, *p* = 9.98e‐1) (Figure 5B,C, and Figure S5B, Supporting Information). A significantly positive correlation between TIAM1 and RAC3 was also observed in the CPTAC BC cohort (Spearman's rho = 0.34, *p* = 1.70e‐3) (Figure S5C, Supporting Information). These results suggested that TIAM1 may act as a GDP to GTP exchange factor for RAC3 in DCIS_adjIDC.

Notably, the expression level of RAC3 was significantly positively correlated with the score of the steroid hormone biosynthesis pathway (Spearman rho = 0.62, *p* = 1.05e‐18) (Figure 5D), and the steroid hormone biosynthesis pathway was upregulated in DCIS_adjIDC (Figure S5D, Supporting Information). Correspondingly, it was found that only the protein expression level of AR among the 6 steroid hormone receptors was upregulated in DCIS_adjIDC compared with DCIS_Pure (Figure 5E), and the transcriptional activity of AR in DCIS_adjIDC was higher than in DCIS_Pure (Figure S5E, Supporting Information). In addition, LIM kinase1 (LIMK1) was reported as the characterized downstream effectors of Rho GTPases and involved in AR nuclear translocation.^[^ ^52^ ^]^ Further, we found that the correlation between the expressions of RAC3 and LIMK1 was 0.57 (Spearman *p* = 1.20e‐7), and the correlation between the expressions of LIMK1 and AR was 0.59 (Spearman *p* = 3.67e‐08) (Figure 5F and Figure S5F, Supporting Information). These results suggested that the activated RAC3 may promote the nuclear translocation of AR through LIMK1.

Further, we screened the target genes of AR and found that aldo‐keto reductase family 1 member C1 (AKR1C1) was significantly upregulated both on the mRNA and protein levels in DCIS_adjIDC (Figure 5G; Figure S5G and Table S5, Supporting Information). It has been reported that AKR1C1 was involved in the regulation of the reactive oxygen species (ROS) metabolic process.^[^ ^53^ ^]^ Subsequently, we found that the expression level of AKR1C1 was significantly negatively correlated with the score of the ROS pathway, which was decreased significantly in DCIS_adjIDC (Figure 5H and Figure S5H, Supporting Information), suggesting that AKR1C1 could remove excess ROS in DCIS_adjIDC cells (Figure 5H). The removal of excessive intracellular ROS prevented the apoptosis of tumor cells and promoted migration and invasion of tumor cells.^[^ ^54^, ^55^, ^56^, ^57^ ^]^ Surprisingly, on the one hand, the apoptosis pathway (e.g., MYC associated factor X (MAX), myocyte enhancer factor 2C (MEF2C), BCL2 like 11 (BCL2L11), BH3 interacting domain death agonist (BID), caspase 3 (CASP3), and apoptotic peptidase activating factor 1 (APAF1)) was downregulated in DCIS_adjIDC compared with DCIS_Pure (Figure 5I,J). Further, after the knockdown of *AKR1C1* in human breast cancer cells HCC1806, we found that the apoptosis signaling pathway was significantly upregulated (Figure 5K,L; Figure S5I and Table S5, Supporting Information) and the mean fluorescence intensity (MFI) of ROS was significantly increased (Figure 5M). On the other hand, this is consistent with previous reports that ROS might induce DCIS_adjIDC cells invasion and migration via induction of hypoxia‐inducible factor 1α (HIF‐1α), leading to the upregulation of vascular endothelial growth factor (VEGF), and inhibition of tissue inhibitor of metalloproteinase 1 (TIMP1), leading to the upregulation of matrix metallopeptidases (MMPs)^[^ ^54^, ^58^ ^]^ (Figure 5N,O). Further, IHC results showed that the expression of TIAM1, AR, and AKR1C1 in DCIS_adjIDC was higher than that in DCIS_Pure (Figure 5P and Figure S5J, Supporting Information). Based on the machine learning algorithm, the area under the curve (AUC) for distinguishing DCIS_adjIDC from DCIS_Pure was 0.96 using TIAM1, AR, and AKR1C1 expression features based on the machine learning algorithm (Figure S5K, Supporting Information). Overall, these results showed the TIAM1‐AR‐AKR1C1 axis promotes cell invasion and migration in DCIS_adjIDC (Figure 4Q).

### 2.5 AKR1C1 is a Potential Targetable Protein in BC

**Figure 6:**
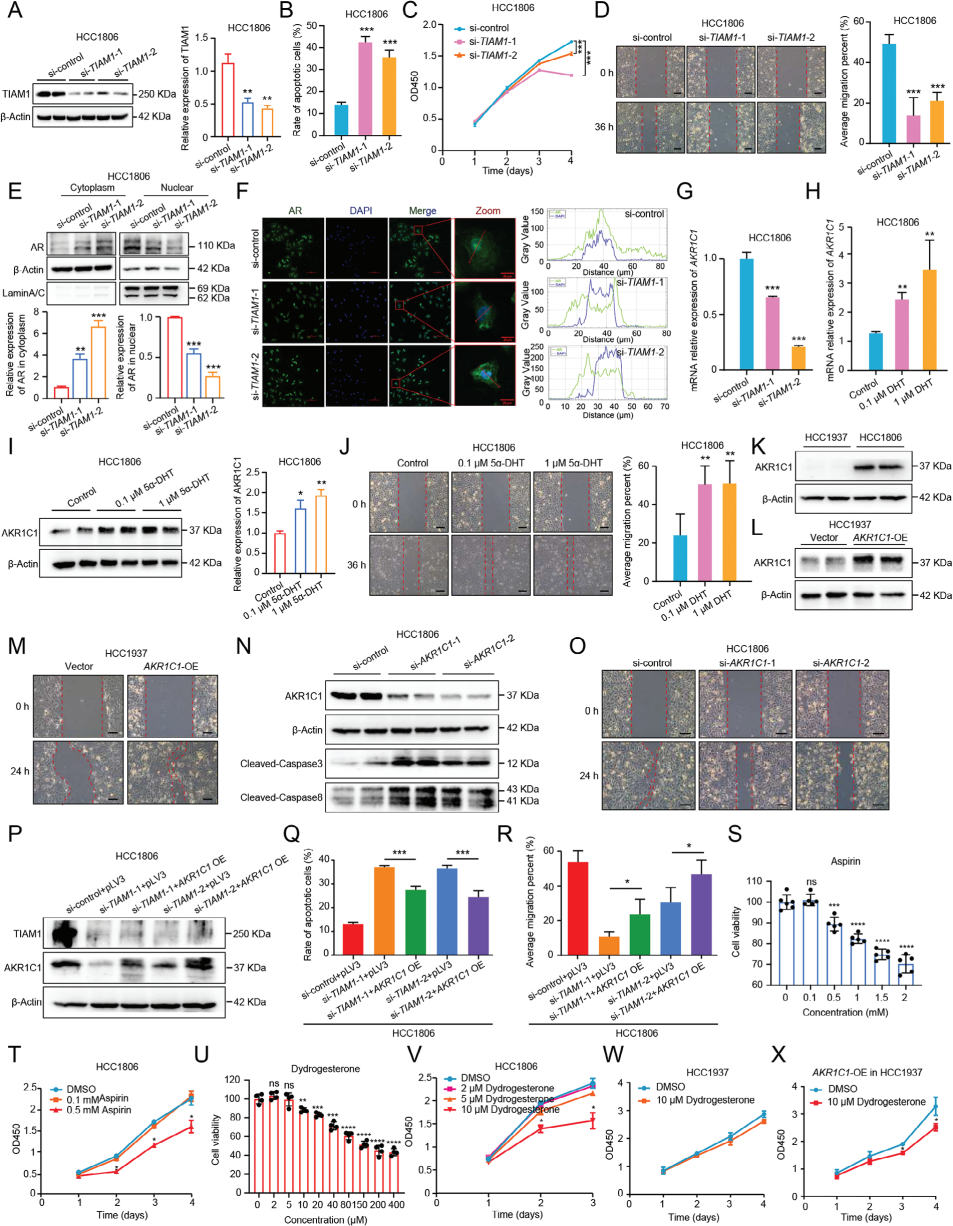
AKR1C1 is a Potential Targetable Protein in BC. A) A representative western blot analysis of TIAM1 in *TIAM1*‐knockdown HCC1806 cells (left). Quantified western blot results (right). Student's *t*‐test, **p < 0.01. B) Flow cytometry analysis with Annexin V‐PI staining was performed to evaluate the percentage of apoptotic cells in *TIAM1*‐knockdown HCC1806 cells. C) The optical density at 450 nm (OD450) every 24 h after *TIAM1* knockdown was recorded and the growth curves were drawn accordingly. D) Wound healing assays were performed on HCC1806 cells or that knocked down *TIAM1* (left). Scale bars = 100 µm. The average migration percent was calculated (right). Student's *t*‐test, ***p < 0.001. E) A representative western blot analysis of AR in cytoplasm and nuclear in *TIAM1*‐knockdown HCC1806 cells (upper). Quantified western blot results (bottom). Student's *t*‐test, ***p < 0.001. F) Representative image shows that *TIAM1* knockdown inhibited AR nuclear translocation in HCC1806 cells (left). Plots of pixel intensity along the red line from top, middle, and bottom rows of images to the left of each plot (right). Scale bars = 20 µm. G) Real‐time PCR analysis of *AKR1C1* in *TIAM1*‐knockdown HCC1806 cells. Student's *t*‐test, ***p < 0.001. H) Real‐time PCR analysis of *AKR1C1* in HCC1806 cells treatment with different doses of 5α‐DHT. Student's *t*‐test, **p < 0.01. I) A representative western blot analysis of AKR1C1 in HCC1806 cells treatment with different doses of 5α‐DHT (left). Quantified western blot results (right). Student's *t*‐test, *p < 0.05, **p < 0.01. J) Wound healing assays were performed on HCC1806 cells or that treatment with different doses of 5α‐DHT (left). Scale bars = 100 µm. The average migration percent was calculated (right). Student's *t*‐test, **p < 0.01. K) A representative western blot analysis of AKR1C1 in wild‐type HCC1937 and HCC1806 cells. L) A representative western blot analysis of AKR1C1 in *AKR1C1*‐ overexpressing HCC1937 cells. M) Wound healing assays were performed on HCC1937 cells or that overexpressing *AKR1C1*. Scale bars = 100 µm. N) A representative western blot analysis of cleaved‐Caspase 3 and cleaved‐Caspase 8 in *AKR1C1*‐knockdown HCC1806 cells. O) Wound healing assays were performed on HCC1806 cells or that knocked down *AKR1C1*. Scale bars = 100 µm. P) Control vector or AKR1C1 overexpression vector was expressed in *TIAM1*‐knockdown HCC1806 cells, and the AKR1C1 expression level was detected by western blotting. Q) Control vector or AKR1C1 overexpression vector was expressed in *TIAM1*‐knockdown HCC1806 cells, and flow cytometry analysis with Annexin V‐PI staining was performed to evaluate the percentage of apoptotic cells. Student's *t*‐test, ***p < 0.001. S) Aspirin was able to inhibit cell viability of HCC1806 in a dose‐dependent manner. Student's *t*‐test, ns, not significant, ***p < 0.001, ****p < 0.0001. T) HCC1806 cells were treated with DMSO or different concentrations of aspirin 0.1 mm and 0.5 mm). The optical density at 450 nm every 24 h after added inhibitor was recorded and the growth curves were drawn accordingly. Student's *t*‐test, *p < 0.05. U) Dydrogesterone was able to inhibit cell viability of HCC1806 in a dose‐dependent manner. Student's *t*‐test, ns, not significant, **p < 0.01, ***p < 0.001, ****p < 0.0001. V) HCC1806 cells were treated with DMSO or different concentrations of dydrogesterone (2 µm, 5 µm, and 10 µm). The optical density at 450 nm every 24 h after added inhibitor was recorded and the growth curves were drawn accordingly. Student's *t*‐test, *p < 0.05. W) HCC1937 cells were treated with DMSO or dydrogesterone (10 µm). The optical density at 450 nm every 24 h after added inhibitor was recorded and the growth curves were drawn accordingly. Student's *t*‐test, ns: not significant. X) HCC1937 cells overexpressing *AKR1C1* were treated with DMSO or dydrogesterone (10 µm). The optical density at 450 nm every 24 h after added inhibitor was recorded and the growth curves were drawn accordingly. Student's *t*‐test, *p < 0.05.

In order to systematically verify the function and mechanism of the TIAM1‐AR‐AKR1C1 axis in BC, we executed a series of experimental studies. Firstly, to verify the effect of TIAM1 on cell migration and apoptosis, we applied siRNA against *TIAM1* to knock down the expression of TIAM1 in HCC1806. The efficiency of *TIAM1* knockdown cells was verified through mRNA and protein expression levels (**Figures** **6**A and S6A, Supporting Information). Next, we performed cell apoptosis and migration assays of HCC1806 or that with *TIAM1* knockdown. To examine whether *TIAM1* knockdown affects cell apoptosis, flow cytometry analysis with Annexin V‐PI staining was performed to evaluate the percentage of apoptotic cells in *TIAM1*‐knockdown HCC1806 cells. We found that *TIAM1* knockdown significantly promoted apoptosis compared with HCC1806 treated with scrambled siRNA (Figure 6B and Figure S6B, Supporting Information). Additionally, we examined whether TIAM1 knockdown affects breast cancer cell HCC1806 growth by CCK‐8 assay. As shown in Figure 6C, the proliferation of HCC1806 cells was inhibited by *TIAM1* knockdown. Moreover, the migration capacity of HCC1806 or that with *TIAM1* knockdown was examined by wound healing assay. We found that *TIAM1* knockdown reduced the migration distance compared with HCC1806 treated with scrambled siRNA (Figure 6D). Taken together, we confirmed that TIAM1 promoted the migration of tumor cells and inhibited cell apoptosis.

Further, to study whether the effect of TIAM1 on migration and cell apoptosis is through AR nuclear translocation – AKR1C1 TF‐TG pair, we extracted the nucleoprotein of *TIAM1*‐knockdown HCC1806 cells, and detected the expression levels of nucleoprotein AR. We found that the expression level of AR in the nucleus was significantly decreased after *TIAM1* knockdown (Figure 6E). In addition, we performed immunofluorescence and observed AR was uniformly distributed in the nucleus and cytoplasm in wild‐type HCC1806 cells (Figure 6F). However, little colocalization between AR and nucleus in *TIAM1*‐knockdown HCC1806 cells (Figure 6F). Further, we found that the transcription level was significantly decreased in *TIAM1*‐knockdown HCC1086 cells compared with wild‐type HCC1806 cells (Figure 6G). These results suggested that *TIAM1* knockdown inhibited AR nuclear translocation, and reduced the transcription level of AKR1C1. Then, to verify the role of AR nuclear translocation in AKR1C1 expression and BC progression, we treated the HCC1806 cells with 5α‐dihydrotestosterone (5α‐DHT), an AR nuclear translocation activator.^[^ ^59^ ^]^ To demonstrate the effect of 5α‐DHT on AR nuclear translocation, we performed immunofluorescence and observed more colocalization between AR and nucleus in HCC1806 cells after treatment 5α‐DHT (0.1 µm and 1 µm) (Figure S6C, Supporting Information). These results suggested that 5α‐DHT promoted AR nuclear translocation in HCC1806 cells. Then, we performed a cell migration assay and detected the expression level of AKR1C1 after treatment 5α‐DHT. The results showed that both 0.1 µm and 1 µm 5α‐DHT could remarkably enhance the expression level of AKR1C1 on the mRNA and protein levels (Figure 6H,I). In addition, the wound healing assay further demonstrated that both 0.1 µm and 1 µm 5α‐DHT promoted the migration of HCC1806 cells (Figure 6J). Together, these results suggested that TIAM1 promoted AR nuclear translocation, which regulates the transcription of AKR1C1, and may result in cell migration.

Next, to confirm the role of AKR1C1 in BC, two human BC cell lines, HCC1806 and HCC1937 with differential AKR1C1 expressions were selected for further study (Figure 6K and FIgure S6D, Supporting Information). HCC1937, which showed lower expression of AKR1C1, was transfected with a lentiviral vector carrying the *AKR1C1* gene to harbor stable expression of AKR1C1 (Figure 6L and Figure S6E,F, Supporting Information) (Experimental Section). Notably, the migration ability of cells overexpressing AKR1C1 was elevated (Figure 6M and Figure S6G,H, Supporting Information). On the contrary, the siRNA against *AKR1C1* was applied to knock down the expression of AKR1C1 in HCC1806. The efficiency of *AKR1C1* knockdown was verified through protein expression (Figure 6N and Figure S6I, Supporting Information). To demonstrate the effect of AKR1C1 on cell apoptosis, we examined the expression of apoptosis‐related molecules by western blotting. Compared with cells treated with control siRNA, the expression levels of apoptosis‐related molecules caspase 3 and caspase 8 were significantly higher in *AKR1C1*‐knockdown HCC1806 cells (Figure 6N and Figure S6I, Supporting Information). The results suggested that AKR1C1 prevented cell apoptosis. Further, to demonstrate the effect of AKR1C1 on cell migration, the cell migration capacity of HCC1806 after AKR1C1 deficiency was examined by wound healing and transwell assay. We found that *AKR1C1* knockdown significantly reduced the migration distance (Figure 6O and Figure S6J, Supporting Information) and the number of migrated cells (Figure S6K, Supporting Information). These results suggested that AKR1C1 promoted cell migration. In addition, to examine whether TIAM1 accelerates migration and inhibits apoptosis of HCC1806 cells via AKR1C1, we performed rescue experiments by overexpressing AKR1C1 in *TIAM1*‐knockdown HCC1806 cells (Figure 6P and Figure S6L,M, Supporting Information). Notably, AKR1C1 overexpression in *TIAM1*‐knockdown HCC1806 cells effectively reversed the effects of *TIAM1* silencing on cell apoptosis and migration (Figure 6Q,R and Figure S6N,O, Supporting Information). These results suggested that TIAM1 facilitates AKR1C1 expression, thereby accelerating the migration and inhibiting the apoptosis of HCC1806 cells.

Moreover, based on the role of the TIAM1‐AR‐AKR1C1 axis in BC progression, we determined the potential therapeutic effect of targeting AKR1C1 for the treatment of BC in vitro. It was first discovered by Dhagat et al. that aspirin, as a well‐known salicylic acid‐based drug, can inhibit the activity of AKR1C1.^[^ ^60^ ^]^ To verify the potential therapeutic effect of aspirin targeting AKR1C1 for BC, the minimum effective dose of aspirin (0.5 mm) was used to treat HCC1806 cells (Figure 6S). Then, we examined the level of cell apoptosis and the cell migration capacity after aspirin treatment compared with cells without aspirin treatment. The results showed that 0.5 mm aspirin can repress cell growth, and reduce the migration distance of HCC1806 (Figure 6T and Figure S6P, Supporting Information). We further employed a more efficient inhibitor targeting AKR1C1 for BC, dydrogesterone,^[^ ^61^ ^]^ to treat HCC1806. The results showed that the minimum effective dose of dydrogesterone (10 µm) remarkably repressed cell growth and cell migration of HCC1806 (Figure 6U,V and Figure S6R,S, Supporting Information). In addition, 10 µm dydrogesterone showed an extensive repression effect on the cell growth of the *AKR1C1* overexpressed HCC1937 than the wild‐type HCC1937 (Figure 5W,X). In a word, these results revealed the role of AKR1C1 in promoting tumor cell migration and inhibiting apoptosis, which can be mitigated by inhibitors, and provide a promising therapeutic option for AKR1C1 inhibitor treatment in patients with AKR1C1 overexpression.

### 2.6 The Progression Routes of Transition from DCIS to Four Clinical Subtypes of IDC

**Figure 7:**
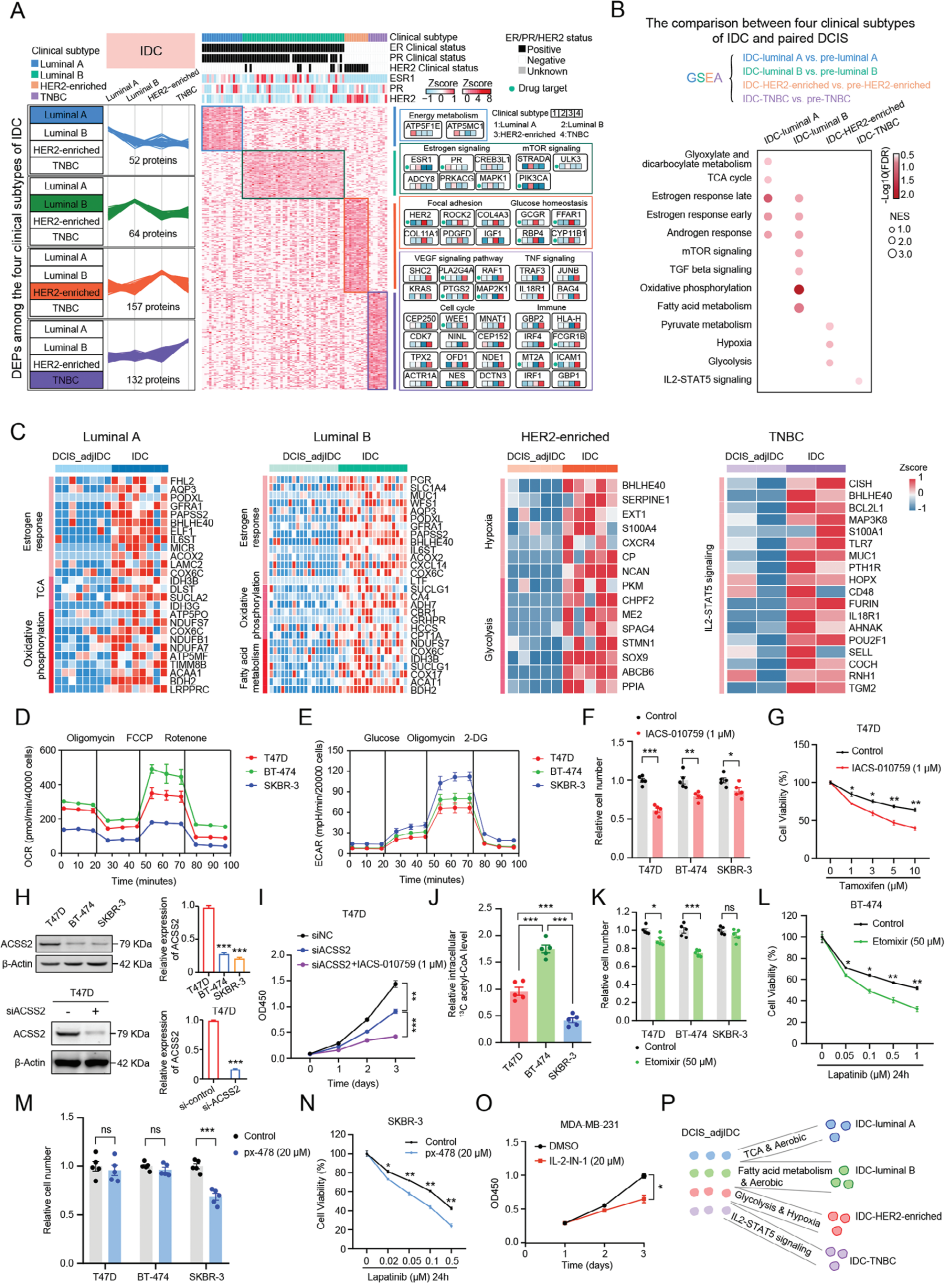
The Progression Routes of Transition from DCIS to Four Clinical Subtypes of IDC. A) Heatmap showing the proteome difference among the four clinical subtypes of IDC. The top panel shows the clinical annotation ER, PR, and HER2 status in clinical data and its related protein expression in proteome data. The bottom panel shows the differently expressed proteins (DEPs) among four clinical subtypes of IDC. The left panel shows 52, 64, 157, and 132 proteins that were significantly overrepresented in luminal A (*n = *17), luminal B (*n = *43), HER2‐enriched (*n = *10), and TNBC (*n = *8) samples, respectively (Fold change > 2, Student's t‐test, *p* < 0.05). The right panel shows the significantly enriched gene set in four clinical subtypes based on DEPs and the expression of the proteins involved in each gene set among four subtypes. B) GSEA was performed on four clinical subtypes of IDC and paired DCIS samples. The dot size represents the NES, and the dot color represents the significance. NES, normalized enrichment score. C) Heatmaps showing the up‐regulated pathways and proteins in four clinical subtypes of IDC compared to their paired DCIS_adjIDC, respectively (Fold change > 1.5 and Rank sums test, *p* < 0.05). D) Oxygen consumption rates (OCR) in T47D, BT‐474, and SKBR‐3 cells. E) Extracellular acidification rates (ECAR) in T47D, BT‐474, and SKBR‐3 cells. F) OXPHOS inhibitor IACS‐010759 (1 µm) significantly inhibited cell proliferation of T47D and BT474 cells, but not SKBR‐3 cells. Student's *t*‐test, *p < 0.05, **p < 0.01, ***p < 0.001. G) Combined use of tamoxifen and OXPHOS inhibitor IACS‐010759 (1 µm) inhibited T47D cell growth. Student's *t*‐test, *p < 0.05, **p < 0.01. H) Representative western blots analysis of ACSS2 in T47D, BT‐474, SKBR‐3 cells, and *ACSS2*‐knockdown T47D cells (upper left and bottom left). The quantitative data of the protein expression of ACSS2 in T47D, BT‐474, and SKBR‐3 cells (upper right). The quantitative data of the protein expression of ACSS2 in T47D cells expressing the control siRNA or *ACSS2* siRNA vector (bottom right). Student's *t*‐test, ***p < 0.001. I) Knockdown of ACSS2 using siRNA inhibited T47D cell growth, and usage of IACS‐010759 (1 µm) led to a more dramatic inhibition effect on siACSS2 cells. Student's *t*‐test, **p < 0.01, ***p < 0.001. J) Relative intracellular C13‐labelled acetyl‐CoA levels after C13‐labelled palmitic acid treatment in three cells. Student's *t*‐test, ***p < 0.001. K) Etomoxir (50 µm) significantly inhibited BT‐474 but not T47D and SKBR‐3 cell proliferation. Student's *t*‐test, ns, not significant, *p < 0.05, ***p < 0.001. L) Combined use of lapatinib and etomoxir (50 µm) inhibited BT‐474 cell growth. Student's *t*‐test, *p < 0.05, **p < 0.01. M) HIF‐1α inhibitor px‐478 (20 µm) inhibited cell proliferation of SKBR‐3 cells. Student's *t*‐test, ns, not significant, ***p < 0.001. N) Combined use of lapatinib and px‐478 (20 µm) inhibited SKBR‐3 cell growth. Student's *t*‐test, *p < 0.05, **p < 0.01. O) IL‐2 inhibitor IL‐2‐IN‐1 significantly inhibited cell growth of MDA‐MB‐231 cells. Student's *t*‐test, *p < 0.05. P) The diagram showing the progression routes of transition from DCIS to four clinical subtypes of IDC.

At present, the multi‐omics studies on breast cancer mainly focus on the IDC stage. Based on the expressions of ER, PR, HER2, and (proliferation marker protein KI67) KI67, IDC was classified into four major clinical subtypes: luminal A, luminal B, HER2‐enriched, and TNBC.^[^ ^6^ ^]^ To illustrate the proteomic characteristics among four clinical subtypes of IDC, we compared the differences among the four clinical subtypes of IDC in our data set. The expression levels of ESR1, PR, and HER2 in four clinical subtypes of IDC (*n = *78) (luminal A = 17, luminal B = 43, HER2‐enriched = 10, and TNBC = 8) in our proteome data and IHC results were consistent with previous reports, indicating the reliability of our mass spectrometry data and clinical information.^[^ ^62^, ^63^ ^]^ Next, we identified 52, 64, 157, and 132 proteins that were significantly overrepresented in luminal A, luminal B, HER2‐enriched, and TNBC samples, respectively (Fold change > 2, Student's *t*‐test, *p* < 0.05) (**Figure** **7**A). Clustering and cluster‐specific enrichment analyses of the enriched proteins using ConsensusPathDB and DAVID databases showed the distinctive biological processes and pathways represented in luminal A, luminal B, HER2‐enriched, and TNBC samples (Figure 7A). Specifically, IDC‐luminal A was characterized by energy metabolism (i.e., ATP synthase F1 subunit epsilon (ATP5F1E) and ATP synthase membrane subunit C locus 1 (ATP5MC1)). IDC‐Luminal B was characterized by estrogen signaling (i.e., ESR1 and PR) and mTOR signaling (i.e., STE20‐related adaptor alpha (STRADA) and UNC‐51 like kinase 3 (ULK3)). IDC‐HER2‐enriched was related to focal adhesion (i.e., HER2 and collagen type XI alpha 1 chain (COL11A1)) and glucose homeostasis (i.e., glucagon receptor (GCGR) and free fatty acid receptor 1 (FFAR1)). TNBC was characterized by cell cycle (i.e., cyclin dependent kinase 7 (CDK7), centrosomal protein 250 (CEP250), and WEE1 G2 checkpoint kinase (WEE1)), VEGF signaling pathway (i.e., SHC adaptor protein 2 (SHC2), phospholipase A2 group IVA (PLA2G4A), and RAF‐1 proto‐oncogene (RAF1)), TNF signaling (i.e., TNF receptor‐associated factor 3 (TRAF3), JUN proto‐oncogene (JUNB), and Interleukin 18 receptor 1 (IL18R1)), and immune (i.e., major histocompatibility complex, class I, H (HLA‐H), interferon regulatory factor 4 (IRF4), and Fc gamma receptor 1b (FCGR1B)). These results were consistent with previous studies, further illustrating the reliability of our mass spectrometry data and clinical information.

Due to there being no IDC‐paired DCIS samples in previous multi‐omics study cohorts, little is known about how the four clinical subtypes of patients progress from DCIS to IDC. Following pathology review, 33 IDC patients which synchronously diagnosed with DCIS and IDC components were included in this study. DCIS regions (DCIS_adjIDC, DCIS adjacent to IDC) and IDC regions on the same slide from the same patient were delineated by the pathologist (Figure S7A, Supporting Information). Further, under the guidance of a pathologist, we applied LCM to dissect the regions of IDC and paired DCIS precisely. The 33 IDC samples included 8 luminal A, 18 luminal B, 5 HER2‐enriched, and 2 TNBC samples. It is known that DCIS is recognized as a precursor of IDC.^[^ ^64^ ^]^ To study the progression routes of four subtypes of IDC, the DCIS_adjIDC samples were divided correspondingly into pre‐luminal A (*n = *8), pre‐luminal B (*n = *18), pre‐HER2‐enriched (*n = *5), and pre‐TNBC (*n = *2) (Figure S7B, Supporting Information).

To investigate progressive mutational changes and identify specific mutations that were discordant in DCIS_adjIDC and IDC, we compared the mutations of the DCIS_adjIDC samples with that of the paired IDC samples (*n = *28). The 28 paired samples included 14 DCIS_adjIDC samples (pre‐luminal A, *n = *4; pre‐luminal B, *n = *7; pre‐HER2‐enriched, *n = *2; and pre‐TNBC, *n = *1) and 14 IDC samples (luminal A, *n = *4; luminal B, *n = *7; HER2‐enriched, *n = *2; and TNBC, *n = *1). Our data showed that the total number of mutations was highly consistent between DCIS_adjIDC and IDC samples (Paired Student's *t*‐test, *p* = 0.15) (Figure S7C, Supporting Information). Consistent with Casasent's study,^[^ ^65^ ^]^ most mutations were highly consistent between the DCIS_adjIDC and their paired IDC, such as *TP53*, *PIK3CA*, *KMT2D*, *FAT3*, *ARID1A*, *GATA3*, *PREX2*, *APC*, *ERBB2*, suggesting that they were acquired in the DCIS_adjIDC prior to IDC (Figure S7D, Supporting Information).

Next, we performed PCA of 66 paired DCIS_adjIDC and IDC samples in our cohort. PCA of DCIS_adjIDC proteomes and IDC proteomes revealed a clear boundary among the four clinical subtypes respectively, indicating the proteomes of the four subtypes of IDC patients had been significantly different in DCIS_adjIDC (Figure S7E,F, Supporting Information). Further, we compared the differences among pre‐luminal A, pre‐luminal B, pre‐HER2‐enriched, and pre‐TNBC in our data set. We identified 99, 70, 173, and 354 proteins that were significantly overrepresented in pre‐luminal A, pre‐luminal B, pre‐HER2‐enriched, and pre‐TNBC samples, respectively (Fold change > 2, Student's *t*‐test, *p* < 0.05) (Figure S7G, Supporting Information). Clustering and cluster‐specific enrichment analyses of the enriched proteins using ConsensusPathDB and DAVID databases showed the distinctive biological processes and pathways represented in pre‐luminal A, pre‐luminal B, pre‐HER2‐enriched, and pre‐TNBC samples (Figure S7G, Supporting Information). Specifically, pre‐luminal A was characterized by lysine degradation (i.e., KMT2C, ACAT1, and EHMT1). pre‐luminal B was characterized by protein folding (i.e., GNA15, CSNK2A3, and ACTB) and mTOR signaling (i.e., WNT5B, STK11, and DEPDC5). pre‐HER2‐enriched was related to extracellular matrix organization (i.e., TGFB2, COL6A5, and ITGA2), adherence junction (i.e., CTNND1, RAC2, and SNAI2), and pentose phosphate pathway (i.e., TKT and PGLS). pre‐TNBC was characterized by G2/M transition (i.e., RBBP4, CEP152, and TUBGCP4), cellular responses to stress (i.e., H2AX, EIF2AK4, and RPL7A), BCR signaling pathway (i.e., PAG1, IBTK, and PIK3R1), and neutrophil degranulation (i.e., DNAJC3, TIMP2, and PA2G4). These results further suggested that the proteomes of the four subtypes of IDC patients had been significantly different in DCIS_adjIDC.

To elucidate how DCIS progressed into different subtypes of IDC, we conducted a pair‐wise comparison of four clinical subtypes of IDC and paired DCIS_adjIDC samples by gene set enrichment analysis (GSEA), respectively. DCIS_adjIDC samples were divided correspondingly into pre‐luminal A (*n = *8), pre‐luminal B (*n = *18), pre‐HER2‐enriched (*n = *5), and pre‐TNBC (*n = *2). Therefore, Figure 7B shows the up‐regulated pathways in the four clinical subtypes of IDC compared to the corresponding DCIS_adjIDC, respectively. GSEA showed that compared with pre‐luminal A, estrogen response (e.g., FHL2, AQP3, GFRA1, PODXL, etc.), oxidative phosphorylation (OXPHOS) (e.g., ATP5PO, NDUFS7, COX6C, NDUFB1, ACAA1, etc.) and TCA cycle (e.g., IDH3B, DLST, SUCLA2, and IDH3G) were upregulated in IDC‐luminal A (FC > 1.5, Wilcoxon rank‐sum test, *p* < 0.05). Compared with pre‐luminal B, estrogen response (e.g., PGR, SLC1A4, MUC1, WFS1, AQP3, etc.), OXPHOS (e.g., NDUFS7, COX6C, IDH3B, SUCLG1, ACAT1, etc.) and fatty acid metabolism (e.g., SUCLG1, CA4, ADH7, CBR1, GRHPR, etc.) were upregulated in IDC‐luminal B (FC > 1.5, Wilcoxon rank‐sum test, *p* < 0.05). Compared with pre‐HER2‐enriched, glycolysis (e.g., PKM, CHPF2, ME2, SPAG4, STMN1, etc.) and hypoxia (e.g., BHLHE40, SERPINE1, EXT1, SLC37A4, KIF5A, etc.) were upregulated in IDC‐HER2‐enriched (FC > 1.5, Wilcoxon rank‐sum test, *p* < 0.05). Compared with pre‐TNBC, IL2‐STAT5 signaling (e.g., BCL2L1, TLR7, MUC1, PTH1R, CD48, etc.) was upregulated in IDC‐TNBC (FC > 1.5, Wilcoxon rank‐sum test, *p* < 0.05) (Figure 7B,C; Figure S7H and Table S6, Supporting Information). These data indicate that, during the transformation from DCIS to IDC, IDC‐luminal A was defined as “aerobic and TCA type”, IDC‐luminal B was defined as “aerobic and fatty acid metabolism type”, IDC‐HER2‐enriched was defined as “hypoxia and glycolysis type”, and IDC‐TNBC was defined as “immune type”.

To verify the progression routes of transition from DCIS to clinical subtypes of IDC, four human breast cancer cell lines (including T47D (IDC‐luminal A, HR+/HER2‐), BT‐474 (IDC‐luminal B, HR+/HER2+), and SKBR‐3 (IDC‐HER2‐enriched, HR‐/HER2+), and MDA‐MB‐231 (IDC‐TNBC, HR‐/HER2‐)^[^ ^66^ ^]^) were selected to better mimic the pattern of IDC subtypes identified in our proteomic data. By analyzing the oxygen consumption rate (OCR) and extracellular acidification rate (ECAR), we confirmed that the two luminal subtype cell lines T47D and BT‐474 showed more aerobic activity than that of SKBR‐3, while SKBR‐3 showed more potent glycolytic activity (Figure 7D,E), supporting the notion that IDC‐luminal subtypes are “aerobic types” and IDC‐HER2‐enriched is “glycolytic type”.

Proteomic data showed that compared with pre‐luminal subtypes, the OXPHOS and estrogen response pathways were upregulated in IDC‐luminal subtypes (Figure 7C). To target the pathway of OXPHOS, IACS‐010759 was used to treat breast cancer cells as a clinical‐grade small‐molecule inhibitor of mitochondrial complex I of OXPHOS.^[^ ^67^ ^]^ The inhibitory effect of IACS‐010759 (1 µm) on T47D and BT‐474 cells was better than that on SKBR‐3 cells (Figure 7F). The combined usage of IACS‐010759 and tamoxifen, an estrogen receptor inhibitor, showed a better inhibitory effect on the cell viability of T47D cells than tamoxifen alone (Figure 7G). In addition, compared with pre‐luminal A, the pathway of TCA cycle was upregulated in IDC‐luminal A (Figure 7C). The previous study has shown that acyl‐CoA synthetase short chain family member 2 (ACSS2) in the TCA cycle pathway could contribute to cancer cell growth under low‐oxygen and lipid‐depleted conditions.^[^ ^68^ ^]^ We confirmed that ACSS2 was expressed highly in T47D cells (Figure 7H). Knockdown of ACSS2 using siRNA inhibited T47D cell growth and usage of IACS‐010759 (1 µm) led to a more dramatic inhibition effect on siACSS2 cells (Figure 7I).

Proteomic data showed that compared with pre‐luminal B, fatty acid oxidation was relatively vigorous in IDC‐luminal B (Figure 7C). As expected, C13‐labelled acetyl‐CoA formation was highest in BT‐474 cells cultured in a medium with C13‐labelled palmitic acid (Figure 7J), suggesting that fatty acid oxidation is more active in BT‐474 cells. To target the pathway of fatty acid oxidation, etomixir (50 µm) was used to treat breast cancer cells as a carnitine palmitoyltransferase‐1 (CPT‐1, rate‐limiting enzyme for fatty acid oxidation) inhibitor, which inhibited the growth of BT‐474 cells but not SKBR‐3 cells (Figure 7K). Lapatinib as a small molecule inhibitor of HER2, has been developed to expand the options for treating HER‐positive breast cancer.^[^ ^69^ ^]^ Further, we found that the combined usage of etomoxir (50 µm) and lapatinib significantly decreased the cell viability of BT‐474 cells than that using lapatinib alone (Figure 7L).

Proteomic data showed that compared with pre‐HER2‐enriched, the hypoxia pathway was upregulated in IDC‐HER2‐enriched (Figure 7C). To target the pathway of hypoxia, the HIF‐1α inhibitor px‐478 (20 µm) was used to treat breast cancer cells, which inhibited the cell number of SKBR‐3 cells but not luminal subtype cell lines (Figure 7M). The combined usage of px‐478 (20 µm) and lapatinib inhibited SKBR‐3 cells better than using lapatinib alone (Figure 7N).

Proteomic data showed that compared with pre‐TNBC, IL2‐STAT5 signaling was upregulated in IDC‐TNBC. To investigate the potential of targeting the IL‐2‐STAT5 pathway for TNBC breast cancer cells, we treated MDA‐MB‐231 cells with IL‐2‐IN‐1, an IL‐2 inhibitor.^[^ ^70^ ^]^ We found that the growth of MDA‐MB‐231 cells treated with 20 µm IL‐2‐IN‐1 was significantly inhibited (Figure 7O). These results suggested that IL‐2‐IN‐1 has the potential to be a therapeutic agent that can inhibit TNBC breast cancer cells.

In conclusion, we found that the inhibitory effect of IACS‐010759 on T47D cells was better than that on BT‐474 cells and SKBR‐3 cells. IACS‐010759 as a potential therapeutic agent for luminal A breast cancer cells, with its combination with tamoxifen showed a better inhibitory effect on these cells. Additionally, the inhibitory effect of etomixir on BT‐474 cells was better than that on T47D cells and SKBR‐3 cells. Etomixir demonstrated potential as a therapeutic agent for inhibiting luminal B breast cancer cells, with its combination with lapatinib resulting in a better inhibitory effect. Furthermore, px‐478 showed promise as a therapeutic agent for inhibiting HER2‐enriched breast cancer cells, with its combination with lapatinib proving to be more effective in this regard. Lastly, IL‐2‐IN‐1 had the potential to inhibit TNBC cells (Figure 7P).

## 3 Discussion

This study represents the very first attempt to perform a large‐scale proteogenomics‐integrative analysis of the characteristics of linear multi‐step progression of BRDC to elucidate progression mechanisms and discover stage‐special targeted therapies. In our cohort, multi‐omics data were generated as a public resource from a retrospective cohort of 224 FFPE samples. 224 FFPE samples in our cohort, spanned from 2007 to 2018, were collected and processed with the same standard operating procedure. To assess the stability of the samples in our cohort, we first analyzed the identification numbers of the samples that span from 2007 to 2018. As a result, we found that there was no significant difference in the protein identification number of the samples with different storage years at the same pathological stage (Figure S8A, Supporting Information). Then, we analyzed the expression of housekeeping genes in our cohort and found that the expression of the housekeeping genes (e.g., NDUFA13, TADA1, SKIV2L, etc.) was constant at the protein level in 224 samples spanning from 2007 to 2018 (Figure S8B, Supporting Information). These results implied that no significant impact of the time spanning from 2007 to 2018 on the protein identifications in this study.

BC is a hormone‐dependent tumor.^[^ ^71^ ^]^ Previous studies have reported that steroid hormones and their receptors play important roles in the development and progression of BC.^[^ ^72^ ^]^ Hanamura et al. reported that estrogen plays a crucial role in the progression of BC through the activation of estrogen receptor ESR1.^[^ ^73^ ^]^ Li et al. reported that PR is a biomarker used routinely at diagnosis to characterize breast cancer, which plays an important role in breast carcinogenesis and advancement.^[^ ^72^ ^]^ Omoto et al. reported that androgen and androgen receptors are involved in BC development and growth.^[^ ^74^ ^]^ These results created a natural paradox and gave rise to some thinking. Our study found that all of these studies may be correct and that these hormones are switched to follow a delicate temporal and spatial sequence during BRDC progression. To be specific, in the early stages of BRDC, the integrative multi‐omics analysis revealed that sterol hormone receptors (ESR1, NR3C1, and AR) were involved in the BRDC progression from DH to DCIS step by step (Figure S8C, Supporting Information). *TP53* mutation‐associated ESR1 overexpression was involved in the transition from DH to DCIS, 6q21 amplification‐associated NR3C1 overexpression helped DCIS_Pure (pure DCIS, no histologic evidence of invasion) cells avoid immune destruction, the TIAM1‐AR‐AKR1C1 axis promoted cell invasion and migration in DCIS_adjIDC (DCIS regions of invasive cancers) (Figure S8C, Supporting Information).

In this study, we found that *TP53* mutation occurred as early as in the tumorigenesis stage, with a frequency of 50% in DCIS and 34% in IDC. The proportion of *TP53* mutations decreases as DCIS progresses to more invasive stages, possibly due to reverse clonal selection. Reverse clonal selection — where the allelic frequency of aberrant genes decreases over the course of malignant progression — appears to occur in some cases.^[^ ^75^ ^]^ A striking example of this is noted with *BRAF* ^V600E^ mutations. *BRAF* ^V600E^ mutations are found in about 50% of melanomas, while they are found in about 80% of benign nevi.^[^ ^76^ ^]^ Similarly, whereas *ERBB2* overexpression in breast cancer is found throughout the benign‐to‐malignant transition, it is detected more frequently in DCIS (∼27‐56%) than in IDC (∼11‐20%).^[^ ^77^, ^78^, ^79^ ^]^ In bladder cancer, the frequency of *FGFR3* mutations is inversely correlated with the aggressiveness of the tumour, as grade 1 bladder cancer presents with the highest frequency of *FGFR3* mutations (∼60%), whereas the most aggressive, high‐grade tumours harbour *FGFR3* mutations in only ∼11% of cases.^[^ ^80^, ^81^, ^82^ ^]^ These observed phenomena may be due to the presence of clone subpopulations carrying distinct mutations or amplifications in the same patient, which have different advantages during tumor progression.

Chromosome amplification is usually one of the markers of tumorigenesis. Multiple tumor driver genes on the same chromosome segment can often synergistically promote tumor development. We found *FOXO3* in 6q21 showed *cis*‐effect and was reported participating in immune suppression^[^ ^83^ ^]^ (Figure S8D, Supporting Information). To determine the function of FOXO3, we further screened the TG of FOXO3. We observed an increased level of TG of FOXO3 TNFSF10 in DCIS_Pure, which exhibited a significant negative correlation with the immune score (Spearman's rho = −0.41, *p* = 1.17e‐4) (Figure S8D,E, Supporting Information). TNFSF10 has been reported to play a role in regulating immune microenvironments.^[^ ^84^ ^]^ In our cohort, we found a significantly negative correlation between TNFSF10 and CD8+ T cells using correlation analysis (Figure S8E, Supporting Information) (Spearman's rho = −0.42, *p* = 1.07e‐4). These results suggested that in addition to the dominant role of PRDM1 in immune suppression, *FOXO3* in 6q21 may have a synergistic effect. In addition, we found that SOD1 in 21q22.11 was amplified, leading to the protein (superoxide dismutase 1, SOD1) encoded by SOD1 was upregulated in DCIS_adjIDC compared with DCIS_Pure (Figure 5A) (Student's *t*‐test, *p* < 0.05). SOD1 is an enzyme that catalyzes the removal of ROS.^[^ ^85^ ^]^ In our cohort, we also observed a significantly negative correlation between the protein level of SOD1 and the score of the ROS pathway (Spearman's rho = −0.26, *p* = 0.028) (Figure S8F, Supporting Information). These results suggested that in addition to the dominant role of TIAM1‐AR‐AKR1C1 in the removal of excess ROS, *SOD1* in 21q22.11 may have a synergistic effect.

We advocate the importance of characterizing biological themes that cross histological boundaries and subdivide individual tumors of the same histology because such insights can lead to new extensions of treatments shown to be effective in one type of tumor to another, histologically same tumors possess disparate proteomics features. For example, DCIS represents a heterogeneous group that differs in its biological behavior and risk of progression, only a small percentage (14%−53%) of cases progress to IDC, resulting in a major clinical challenge in determining which patients to treat.^[^ ^12^ ^]^ In this study, we found that, although the DCIS_Pure and DCIS_adjIDC subgroups were not significantly different at the histological grade, PCA of our transcriptome, proteome, and phosphoproteome data revealed they were two distinct subgroups of DCIS. xCell^[^ ^86^ ^]^ is a web tool that performs cell type enrichment analysis from gene expression data for 64 immune and stroma cell types. It has been applied in many researche projects related to tumor proteomics and transcriptomics and has uncovered novel insights that have been validated by other independent experiments.^[^ ^11^, ^26^, ^87^, ^88^, ^89^ ^]^ Previous research has reported that the tumor microenvironment infiltration estimated by proteomic data had a high Pearson correlation with ones estimated by transcriptomic data^[^ ^26^, ^88^ ^]^ indicates the potential of proteome in xCell analysis to reveal tumor microenvironment infiltration. Therefore, in this study, to clarify the heterogeneity of DCIS, we attempted to evaluate the immune microenvironment by xCell based on proteomic data. xCell analysis showed that the abundance of CD8+ Tem, CD8+ Tcm, and CD8+ T cells in DCIS_adjIDC was higher than that in DCIS_Pure. Consistent with our conclusions, Alcazar et al. found that DCIS adjacent to IDC (DCIS_adjIDC) contained a significantly higher frequency of T cells than pure DCIS, and assessing the frequencies of activated CD8+ T cells in DCIS may identify patients with a higher risk of invasive progression.^[^ ^90^ ^]^ In addition, needle biopsy is a common method for the preoperative diagnosis of breast cancer.^[^ ^91^, ^92^ ^]^ However, due to the limited tissue sample size obtained by needle biopsy, it is possible to misdiagnose DCIS with IDC as DCIS.^[^ ^93^ ^]^ In this study, the AUC for distinguishing DCIS_adjIDC from DCIS_Pure was 0.96 using three protein expression features of TIAM1, AR, and AKR1C1 based on the machine learning algorithm (Figure S5M, Supporting Information), indicating that we could predict whether DCIS samples are accompanied by IDC to assist clinical diagnosis in the future.

AKR1C1 is a member of the AKR1C family and was reported to be highly expressed in different cancer types such as breast cancer, gastric cancer, bladder cancer, and non‐SCLC.^[^ ^94^, ^95^, ^96^, ^97^ ^]^ The overexpression of AKR1C1 often plays an essential role in cancer invasion, metastasis, and chemoresistance.^[^ ^96^ ^]^ Here, we found that AKR1C1 could remove excess ROS in DCIS_adjIDC cells, leading to preventing the apoptosis of tumor cells and promoting migration of tumor cells. Further, two kinds of reported inhibitors of AKR1C1, aspirin and dydrogesterone were used in BC cells, and can inhibit cell growth and migration. Currently, several clinical trials on observational studies have shown that taking aspirin improves survival in a small percentage of breast cancer patients.^[^ ^98^ ^]^ In addition, there are several ongoing clinical trials on observational studies of the association of dydrogesterone intake with breast cancer risk. Our observation suggests that aspirin and dydrogesterone can target AKR1C1 in BC, and DCIS_adjIDC patients with the TIAM1 amplification‐AR overexpression‐AKR1C1 overexpression axis may be able to receive aspirin or dydrogesterone adjuvant therapy.

Targeted therapies, including endocrine therapy and human epidermal growth factor receptor‐2 targeted therapy, marked a new era of breast cancer treatment.^[^ ^15^ ^]^ However, a certain proportion of breast cancer patients present with resistance to drug therapy, making it much more difficult to control the deterioration of the disease. Altered energy metabolism has become one of the hallmarks of cancer, including breast cancer.^[^ ^15^ ^]^ Adjuvant therapies targeting cellular metabolism may be a promising strategy to overcome drug resistance in cancer therapy. In 2018, Molina et al. reported the discovery of IACS‐010759 as a clinical‐grade small‐molecule inhibitor of mitochondrial complex I of OXPHOS.^[^ ^67^ ^]^ Further research has shown that treatment with IACS‐010759 robustly inhibited proliferation and induced apoptosis in models of brain cancer and acute myeloid leukemia (AML) reliant on the OXPHOS, likely owing to a combination of energy depletion and reduced aspartate production that leads to impaired nucleotide biosynthesis. In models of brain cancer and AML, tumor growth was potently inhibited in vivo following IACS‐010759 treatment at well‐tolerated doses. IACS‐010759 is currently being evaluated in phase 1 clinical trials in relapsed/refractory AML and solid tumors. In this study, during the transition from DCIS to IDC, we found that the progression routes of the three subtypes of IDC (luminal A, luminal B, and HER2‐enriched) are related to metabolism, but they are different. Based on the progression routes of the three subtypes of IDC, we found that the combination of the OXPHOS inhibitor IACS‐010759 and tamoxifen inhibited luminal A cells better than using tamoxifen alone. In addition, lapatinib as a small molecule inhibitor of HER2, has been developed to expand the options for treating HER‐positive breast cancer.^[^ ^69^ ^]^ We found that the combination of carnitine palmitoyltransferase‐1 (CPT‐1) inhibitor etomoxir and lapatinib inhibited luminal B cells better than using lapatinib alone. The HIF‐1α inhibitor px‐478 in combination with lapatinib inhibited HER2‐enriched cells better than using lapatinib alone. Altogether, we proposed adjuvant therapy for IDC targeting cell metabolism, which may provide a new strategy for overcoming drug resistance in IDC treatment.

In summary, our integrative proteogenomic analysis enables an understanding of the sequence of a series of biological events in the progression of BRDC and provides opportunities for the treatment of BRDC in different stages.

## 4 Experimental Section

### Patient Samples of the BRDC Progression Cohort—Construction of the BRDC Progression Cohort

In this study, two hundred and twenty‐four archival formalin‐fixed paraffin‐embedded (FFPE) tissues at 4 BRDC progression stages, including normal ductal epithelial tissue (Normal), ductal hyperplasia (DH, including usual ductal hyperplasia (UDH) and atypical ductal hyperplasia (ADH)), ductal carcinoma in situ (DCIS), and invasive ductal carcinoma (IDC), were randomly collected from 168 female patients with breast cancer and mammary benign disease. Breasts are composed of lobules, ducts, and adipose and fibroglandular tissues.^[^ ^16^ ^]^ Histopathologically, the progression of human BRDC was a linear multi‐step process that initiates as normal ductal epithelial tissue hyperplasia, progresses to DCIS, evolves into IDC, and culminates in the potentially lethal stage of lymph node metastasis and distant metastasis. In a word, breast NATs were normal ductal epithelial tissue in the study of BRDC progression. In our study, breast NATs were normal ductal epithelial tissue from patients with mammary benign disease.

### Patient Samples of the BRDC Progression Cohort—Sample Collection

The BRDC progression samples used for this study were collected from Zhongshan Hospital affiliated to Fudan University. Two hundred and twenty‐four archival formalin‐fixed paraffin‐embedded (FFPE) tissues at 4 BRDC progression stages were randomly collected from 168 female patients with BC and mammary benign disease from May 2007 to December 2018. Clinical information of 168 female patients including age, histological stage, degree of differentiation, TNM stage (AJCC cancer staging system 8^th^ edition), menopausal status, clinical subtype, and status of survival was summarized in Figure 1B and Table S1, Supporting Information. All patient samples were obtained with the hospital's approval of the Research Ethics Committee with written informed consent provided by all participants.

### Patient Samples of the BRDC Progression Cohort—Sample Selection Principles for Multi‐Omics Studies

The selection of samples for proteomic, phosphoproteomic, genomic, and transcriptomic studies needed to satisfy the following three principles: firstly, all the samples needed to be conducted on proteomic profiling. Secondly, after ensuring proteomic profiling, the samples underwent phosphoproteomic profiling and whole‐exome sequencing as much as possible. Finally, if there were any remaining samples, transcriptomic sequencing was conducted. Therefore, a proteomic, phosphoproteomic, genomic, and transcriptomic analysis was performed to profile the proteogenomic patterns of samples dissected from BRDC of different stages and grades in 168 female patients. Although the amount of tissue samples for studying BRDC progression was tiny, 224 samples for proteomic profiling, 49 samples for phosphoproteomic profiling, 79 samples for whole‐exome sequencing, and 42 samples for transcriptomic sequencing were still obtained. The samples covered the different stages and grades of BRDC progression, including the precancerous stages (Normal and DH) and the tumor stages (DCIS and IDC).

### Patient Samples of the BRDC Progression Cohort—Sample Preparation

The tissue specimens used were FFPE. The sample preparation followed FFomic strategy.^[^ ^88^ ^]^ BRDC tissues and Normal tissues were collected within 30 min after resection, immediately transferred into sterile freezing vials and snap frozen in liquid nitrogen, and then split and stored at ‐80 °C^[^ ^99^ ^]^ until being used. The cold ischemia time of this study was consistent with other large‐scale proteogenomic studies.^[^ ^26^, ^42^ ^]^ Accurate evaluation of tumor cellularity was determined using the middle section of each tumor tissue block, which was resected and subjected to hematoxylin and eosin (H&E) staining. For proteomic, genomic, and phosphoproteomic sample preparation, slides (10 µm thick) were sectioned, deparaffinized with xylene, and washed in an ethanol gradient. Specimens selected according to H&E staining were scraped using a dissecting microscope and then stored at −80 °C until needed. For RNA sample preparation, slides (10 µm thick) were sectioned, were not dewaxed, and stored at room temperature for further progression. Each sample was assigned a new research ID, and the patient's name or medical record number used during hospitalization was de‐identified.

### Patient Samples of the BRDC Progression Cohort—Histopathology Review

Hematoxylin and eosin (H&E) stained slides were reviewed and evaluated independently by three expert pathologists and information regarding tumor histological stage, degree of differentiation, TNM stage, and tumor purity were provided. Tumor sections were required to contain an average of 70% tumor cell nuclei with equal to or less than 20% necrosis for inclusion in the study. As for the normal breast tissue, the proportion of normal cells is 100%, while the tumor cell is 0% in the study. Additionally, all tumor samples were assessed for tumor content and the presence and extent of tumor necrosis. Tumor samples were also evaluated for the presence and extent of inflammatory infiltrates, as well as for the type of infiltrating cells (lymphocytes, neutrophils, eosinophils, histiocytes, plasma cells) in the tumor microenvironment. Any non‐concordant diagnoses among the three pathologists were re‐reviewed, and a resolution was reached following discussion.

### Patient Samples of the BRDC Progression Cohort—Laser‐Capture Microdissection (LCM)

In our cohort, H&E‐stained slides of all the samples of BRDC progression were reviewed and evaluated independently by three expert pathologists, and the areas of all the samples of BRDC progression were delineated. Further, under the guidance of a pathologist, LCM was applied to dissect the sections of samples precisely, which is generally used to improve the purity.^[^ ^18^ ^]^ All FFPE specimens were deparaffinized with xylene and rehydrated through graded alcohols and water. The H&E sections were stained with Mayer's haematoxylin and dehydrated through graded alcohols and xylene. Before microdissection, FFPE specimens were sectioned with a microtome (10 µm thick) and mounted from FFPE blocks were micro‐dissected with a Leica LMD 6500 laser microdissection system. All samples were systematically evaluated to confirm the histopathologic diagnosis and variant histology by three expert pathologists, and then an area of 1–5 × 10^6^ µm^2^ was collected (derived from dissected area × slide thickness/average mammalian cell volume of 2000 µm^3^, BNID 100 434). The samples were collected in 1.5‐mL tubes and kept in storage at −80 °C until further processing. The methods are also applied in other published proteogenomic studies, such as ovarian cancer^[^ ^100^ ^]^ and glioblastoma.^[^ ^101^ ^]^

### Proteomic and Phosphoproteomic Analysis—Protein Extraction and Tryptic Digestion

Samples were lysed in 100 µL TCEP buffer (2% deoxycholic acid sodium salt, 40 mm 2‐chloroacetamide, 100 mm Tris‐HCl, 10 mm Tris (2‐chloroethyl) phosphate, 1 mm PFSM, pH 8.5) supplemented with protease inhibitors and phosphatase at 99 °C for 30 min. After cooling to room temperature, trypsin (Promega, Madison, WI, USA, #V5280) was added and digested for 18 h at 37 °C. 10% formic acid was added and vortexed for 3 min, followed by sedimentation for 5 min (12,000 g). Next, a new 1.5‐mL tube with extraction buffer (0.1% formic acid in 50% acetonitrile) was used to extract the supernatant (vortex for 3 min, followed by 12,000 g of sedimentation for 5 min). Collected supernatant was transferred into a new tube for drying using a SpeedVac. After drying, 100 µL 0.1% FA was needed for dissolving the peptides and vortex for 3 min, and then sedimentation for 3 min (12,000 g). The supernatant was picked into a new tube and then desalinated. Before desalination, the activation of pillars with 2 slides of 3 M C8 disk is required, and the liquid is as follows: 90 µL 100% ACN twice, 90 µL 50% and 80% ACN once in turn, and then 90 µL 50% ACN once. After pillar balance with 90 µL 0.1% FA twice, the supernatant of the tubes was loading into the pillar twice, and decontamination with 90 µL 0.1% FA twice. Lastly, 90 µL elution buffer (0.1% FA in 50% ACN) was added into the pillar for elution twice and only the eluent was collected for MS. And then the collection liquid was put in a 60 °C vacuum drier for drying (∼1.5 h).

### Proteomic and Phosphoproteomic Analysis—The Enrichment of Phosphorylated Peptides

The peptide concentration was determined using a NanoDrop 2000C spectrophotometer (at 280 nm). The phosphoproteome samples were prepared by High‐Select Fe‐NTA Phosphopeptide Enrichment Kit (Thermo Fisher Scientific, A32992), following the manufacturer's recommendations. Briefly, 0.2 mg peptides were resuspended in 200 µL binding/wash buffer and loaded to the equilibrated spin column. The resin was mixed with the sample by gently tapping. The mixture was incubated for 30 min and centrifuged at 1000 × g for 30 s to discard the flowthrough. The column was then washed by 200 µL of binding/wash buffer and centrifuged at 1000 × g for 30 s for 3 times and washed by 200 µL of LC‐MS grade water for one additional time. The phosphopeptide was eluted by adding 100 µL of elution buffer and centrifuged at 1000 × g for 30 s for 2 times, and immediately dried using a SpeedVac (Eppendorf) at 45 °C for mass spectrometry analysis.

### Proteomic and Phosphoproteomic Analysis—Nano‐LC‐MS/MS

For the proteome profiling samples, peptides were analyzed on a timsTOF Pro ultra‐high resolution quadrupole time of flight mass spectrometer (Bruker Daltonics) coupled with a nanoElute UHPLC system (Bruker Daltonics). Dried peptides were re‐dissolved in 0.1% formic acid and injected onto a reverse phase C18 homemade 150 µm × 30 cm silica microcolumn (particle size, 1.9 µm; pore size, 120 Å; SunChrom, USA) using a nanoElute (Bruker Daltonics). Target on‐column load was 200 ng total peptide per injection with a pressure of 280 bar. The flow rate was 600 nL mi^−1^n. Mobile phase A was 0.1% formic acid in water; mobile phase B was 0.1% formic acid in acetonitrile. The gradient was linear from 2% B to 35% B over 110 min. The mass spectrometer was a timsTOF Pro (Bruker Daltonics) set to acquire data in Parallel Accumulation Serial Fragmentation (PASEF) mode. The TIMS accumulation time was set to 100 ms and precursor masses for 0.4 min where charge states of 2–4 were allowed.

For the phosphoproteomic samples, peptides were analyzed on a Orbitrap Fusion Lumos Hybrid Quadrupole‐Orbitrap Mass Spectrometer (Thermo Fisher Scientific) coupled with a high‐performance liquid chromatography system (EASY nLC 1200, Thermo Fisher Scientific). Dried peptide samples re‐dissolved in Solvent A (0.1% formic acid in water) were loaded onto a 2‐cm self‐packed trap column (100 µm inner diameter, 3 µm ReproSil‐Pur C18‐AQ beads, Dr Maisch GmbH) using Solvent A and separated on a 150 µm‐inner‐diameter column with a length of 30 cm (1.9 µm ReproSil‐Pur C18‐AQ beads, Dr Maisch GmbH) over a 150 min gradient (Solvent A: 0.1% Formic acid in water; Solvent B: 0.1% Formic acid in 80% ACN) at a constant flow rate of 600 nL mi^−1^n (0‐150 min, 0 min, 4% B; 0–10 min, 4%−15% B; 10–125 min, 15%−30% B; 125–140 min, 30%−50% B; 140–141 min, 50%−100% B; 141–150 min, 100% B). Eluted peptides were ionized at 2 kV and introduced into the mass spectrometer. Mass spectrometry was performed in data‐dependent acquisition mode. For the MS1 Spectra full scan, ions with *m/z* ranging from 300 to 1400 were acquired by an Orbitrap mass analyzer at a high resolution of 120000. The automatic gain control (AGC) target value was set to 3E+06. The maximal ion injection time was 80 ms. MS2 spectral acquisition was performed in the ion trap in a rapid speed mode with 1.5 s cycletime. Precursor ions were selected and fragmented with higher energy collision dissociation (HCD) with a normalized collision energy of 30%. Fragment ions were analyzed by an ion trap mass analyzer with an AGC target at 5E+04. The maximal ion injection time of MS2 was 20 ms. Peptides that triggered MS/MS scans were dynamically excluded from further MS/MS scans for 18 s. The coefficient of variation (CV) values on FAIMS were ‐45 V and ‐65 V.

### Proteomic and Phosphoproteomic Analysis—MS Database Searching

MS raw files generated by LC‐MS/MS were searched against the UniProt human proteome database using PEAKS Online software (Bioinformatics Solution Inc., Waterloo, Canada). Protease was Trypsin. Up to 2 missed cleavages were allowed. Carbamidomethyl (C) was considered as a fixed modification. For the proteome profiling data, variable modifications were oxidation (M) and acetylation (Protein N‐term). The cutoff of false discovery rate (FDR) by using a target‐decoy strategy was 1% for both proteins and peptides. For the phosphoproteomic data, variable modifications were oxidation (M), acetylation (Protein N‐term) and phospho (S/T/Y), and MS raw files were processed with Proteome Discoverer (ver. 2.3).

### Proteomic and Phosphoproteomic Analysis—Proteome Data Preprocess

For quality control of performance of MS, the HEK293T cell (National Infrastructure Cell Line Resource) was used as the quality control standard to monitor the MS stability. The quality control standard HEK293T cell was digested using the same method and conditions as the BRDC progression samples, and measured every three days. A Spearman's correlation coefficient was calculated for all quality control runs of HEK293T samples in a statistical analysis environment R (version 4.0.2). For quality control of proteome and phosphoproteome, the average correlation coefficient among the standards was 0.92 and 0.91, respectively, demonstrating the consistent stability of the MS platform.

To evaluate the reliability of the results of the cohort samples, all 224 BRDC progression samples were mixed as a BRDC samples pool. The quality control standard was measured every three days. A Spearman's correlation coefficient was calculated for all quality control runs in a statistical analysis environment R (version 4.0.2). For quality control of proteome and phosphoproteome, the average correlation coefficient among the repeat runs of the BRDC samples pool was 0.93 and 0.93, respectively, suggesting the reliability of the results of the cohort samples.

### Proteomic and Phosphoproteomic Analysis—Data Normalization and Missing Value Imputation

Protein quantification used precursor ions MS (MS1) signal intensities with total ion current (TIC) normalization. For a peptide “‘i”’ from a sample “‘n,”’ its quantitative information was its peak area (Pi) calibrated by the relative TIC of the sample: Pi * (TICa/TICn), where “‘a”’ represents the sample that was randomly chosen as the benchmark. The quantitative information of the top three peptides of a protein was averaged to get the protein‐level relative quantitative information. The normalized TIC intensities of 224 samples were extracted from the PEAKS Online result files to represent the final expression of a particular protein across samples, resulting in a 15032×224 protein‐expression matrix. The expression matrix was then log_2_‐transformed and used in all quantitative analysis. For missing values, more than 50% of the identified proteins were screened in each progression stage and the proteomic was imputed data using the R package “impute” (https://git.bioconductor.org/packages/impute) based on the K‐NN algorithm. When the protein detection rate was < 50%, the missing value was replaced with one‐tenth of the minimum value across our proteome data. The median numbers of the proteins that were imputed by the K‐NN algorithm for the four stages were 1690 in Normal, 1647 in DH, 1837 in DCIS, and 1574 in IDC, respectively. The median missing values of the four types of samples were 54.35%, 56.62%, 53.29%, and 51.54%, respectively. The missing values of each sample ranged from 39.99% to 69.12%. This missing value percentage is comparable with the published studies that ranged from 30.37% to 78.08%.^[^ ^102^, ^103^, ^104^, ^105^ ^]^

### Proteomic and Phosphoproteomic Analysis—Whole‐Exome Sequencing

WES were all performed by Novogene Co., LTD. DNA from FFPE tumor tissue samples were collected and used for WES, and matched germline DNA was obtained from non‐tumor tissue samples. One hundred and sixteen samples were analyzed by WES, including 79 BRDC samples (15 DH, 32 DCIS, and 32 IDC samples) and 37 paired adjacent normal tissue samples. The 37 paired normal samples included 8 normal samples adjacent to DH, 13 normal samples adjacent to DCIS, and 16 normal samples adjacent to IDC. As previously described,^[^ ^106^, ^107^, ^108^, ^109^ ^]^ a normal sample pool based on the 37 paired normal samples was constructed and the somatic mutation calling against this normal sample pool was performed. Paired‐end sequencing (PE150) was performed on an Illumina HiSeq with a 131.1× target depth (mean) and 14.95 G volume (mean) of 79 raw data (Table S2, Supporting Information).

### Proteomic and Phosphoproteomic Analysis—DNA Extraction

Total DNA from 79 samples of BRDC progression were extracted using QIAamp DNA Mini Kit (QIAGEN, Hilden, Germany) according to the manufacturer's instructions. DNA degradation and contamination were monitored on 1% agarose gels. DNA concentration was measured by Qubit DNA Assay Kit in Qubit 2.0 Fluorimeter (Invitrogen, CA, USA).

### Proteomic and Phosphoproteomic Analysis—Library Preparation

A total amount of 0.6 µg genomic DNA per sample was used as input for DNA sequencing. Sequencing libraries were generated by using Agilent SureSelect Human All Exon V6 kit (Agilent Technologies, CA, USA) following manufacturer's recommendations and barcodes were added to each sample. Briefly, fragmentation was carried out using a hydrodynamic shearing system (Covaris, Massachusetts, USA) to generate 180–280 bp fragments. The remaining overhangs were converted into blunt ends via exonuclease/polymerase activities. Following adenylation of the 3′ ends of DNA fragments, adapter oligonucleotides were ligated. DNA fragments with ligated adapter molecules at both ends were selectively enriched in a PCR reaction. Following the PCR reaction, libraries were hybridized with the liquid phase via a biotin‐labeled probe following which magnetic beads with streptomycin were utilized to capture the exons of genes. Captured libraries were enriched via a PCR reaction to add index tags in preparation for sequencing. Products were purified using an AMPure XP system (Beckman Coulter, Beverly, USA) and quantified using the Agilent high sensitivity DNA assay on the Agilent Bioanalyzer 2100 system.

### Proteomic and Phosphoproteomic Analysis—Clustering and Sequencing

Clustering of index‐coded samples was performed on a cBot Cluster Generation System using a HiSeq PE Cluster Kit (Illumina) according to the manufacturer's instructions. After cluster generation, the DNA libraries were sequenced on the Illumina HiSeq platform and 150 bp paired‐end reads were generated.

### Whole‐Exome Sequencing Data Analysis—Quality Control

The original fluorescence image files obtained from the HiSeq platform were transformed to short reads (raw data) by base calling, following which these short reads were recorded in FASTQ format, which contains sequence information and corresponding sequencing quality information. Sequence artifacts, including reads containing adapter contamination, low‐quality nucleotides, and unrecognizable nucleotides (N), undoubtedly set the barrier for the subsequent reliable bioinformatics analysis. Hence, quality control is an essential step that must be applied to guarantee meaningful downstream analysis.

The data processing steps were as follows:

- Paired reads were discarded if either read contained adapter contamination (>10 nucleotides aligned to the adapter, allowing ≤ 10% mismatches).
- Paired reads were discarded if more than 10% of bases are uncertain.
- Paired reads were discarded if the proportion of low‐quality (Phred quality<5) bases is either read was over 50%.

All downstream bioinformatics analyses were based on high‐quality clean data, which were retained after these steps. At the same time, QC statistics including total read number, raw data, raw depth, sequencing error rate, percentage of reads with Q30 (the percentage of bases with Phred‐scaled quality scores greater than 30), and GC content distribution were calculated and summarized.

### Whole‐Exome Sequencing Data Analysis—Reads Mapping to Reference Sequence

Valid sequencing data were mapped to the reference human genome (UCSC hg19) using Burrows‐Wheeler aligner (BWA) software^[^ ^110^ ^]^ to obtain the original mapping results stored in BAM format. If one read, or one paired read, was mapped to multiple positions, the strategy adopted by the BWA was to choose the most likely placement. If two or more most likely placements were present, the BWA picked one randomly. Then, SAMtools^[^ ^111^ ^]^ and Picard (http://broadinstitute.github.io/picard/) were used to sort BAM files and perform duplicate marking, local realignment, and base quality recalibration to generate final BAM files for computation of the sequence coverage and depth. The mapping step was very difficult due to mismatches, including true mutations and sequencing errors, and duplicates resulting from PCR amplification. These duplicate reads were uninformative and should not be considered as evidence for variants. Picard was used to mark these duplicates for the follow‐up analysis.

### Whole‐Exome Sequencing Data Analysis—Variant Calling

Samtools mpileup and bcftools were used to perform variant calling and identify SNPs and InDels. Somatic SNP variant calls were assessed using MuTect,^[^ ^112^ ^]^ and the Indels variant calls were assessed using Strelka^[^ ^113^ ^]^ with default options. The resulting somatic mutations were annotated using the ANNOVAR RefSeq gene‐based annotation.

### Whole‐Exome Sequencing Data Analysis—Somatic Copy Number Alteration (CNA)

Exome‐based somatic copy number alteration (CNA) was called by following somatic CNV calling pipeline in GATK's (GATK 4) Best Practice. The results of this pipeline and segment files of every 1000 were input in GISTIC2,^[^ ^114^ ^]^ to identify significantly amplified or deleted focal‐level and arm‐level events, with a Q value < 0.1 considered significant. A log_2_ ratio cutoff 1 was used to define CNA amplification and deletion. The arm‐level copy number change was further summarized based on a weighted sum approach,^[^ ^115^ ^]^ in which the segment‐level log_2_ copy ratios for all the segments located in the given arm were added up with the length of each segment being weighted. To exclude false positives as much as possible, relatively stringent cutoff thresholds were used with the following parameters: ‐ta 0.1 ‐tb 0.1 ‐brlen 0.98 ‐conf 0.9. Other parameters were the same as default values.

### Whole‐Exome Sequencing Data Analysis—Analysis of Significantly Mutated Genes

Filtered mutations (including SNV and indel) were further used to identify significantly mutated genes by MutSigCV (https://software.broadinstitute.org/cancer/cga/mutsig, ver. 1.4) with default parameters. Final MutSigCV P values were converted to q values using the method of Benjamini and Hochberg,^[^ ^116^ ^]^ and genes with q ≤ 0.1 were declared to be significantly mutated.

### Whole‐Exome Sequencing Data Analysis—Effects of Copy Number Alterations

CNA affecting protein abundance in either “*cis*” (within the same aberrant locus) or “*trans*” (remote locus) mode were visualized by multiOmicsViz (R package).^[^ ^42^, ^43^, ^44^ ^]^ Spearman's correlation coefficients and associated multiple‐test *p* values were calculated.

### Whole‐Exome Sequencing Data Analysis—Gain of Neo‐Mutations

The mutation frequency was estimated by the ratio of the number of mutated samples versus the number of total samples.^[^ ^105^ ^]^ In our study, the neo‐mutations represented the mutations appearing at a certain stage, but was not identified in earlier stages. For example, *TP53* mutation was first detected in the DCIS stage, but not in the DH stage, indicating *TP53* mutation was the neo‐mutation of the DCIS stage.

### Whole‐Exome Sequencing Data Analysis—Mutational Signature Analysis

Mutation signatures were jointly inferred for 79 tumors using the R package sigminer.^[^ ^24^ ^]^ The sigminer approach (https://github.com/ShixiangWang/sigminer) was used to extract the underlying mutational signatures. The 96 mutation vectors (or contexts) generated by somatic SNVs based on six base substitutions (C > A, C > G, C > T, T > A, T > C, and T > G) within 16 possible combinations of neighboring bases for each substitution were used as input data to infer their contributions to the observed mutations. Sigminer using a nonnegative matrix fac‐torization (NMF) approach was applied to decipher the 96 × 79 (i.e., mutational context‐by‐sample) matrix for the 30 known COSMIC cancer signatures (https://can‐cer.sanger.ac.uk/cosmic/signatures) and infer their exposure contributions.

### RNA‐Seq—RNA Extraction

RNA was extracted from tissues by using TRIzol reagent kit (Ambion, Invitrogen, USA) according to the reagent protocols. The concentration and RNA integrity were then determined by using a NanoDrop ND‐1000 spectrophotometer (NanoDrop Technologies, Wilmington, USA) and an Agilent 2100 Bioanalyzer (Agilent, CA, USA). RNA samples exhibiting an RNA integrity number (RIN) greater than 6.0 were included in the study. For library preparation of RNA sequencing, a total amount of 2 µg RNA per sample was used as the input material for the RNA sample preparations. Sequencing libraries were generated using NEBNext UltraTM RNA Library Prep Kit for Illumina (#E7530L, NEB, USA) following the manufacturer's recommendations and index codes were added to attribute sequences to each sample. Briefly, mRNA was purified from total RNA using poly‐T oligo‐attached magnetic beads. Fragmentation was carried out using divalent cations under elevated temperature in NEBNext First Strand Synthesis Reaction Buffer (5X). First strand cDNA was synthesized using random hexamer primer and RNase H. Second strand cDNA synthesis was subsequently performed using buffer, dNTPs, DNA polymerase I and RNase H. The library fragments were purified with QiaQuick PCR kits and eluted with EB buffer, followed by terminal repair, A‐tailing and adaptor addition. The aimed products were retrieved and PCR was performed, then the library was completed. The RNA concentration of the library was measured using Qubit RNA Assay Kit in Qubit 3.0 and then it was diluted to 1 ng mL^−1^. Insert size was assessed using the Agilent Bioanalyzer 2100 system (Agilent Technologies, CA, USA), and qualified insert size was accurately quantified using StepOnePlusTM Real‐Time PCR System (Library valid concentration > 10 nm). The clustering of the index‐coded samples was performed on a cBot cluster generation system using HiSeq PE Cluster Kit v4‐cBot‐HS (Illumina) according to the manufacturer's instructions. After cluster generation, the libraries were sequenced on an Illumina platform and 150 bp paired‐end reads were generated.

### RNA‐Seq—RNA‐Seq Data Analysis

RNA‐Seq reads were adaptor trimmed and the data quality was assessed with the FastQC (ver. 0.11.7) software before any data filtering criteria was applied. Reads were mapped onto the human reference genome (GRCh38.p12 assembly) by using HISAT2 software (ver. 2.0.4). The mapped reads were assembled into transcripts or genes by using StringTie software (ver. 1.3.4d) and the genome annotation file (hg38_ucsc.annotated.gtf). For quantification purpose, the relative abundance of the transcript/gene was measured by a normalized metrics, FPKM (Fragments Per Kilobase of transcript per Million mapped reads). Transcripts with an FPKM score above one were retained, resulting in a total of 12563 gene IDs. All known exons in the annotated file were 100% covered.

### Functional Experiments—Antibodies and Reagents

Primary antibodies used in this study included TP53 antibody (Leica Biosystems, Cat# PA0067), ESR1 antibody (Abcam, Cat# ab241557), CD8A antibody (Invitrogen, Cat# PA5‐11453), NR3C1 antibody (Abcam, Cat# ab183127), TIAM1 antibody (Cloud‐clone, Cat# PAC778Hu01), AR antibody (Gene Tech, Cat# GT245202), AKR1C1 antibody (GeneTex, Cat# GTX105620), and ACSS2 antibody (Abcam, Cat# ab133664). There were two inhibitors targeting AKR1C1 used in this study. Aspirin (A2093) was purchased from Sigma‐Aldrich, while dydrogesterone (HY‐B0257A) from MedchemExpress. Tamoxifen (S1238), lapatinib (S2111), etomixir (S8244), IACS‐010759 (S8731) and px‐478 (S7612) were purchased from Selleck. IL‐2‐IN‐1 (245747‐10‐8) was purchased from MedchemExpress.

### Functional Experiments—Cell Culture

Human T47D (HR+, HER2‐), BT‐474 (HR+, HER2+), SKBR‐3 (HR‐, HER2+), and MDA‐MB‐231 (TNBC) BC cell lines were obtained from American Type Culture Collection (ATCC). The HCC1806 cell line (ZQ0769) was obtained from Zhong Qiao Xin Zhou Biotechnology (Shanghai, China). The HCC1937 (CL‐0093) cell line was obtained from Procell Life Science & Technology (Wuhan, China). The MCF10DCIS.COM cell line were kindly provided by Fudan University Shanghai Cancer Center. All cell lines were used within 15 passages. All cell line authentication had been carried out by short tandem repeat method through using Short Tandem Repeat Multi‐Amplification Kit (Microreader 21 ID System), which was used for PCR amplification and PCR products were detected by ABI 3130xl DNA Analyzer (Applied Biosystems). All cell lines had been confirmed without Mycoplasma contamination by using Mycoplasma Stain Assay Kit (Beyotime Institute of Biotechnology). T47D, BT‐474, SKBR‐3, MDA‐MB‐231, HCC1806, HCC1937 cells were cultured in RPMI 1640 Medium (Gibco, Cat# 22 400 089) or DMEM (Gibco, Cat# 11 965 092) supplemented with 10% fetal bovine serum (Gibco, Cat# 10 099 141) and 100 U mL^−1^ penicillin‐streptomycin solution (Gibco, Cat# 15 140 122). MCF10DCIS.COM cells were cultured in specific epithelial culture medium (Procell, Cat# CM‐0525). Cells were maintained in a 5% CO_2_‐humidified atmosphere at 37 °C until ready for use.

### Functional Experiments—RNA Interference

Synthetic oligos were used for siRNA‐mediated silencing of *SREBF2* (5′‐GCUGCAAUUUGUCAGUAAUTT‐3′ and 5′‐GGACAACCCAUAAUAUCAUTT‐3′), *NR3C1* (5′‐GUGGCAAUGUGAAAUUGUATT‐3′ and 5′‐CCCAGGUAAAG‐AGACGAAUTT‐3′), *TIAM1* (5′‐GCGCCUGAAAUUUCUAAUATT‐3′ and 5′‐CCUCCGUACAGUAAUUAUATT‐3′), *AKR1C1* (5′‐UCCAGUGUCUGUAAAG‐CCATT‐3′ and 5′‐GGAGAUGAUCCUCAACAAGTT‐3′), *ACSS2* (5′‐GGACCAGGAUGGCUAUUAC‐3′), and scramble siRNA was used as a control. Cells were transfected with siRNAs using Lipofectamine 2000 Reagent (Invitrogen) according to the manufacturer's protocol. Knockdown efficiency was verified by qRT‐PCR or western blotting.

### Functional Experiments—Construction of AKR1C1 Overexpression, Lentivirus Production and Cell Transduction

For the analysis of AKR1C1 in preventing the apoptosis of tumor cells and promoting migration of tumor cells, stable cell lines overexpressing AKR1C1‐FLAG were constructed. The cDNA of AKR1C1 was cloned into the pLV3‐CMV‐MCS‐3×FLAG‐Puro vector via the unique *EcoRI* site and the neighboring *BamHI* site. For convenient detection, 3×FLAG‐tag encoding sequence (GACTACAAAGACCATGACGGTGATTATAAAGATCATGACATC‐GACTACAAGGATGACGATGACAAGTAG) was inserted before the start codon (ATG) to express the AKR1C1‐FLAG fusion protein. The primers used for plasmid construction as following: forward primer (5′‐3′): GATTCTAGAGCT‐AGCGAATTCgccaccatggattcgaaatatcagtgtgt, reverse primer (5′‐3′): CATGGT‐CTTTGTAGTCGGATCCatattcatcagaaaatggataattagg.

The double‐stranded AKR1C1 DNA was cloned into the pLV3‐CMV‐MCS‐3×FLAG‐Puro vector; 8 µg of packaging plasmids psPAX2: pMD2.G (3:1) and 8 µg of Lenti‐vector containing the target gene were co‐transfected into 2.5×10^6^ HEK293T cells using Lipofectamine 2000 Reagent (Invitrogen). In this transfection method, 500 µL of Opti‐MEM (serum‐free medium) and the plasmid were placed in an empty EP tube and PEI (three times the concentration of plasmid) was added into the medium with vigorous shaking. The mixture was incubated for 15 min. Meanwhile, the cell culture medium was replaced with 5 mL of fresh 10% FBS medium. After 15 min, the mixture was added to the cells, and the fresh medium was replaced after 8 h. After 48–72 h, the transfection was completed. The media containing the lentivirus particles were collected after 48–72 h, and centrifuged at 5000×g for 5 min. The supernatants approximately 1 mL mixed with 1 mL fresh medium were used independently to infect HCC1937 cells; 24 h later, removed the media containing the lentivirus particles and added the fresh medium. After infection, cells were cultured and selected with puromycin for the generation of stably overexpressed cells. Empty pLV3‐CMV‐MCS‐3×FLAG‐Puro vector was used as a negative control.

### Functional Experiments—Western Blot Assay and Antibodies

Total protein extracted from cells using RIPA lysis buffer and equal amounts of the cell protein lysates (30 µg) were separated by 10% SDS‐PAGE and transferred to a hybridization nitrocellulose filter (NC) membrane (Millipore, Darmstadt, Germany). After being blocked with 5% nonfat dry milk in phosphate‐buffered saline and 0.1% Tween 20 solution, membranes were incubated overnight at 4 °C with the following primary antibodies: AKR1C1 (Abcam, China, 1:1000), P38 MAPK (Cell Signaling Technology, China, 1:1000), p‐P38 MAPK (Cell Signaling Technology, China, 1:1000), and β‐Actin (Proteintech, China, 1:1000). The membranes were then incubated with the appropriate secondary antibody for 2 h at room temperature. The specific bands were detected using an ECL detection kit (Applygen, China) and captured on a Molecular Imager ChemiDoc XRS system (Bio‐Rad, Louisville, USA). Relative expression was determined by normalizing with b‐actin using the ImageJ software (Ver. 1.53n, National Institutes of Health, MD, USA).

### Functional Experiments—In Vitro Cell Migration Assays

To examine the cell migration in vitro, the transwell assay and wound healing assay were used. Transwell migration assay was performed in chambers with 8.0 µm polycarbonate membrane (Corning) in a 24‐well transwell plate. A fair amount of differently treated cells was seeded into the upper chamber in serum‐free basic medium, while the lower chamber was filled with completed medium containing 10% FBS. After incubation for 24 h, the migrated cells on the lower side of the well were then fixed by 4% paraformaldehyde, stained with 0.5% crystal violet and photographed in multiple random sight under inverted microscope. The numbers of cells in each sight were counted and the average were there for comparison.

For wound healing assay, cells were seeded in 6‐well plates, reaching nearly 90% cell confluence overnight. Cells were scratched using pipette tips and washed twice with PBS. Then, the cells were cultured in serum‐free medium for 24 h. The scratches were marked and captured under inverted microscope at 0 h and 24 h. Subsequently, the width of scratches was analyzed by ImageJ software. The wound‐healing rate was defined as the ratio of width deviation between 0 h and 24 h to the width at 0 h, the average of the rate indicated the migration ability of cells.

### Functional Experiments—Quantitative Real‐Time PCR (qRT‐PCR)

Certain amount of total RNA isolated from cells using the TRIzol reagent (Takara) were reverse‐transcribed into cDNA. Quantitative real‐time PCR analysis was performed in one step with Hieff qRCR SYBR Green Master Mix (Yeasen Biotechnology). Quantification of gene expression was calculated as R = 2‐ΔΔCt, with *GAPDH* used as a reference gene. Specific primers of target genes were synthesized by Sangon Biotech and the sequences of all primers were as follows: GAPDH: forward primer: 5′‐ATCATCCCTGCCTCTACTGG‐3′, reverse primer: 5′‐GTCAGGTCCACCACTGACAC‐3′; SREBF2: forward primer (5′‐3′): ATGGGCAGCAGAGTTCCTTC, reverse primer (5′‐3′): CGACAGTAGCAGGT

CACAGG; LSS: forward primer (5′‐3′): TCAGTGCAGCTCCCTGACG, reverse primer (5′‐3′): CATAGTTGAGCGCAGTCCCA; SC5D: forward primer (5′‐3′): GACGGTGATTTTCGTGTCCC, reverse primer (5′‐3′): ATGAGCCGCCAATC

CTATCC; MVD: forward primer (5′‐3′): ATCAAGTACTGGGGCAAGCG, reverse primer (5′‐3′): CAAATCCGGTCCTCGGTGAA; NR3C1: forward primer (5′‐3′): GGCGGGAGAAGACGATTCAT, reverse primer (5′‐3′): ACTGGGGCT

TGACAAAACCA; CX3CL1: forward primer (5′‐3′): CGGGCGTGACTGGTTCC

TC, reverse primer (5′‐3′): CGTGGCGGCAGTGGAGAC; CXCL12: forward primer (5′‐3′): AGATGCCCATGCCGATTCTT, reverse primer (5′‐3′): AGGGCACAGTTTGGAGTGTT; TIAM1: forward primer (5′‐3′): ATGAGTTCT

GTGAGTCCGTGAAGG, reverse primer (5′‐3′): CCTCTTTCCCGCTGACTGA

TGG; AKR1C1: forward primer (5′‐3′): CATGCCTGTCCTGGGATTT, reverse primer (5′‐3′): AGAATCAATATGGCGGAAGC.

### Functional Experiments—^13^C Labeling Acetyl‐CoA Detection

For the ^13^C labeling acetyl‐CoA detection, 2 mmol L^−1^ palmitic acid (U‐^13^C) were used to treat cells for 12 h and ^13^C‐labeled acetyl‐CoA derived from ^13^C palmitate was detected using an LC‐MS/MS method as previously described.^[^ ^117^ ^]^

### Functional Experiments—Measurement of ROS Production

Intracellular ROS levels were measured with the Reactive Oxygen Species Assay Kit (Yeasen, Shanghai) based on DCFH‐DA (2′,7′‐dichlorodi‐hydrofluorescein diacetate) according to the manufacturer's protocol. 24 h after transfected with siRNA or negative control, Cells were incubated with 10 µm DCF‐DA for 30 min at 37 °C and then analyzed using the FACSCalibur flow cytometer (BD Biosciences, San Jose, CA).

### Functional Experiments—CCK‐8 Assay

The Cell Counting Kit‐8 (CCK‐8, ShareBio) was performed to assess the cell viability of breast cancer cells undergoing different treatments 26. Cells were plated into 96‐well plates at an appropriate density. After adherence, the cells were treated with inhibitors of AKR1C1 for 96 h. The WST‐8 was added into the culture medium and incubated at 37 °C at intervals of 24 h. The optical density at 450 nm (OD450) was recorded and the growth curves were drawn accordingly.

### Functional Experiments—Flow Cytometry Analysis with Annexin V‐PI Staining

Flow cytometry was performed to analyze cell apoptosis 26. For cell apoptosis analysis, ∼10^6^ cells were harvested from culture dishes using trypsin (without EDTA). After two washes with cold PBS, cells were resuspended in 100 µL 1 × Binding Buffer (Annexin V‐FITC/PI Apoptosis Detection Kit, YEASEN, Shanghai, China). Cells were stained with 5 µL annexin V‐FITC and 10 µL PI staining solution (Annexin VFITC/PI Apoptosis Detection Kit) in the dark, at room temperature for 15 min. Following this incubation, 400 µL 1 × Binding Buffer was added to each sample and then kept on ice until analysis (within 1 h).

### Functional Experiments—Immunofluorescence

Cultured cells were washed in phosphate‐buffered saline (PBS) and fixed in 4% paraformaldehyde (Sigma, 441 244) in PBS for 15 min at room temperature. Subsequently, they were permeabilized for 10 min with 0.25% Triton X‐100 in PBS, and blocked with 10% goat serum albumin (Beyotime, China) in PBS for 30 min at 37 °C. Primary and secondary antibody incubations (both in 2.5% BSA) were overnight at 4 °C and 2 h at 37 °C, respectively. Primary antibodies used: Anti‐Androgen receptor antibody (Proteintech, Cat# ab66747‐1‐Ig, 1:200). The following secondary antibodies (all from Thermo Fisher Scientific): Donkey anti‐Rabbit Alexa Fluor 488 (Cat# A21206) were used at 1:250, and DAPI (Beyotime, Cat# C1005) was used at 1:5000. Coverslips were mounted in Antifade Mounting Medium (Beyotime, Cat# P0126).

### Functional Experiments—Measurement of OCR and ECAR

The oxygen consumption rate (OCR) and the extracellular acidification rate (ECAR) were measured with the Seahorse XF Cell Mito Stress Test Kit using the Seahorse XF Analyzer (Agilent, Santa Clara, CA, USA). Briefly, the day prior to analysis, 1.5 × 10^4^ breast cancer cells were seeded into a 24‐well XF24 cell culture microplate. Cells were washed and incubated in the Seahorse Bioscience assay medium contain 4500 mg L^−1^ glucose, 4 mm L‐glutamine, and 1 mm sodium pyruvate in a non‐CO2 incubator at 37 °C for 1 h before commencing the assay. Mitochondrial function was analyzed by performing the oxygen consumption rate (OCR) measurement at baseline and following treatment with the mitochondrial inhibitor oligomycin (1 µm), mitochondrial uncoupler FCCP (1 µm), or respiratory chain inhibitors rotenone and antimycin A (1 µm). Extracellular acidification rate (ECAR) was measured concurrently with OCR.

### Functional Experiments—Immunohistochemistry Staining and Evaluation

Formalin‐fixed, paraffin‐embedded tissue sections of 10 µm thickness were stained in batches for detecting TP53, ESR1, NR3C1, CD8A, TIAM1, AR, and AKR1C1 in a central laboratory at the Zhongshan Hospital according to standard automated protocols. Deparaffinization and rehydration were performed, followed by antigen retrieval and antibody staining. IHC was performed using the Leica BOND‐MAX auto staining system (Roche). Antibody was introduced, followed by detection with a Bond Polymer Refine Detection DS9800 (Bond). Slides were imaged using an OLYMPUS BX43 microscope (OLYMPUS) and processed using a Scanscope (Leica). The IHC evaluation was analyzed using an IHC profiler compatible plugin with integrated options for the quantitative analysis of digital IHC images stained for cytoplasmic or nuclear proteins.^[^ ^118^ ^]^ Moreover, the intensity of the cytoplasmic staining and the percentage of positively stained tumor cells were also scored numerically.

Mutant TP53: mouse monoclonal anti‐P53 (1:800, Leica Biosystems, Cat# PA0067); ESR1: mouse monoclonal anti‐ESR1 (1:500, Abcam, Cat# ab241557); NR3C1: rabbit monoclonal anti‐NR3C1 (1:1000, Abcam, Cat# ab183127); CD8A: rabbit polyclonal anti‐CD8A (1:50, Invitrogen, Cat# PA5‐11453); TIAM1: rabbit polyclonal anti‐TIAM1 (1:100, Cloud‐clone, Cat# PAC778Hu01); AR: rabbit polyclonal anti‐AR (1:200, Gene Tech, Cat# GT245202); AKR1C1: rabbit polyclonal anti‐AKR1C1 (1:500, GeneTex, Cat# GTX105620).

### Functional Experiments—Enzyme‐Linked Immunosorbent Assay (ELISA)

The protein levels of CXCL12 and CX3CL1 in cell lysate supernatant and CM were measured by using human ELISA kits (Sangon Biotech, cat# D711027‐0096 and cat# D711433‐0096) according to the manufacturer's instructions. The protein levels were calculated based on the standard curve.

### Functional Experiments—CD8+ T cells Isolation

CD8+ T cells were isolated from PBMCs of BC patients prepared as described, by positive selection using the EasySep Human CD8+ T Cell Isolation Kit (StemCell, Cat#17 853) according to the manufacturer's protocol. The purity of enriched cells was assessed via flow cytometry as the proportion of CD3+ and CD8+ cells with purities ranging from 91% to 93%.

### Functional Experiments—Two‐Chamber CD8+ T Cells Migration Assays

The procedure of two‐chamber migration assays was previously described.^[^ ^119^ ^]^ CD8+ T cells migration was evaluated using a 24‐well, 5.0 µm pore size Transwell plate (JET, TCS0040). CD8+ T cells were activated with Immunocult Human CD3/CD28 T Cell Activator (StemCell, Cat#10 971) for 72 h and washed once with PBS. An aliquot (100 µL) of the cell suspension containing 5 × 10^5^ CD8+ T cells was added in the upper chamber of the transwell. A 1:1 mixture of ImmunoCult‐XF T Cell Expansion Medium (StemCell, Cat#10 981) and *NR3C1*‐knockdown MCF10DCIS.COM cells CM or MCF10DCIS.COM cells CM was added to the lower chamber as the chemoattractant. The migrated cells in the lower chamber were counted after 12 h.

### Quantification and Statistical Analysis—Quality Control and Assessment of LC‐MS/MS Data

The density plot of the normalized intensities of the proteins identified in each sample showed that all samples passed the quality control with an expected unimodal distribution (dip statistic test).

### Quantification and Statistical Analysis—Differential Protein Analysis

Student's *t*‐test was used to examine whether proteins were differentially expressed between DCIS and DH, or patients with different *TP53* mutation status. Upregulated or downregulated proteins in tumors were defined as proteins differentially expressed in DCIS compared with DH (fold change > 1.2 or < 0.8, Student's *t‐*test, *p* < 0.05). Student's *t‐* test was used to examine whether proteins were differentially expressed between DCIS_Pure and DH. Upregulated or downregulated proteins in DCIS_Pure were defined as proteins differentially expressed compared with DH (FC > 2 or < 0.5, Student's *t‐*test, *p* < 0.05).

### Quantification and Statistical Analysis—Gene Set Enrichment Analysis (GSEA)

GSEA was performed by the GSEA software (http://software.broadinstitute.org/gsea/index.jsp).^[^ ^120^ ^]^ Gene sets including KEGG, GO Biological Process (BP), Reactome, and HALLMARK downloaded from the Molecular Signatures Database (MSigDB v7.1, http://software.broad.institute.org/gsea/msigdb/index.jsp) were used.

### Quantification and Statistical Analysis—Pathway Scores and Correlation Analysis

Single‐sample gene set enrichment analysis (ssGSEA)^[^ ^29^ ^]^ was utilized to obtain pathway scores for each sample based on proteomic and phosphoproteomic data using the R package GSVA.^[^ ^30^ ^]^ Correlations between the pathway scores and other features were determined using Spearman's correlation. Inferred activity was performed using ssGSEA implemented in the R package GSVA with a minimum gene set size of 10. The transcriptional targets of SREBF2 and ESR1 transcription factors were collected from the ENCODE and CHEA transcription factor targets datasets,^[^ ^32^, ^121^ ^]^ and used to infer the activities of SREBF2 and ESR1 via ssGSEA, respectively.

### Quantification and Statistical Analysis—Estimation of Multigene Proliferation Scores (MGPS)

To calculate MGPS, the mean expression level was calculated for all genes previously identified as exhibiting regulation during the cell cycle.^[^ ^122^ ^]^

### Quantification and Statistical Analysis—Phosphoproteomic Data Analysis

Phosphoproteome MS raw files were searched against the human RefSeq protein database (27414 proteins, version 04/07/2013) using Proteome Discoverer (ver. 2.3) with a Mascot^[^ ^123^ ^]^ (version 2.3.01) engine with a percolator.^[^ ^124^ ^]^ Carbamidomethyl cysteine was used as a fixed modification, and oxidized methionine, protein N‐term acetylation, and phospho (S/T/Y) were set as variable modifications. The false discovery rate (FDR) of peptides and proteins was set at 1%. The tolerance for spectral searches a mass tolerance of 20 ppm for the precursor. The maximum number of missing cleavage sites was set at 2. For phosphosite localization, ptmRS^[^ ^125^ ^]^ was used to determine phosphosite confidence and a phosphosite probability > 0.75 was used for further analysis.

### Statistical Analysis

In this study, the StandardScaler (namely z‐score) and MinMaxScaler were used to transform the data for analysis or display the data. If necessary, any observations that are more than 1.5 interquartile range (IQR) below the first quartile or more than 1.5 IQR above the third quartile were considered outliers. To handle the categorical data, the Fisher exact test or the Chi‐square test was used. To handle the continuous data for the comparison analyses of two groups (such as the comparison analyses of proteome data between two stages of the BRDC progression), the Student's *t*‐test or the Rank sums test was used. To handle the continuous data for the comparison analyses of multiple groups (such as the comparison analyses of proteome data among all the BRDC stages), the ANOVA test or the Kruskal test was used. To account for multiple tests, the *p* values were adjusted using the Benjamin‐Hochberg FDR correction. To evaluate the correlation of two continuous variables, Spearman's correlation or Pearson's correlation analysis was used. A *p*‐value or FDR less than 0.05 was considered statistically significant. Unless the statement, the two‐sided testing was used for all the statistical inference analyses. All the analyses of clinical data were performed in R (version 4.0.2) and python (version 3.9.15).

### Ethics Approval and Consent to Participate

The present study was carried out to comply with the ethical standards of Helsinki Declaration II and approved by the Institution Review Board of Fudan University Zhongshan Hospital (B2019‐200R).

## Conflict of Interest

The authors declare no conflict of interest.

## Author Contributions

G.X., J.Y., J.L., M.Z., J.X., and M.H. contributed equally to this work. C. D., Y. H., X. L., C. X., H. Z., and G. X. contributed to conceptualization; G. X., J. X., J. Y., M. Z, M. H., R. Z., and Z. S. performed experiments and data collection; J. L., G. X., J. Z., J. F., S. T., and C.D. curated the data; G. X., J. L., and M. H. analyzed the data; G. X., J. L., M. H., P.R., and Y. L. visualized the study; J. Y., M. Z, Y. H., C. X., H. Z., X. L., J. Z., J. C., and C. D. contributed to patient sample management and QC; G. X., R. Z., J. X., and C.D. wrote the manuscript. All authors read and approved the final manuscript.

## Supporting information



Supporting Information

Supplementary Table 1

Supplementary Table 2

Supplementary Table 3

Supplementary Table 4

Supplementary Table 5

Supplementary Table 6

## Data Availability

The raw WES data and the raw RNA‐seq data have been deposited in the National Genomics Data Center (GSA) database under accession code HRA008164. Proteomic and phosphoproteomic raw datasets are available through the iProx Consortium (https://www.iprox.org/) with the subproject ID IPX0004887000 or the Firmiana platform (http://www.firmiana.org/login/). In addition, we have provided detailed codes and the statistical methods used for data analysis in our study to GitHub (https://github.com/Jiacheng‐Lyu/BRDC).
