# Supplementary material for: Proteogenomic Landscape of Breast Ductal Carcinoma Reveals Tumor Progression Characteristics and Therapeutic Targets
Source: Adv Sci (Weinh). 2024 Oct 17;11(46):2401041. doi: 10.1002/advs.202401041 (PMC11633542; doi:10.1002/advs.202401041)
Supplement: Supplementary file 1 — Supporting Information [file ADVS-11-2401041-s004.pdf]

ADVANCED SCIENCE
Open Access

Supporting Information

for *Adv. Sci.*, DOI 10.1002/advs.202401041

Proteogenomic Landscape of Breast Ductal Carcinoma Reveals Tumor Progression Characteristics and Therapeutic Targets

Ganfei Xu, Juan Yu, Jiacheng Lyu, Mengna Zhan, Jie Xu, Minjing Huang, Rui Zhao, Yan Li, Jiajun Zhu, Jinwen Feng, Subei Tan, Peng Ran, Zhenghua Su, Xinhua Liu, Jianyuan Zhao, Hongwei Zhang, Chen Xu*, Jun Chang*, Yingyong Hou* and Chen Ding**

Proteogenomic Landscape of Breast Ductal Carcinoma Reveals Tumor Progression Characteristics and Therapeutic Targets

Ganfei Xu,[1,4] Juan Yu,[1,4] Jiacheng Lyv,[1,4] Mengna Zhan,[1,4] Jie Xu,[1,4] Minjing Huang,[1,4] Rui Zhao,[2] Yan Li,[1] Jiajun Zhu,[1] Jinwen Feng,[1] Subei Tan,[1] Peng Ran,[1] Zhenghua Su,[1] Xinhua Liu,[1] Jianyuan Zhao,[2] Hongwei Zhang,[1,*] Chen Xu,[1,*] Jun Chang,[1,*] Yingyong Hou,[1,*] and Chen Ding,[1,3,5*]

[1] State Key Laboratory of Genetic Engineering, School of Life Sciences, Human Phenome Institute, Department of Pathology, Zhongshan Hospital, Fudan University, Shanghai 200433, China

[2] Institute for Developmental and Regenerative Cardiovascular Medicine, MOE-Shanghai Key Laboratory of Children's Environmental Health, Xinhua Hospital, Shanghai Jiao Tong University School of Medicine, Shanghai 200092, China

[3] Departments of Cancer Research Institute, Affiliated Cancer Hospital of Xinjiang Medical University, Xinjiang Key Laboratory of Translational Biomedical Engineering, Urumqi 830000, P. R. China

[4] These authors contribute equally

[5] Lead Contact

*Correspondence: chend@fudan.edu.cn (C.D.), hou.yingyong@zs-hospital.sh.cn (Y. H.), jchang@fudan.edu.cn (J.C.), xu.chen@zs-hospital.sh.cn (C.X.), zhang.hongwei@zs-hospital.sh.cn (H. Z.)

Supplementary Figure 1

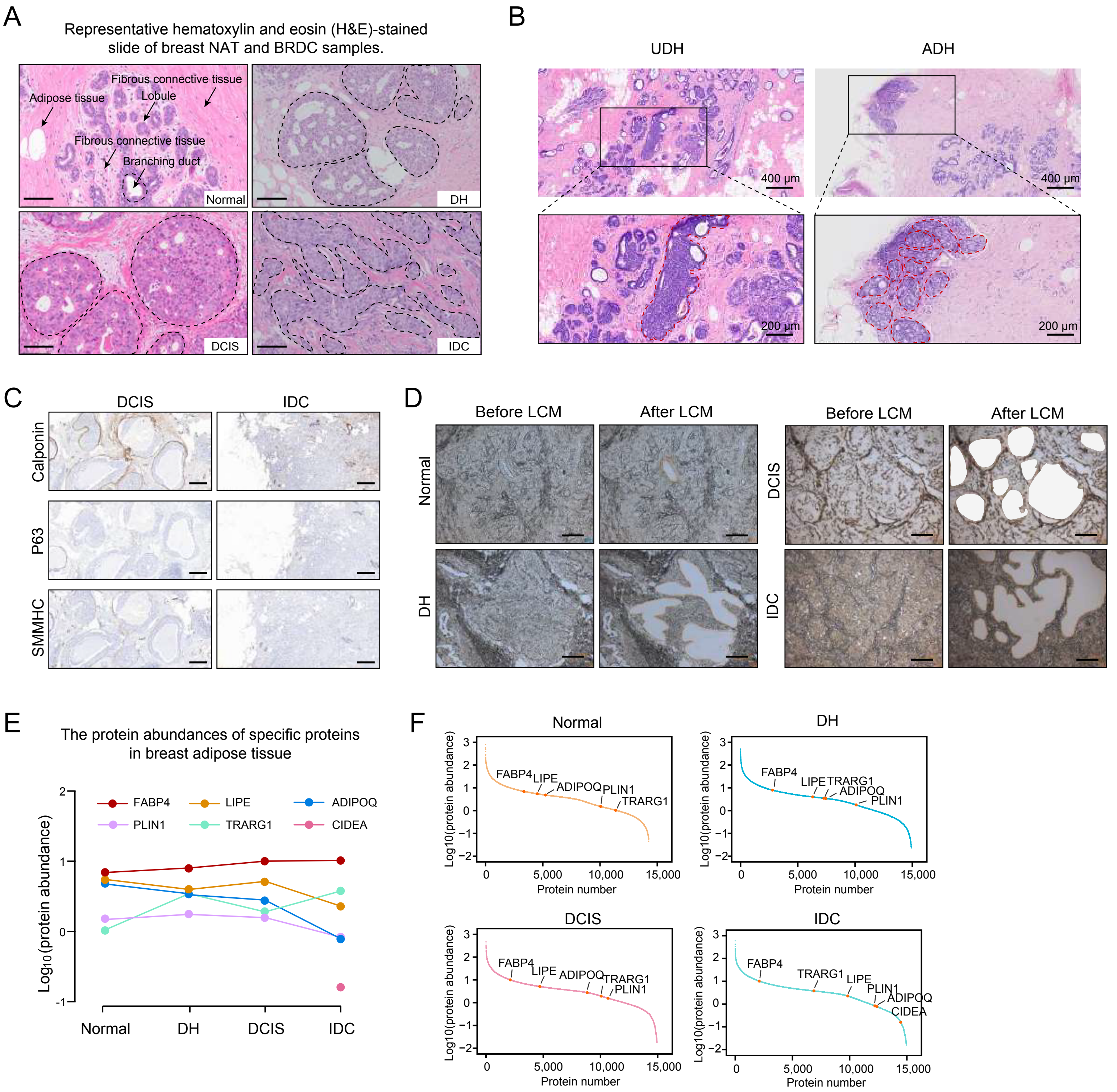

Figure S1. Proteogenomic Profiling of BRDC Progression, related to Figure 1

(A) Representative hematoxylin and eosin (H&E)-stained slide of Nromal and BRDC samples (Scale bars = 100 μm). The black dotted circle indicates the sampling area circled by the pathologist.

(B) Representative hematoxylin and eosin (H&E)-stained slides of usual ductal hyperplasia (UDH) and atypical ductal hyperplasia (ADH) (Scale bars = 200 μm). The red dotted circle indicates the sampling area circled by the pathologist.

(C) Representative IHC images of on a panel of myoepithelial markers on DCIS and IDC tissues. Scale bars = 400 μm.

(D) The microdissection of the different stages samples of BRDC progression. Scale bars = 1000 μm.

(E and F) The abundance of the specific proteins of breast adipose tissue in different stages of BRDC progression.

Supplementary Figure 2

A

	Cosine similarity	COSMIC signatures	Description
DH	0.835	Signature 30	Defective DNA base excision repair due to NTHL1 mutations
	0.69	Signature 42	Haloalkane exposure
DCIS	0.818	Signature 30	Defective DNA base excision repair due to NTHL1 mutations
	0.71	Signature 7a	Ultraviolet light exposure
	0.749	Signature 6	Defective DNA mismatch repair
	0.579	Signature 2	Activity of APOBEC family of cytidine deaminases
IDC	0.807	Signature 30	Defective DNA base excision repair due to NTHL1 mutations
	0.738	Signature 7a	Ultraviolet light exposure
	0.72	Signature 7b	Ultraviolet light exposure
	0.633	Signature 10b	Polymerase epsilon exonuclease domain mutations

B

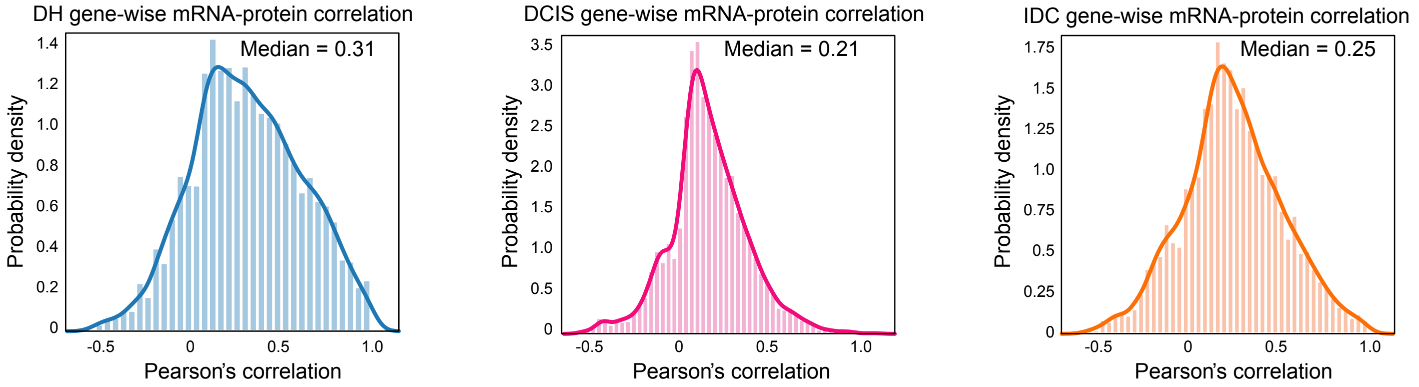

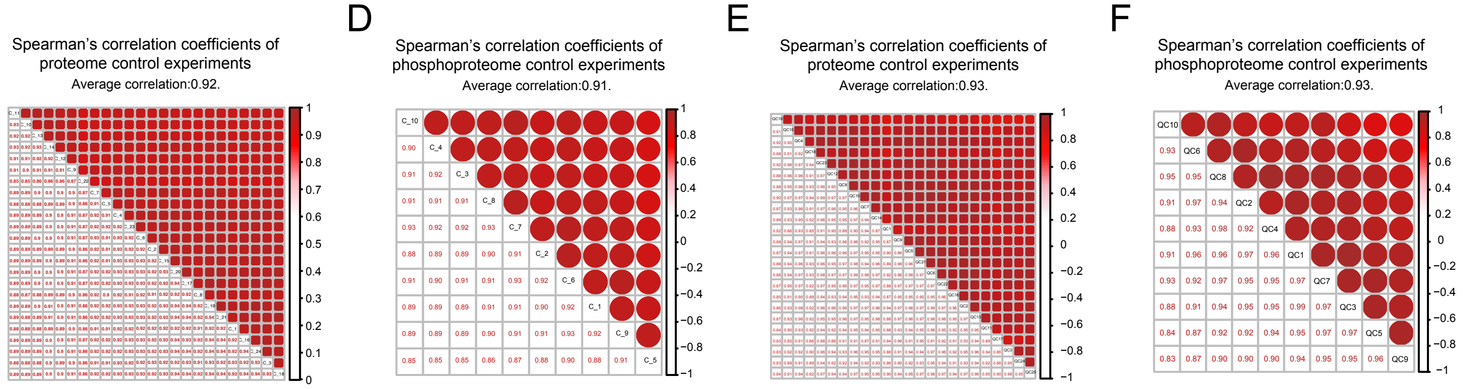

G

Spearman's correlation coefficients of stages in the BRDC progression.

Stage	Spearman's R (mean)
Normal	0.89
DH	0.85
DCIS	0.83
IDC	0.78

H

Spearman's correlation coefficients of stages in the BRDC progression.

Stage	Spearman's R (mean)
Normal	0.91
DH	0.89
DCIS	0.83
IDC	0.83

I

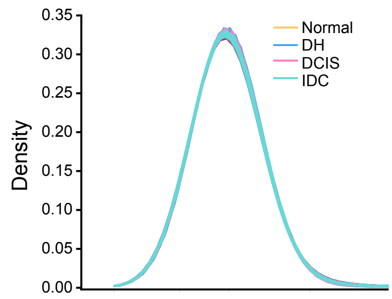

J

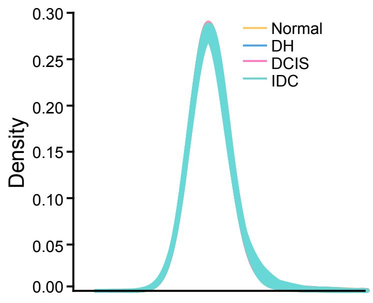

K

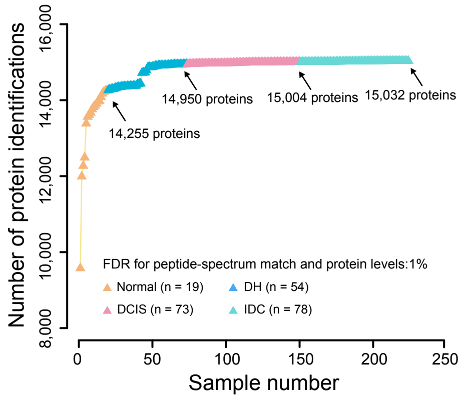

L

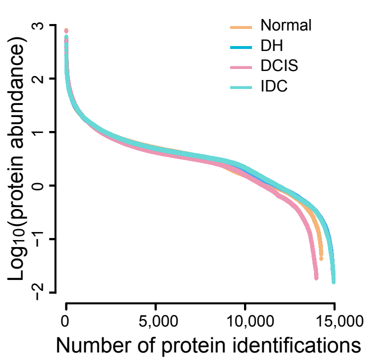

M

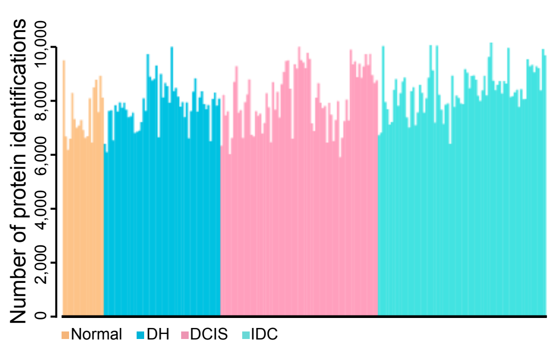

Figure S2. Proteogenomic Profiling of BRDC Progression, related to Figure 1

(A) Mutational signatures in the BRDC progression.

(B) mRNA-protein correlation in different stages of BRDC progression. Left: mRNA-protein correlation in DH. Middle: mRNA-protein correlation in DCIS. Right: mRNA-protein correlation in IDC.

(C and D) Longitudinal quality control of mass spectrometry using tryptic digest of HEK293T cells. The bottom-left half of the panel represents the Spearman's correlation coefficients of the samples.

(E and F) Longitudinal quality control of mass spectrometry using the BRDC samples pool. The bottom-left half of the panel represents the Spearman's correlation coefficients.

(G) The table chart showing the (Spearman's) correlation coefficients (mean) of stages of the proteomes in the histopathological stages of the BRDC progression.

(H) The table chart showing the (Spearman's) correlation coefficients (mean) of stages of the phosphoproteomes in the histopathological stages of the BRDC progression.

(I) Distribution of protein abundances in the different stages of BRDC progression by a density plot. All of the samples passed proteomic quality control.

(J) Distribution of phosphoprotein abundances in the different stages of BRDC progression by a density plot. All of the samples passed proteomic quality control.

(K) The cumulative number of protein identifications of 224 samples.

(L) Overview of the proteomics profile of BRDC patients. Shown are the dynamics of protein abundances identified in the different stages of BRDC progression.

(M) The number of proteins identified in each sample.

Supplementary Figure 3

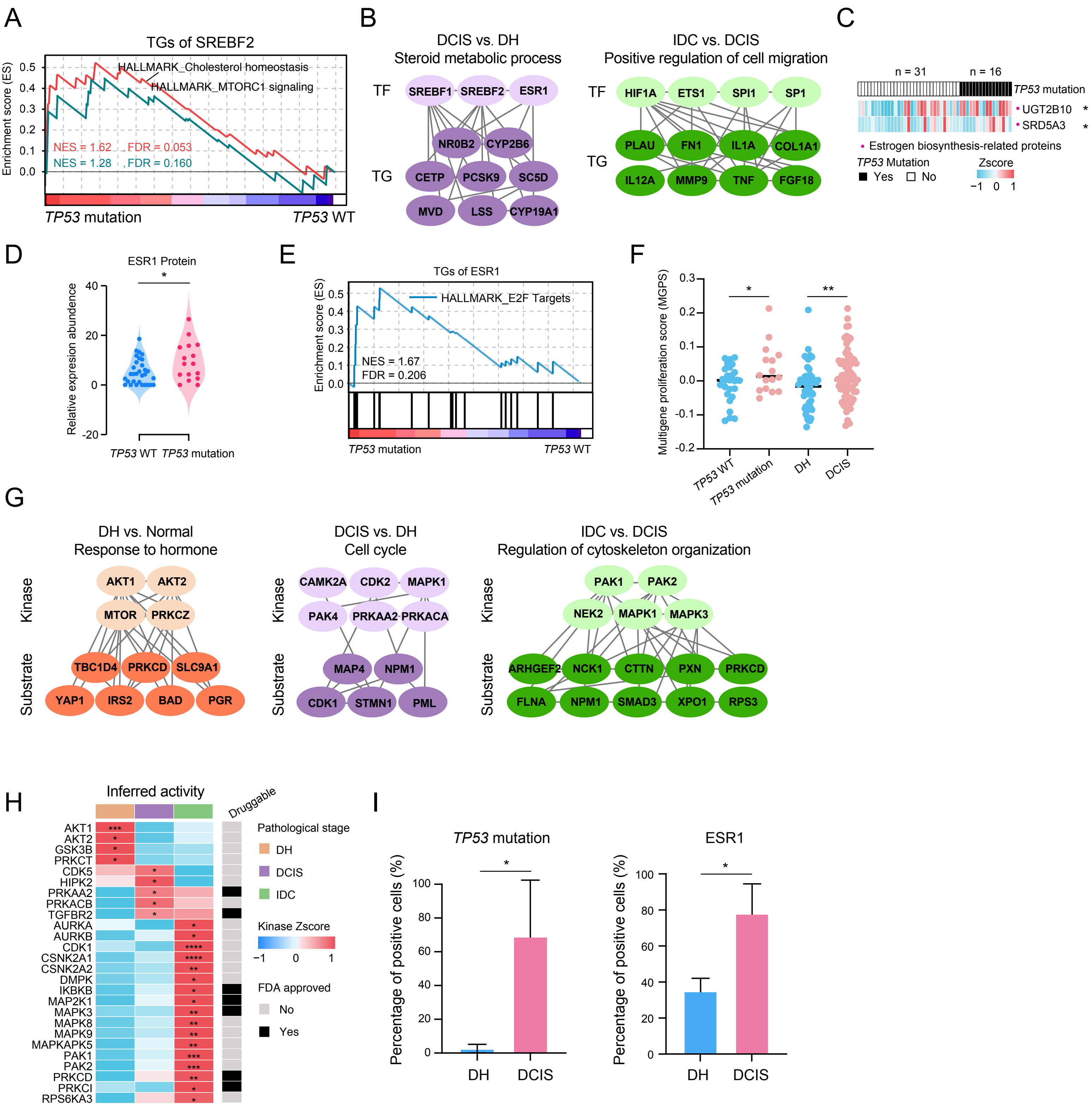

Figure S3. *TP53* Mutation-Associated ESR1 Overexpression was Involved in Tumorigenesis of BRDC, related to Figure 2

(A) GSEA showed that the TGs of SREBF2 were involved in the cholesterol homeostasis pathway in the *TP53* mutation group.

(B) The regulation networks of TFs and TGs in different stages of BRDC progression.

(C) Heatmap showing the protein expression levels of the estrogen synthesis-related proteins in the *TP53* wild-type group (n = 31) and the *TP53* mutation group (n = 16). Student's *t*-test, $*p < 0.05$.

(D) Violin diagram illustrating the protein expression levels of ESR1 in the *TP53* wild-type group (n = 31) and the *TP53* mutation group (n = 16). Student's *t*-test, $*p < 0.05$.

(E) GSEA showed that the TGs of ESR1 were involved in the proliferation-related pathways in the *TP53* mutation group.

(F) Boxplots showing the multigene proliferation scores (MGPS) in the *TP53* wild-type group (n = 31), the *TP53* mutation group (n = 16), DH (n = 54), and DCIS (n = 73). Student's *t* test, $*p < 0.05$, $**p < 0.01$.

(G) The regulation networks of kinases and substrates in different stages of BRDC progression.

(H) Evaluation of specific kinase activities in different stages of BRDC progression via KSEA.

(I) Boxplots showing the quantification of the IHC results. Student's *t*-test, $*p < 0.05$.

Supplementary Figure 4

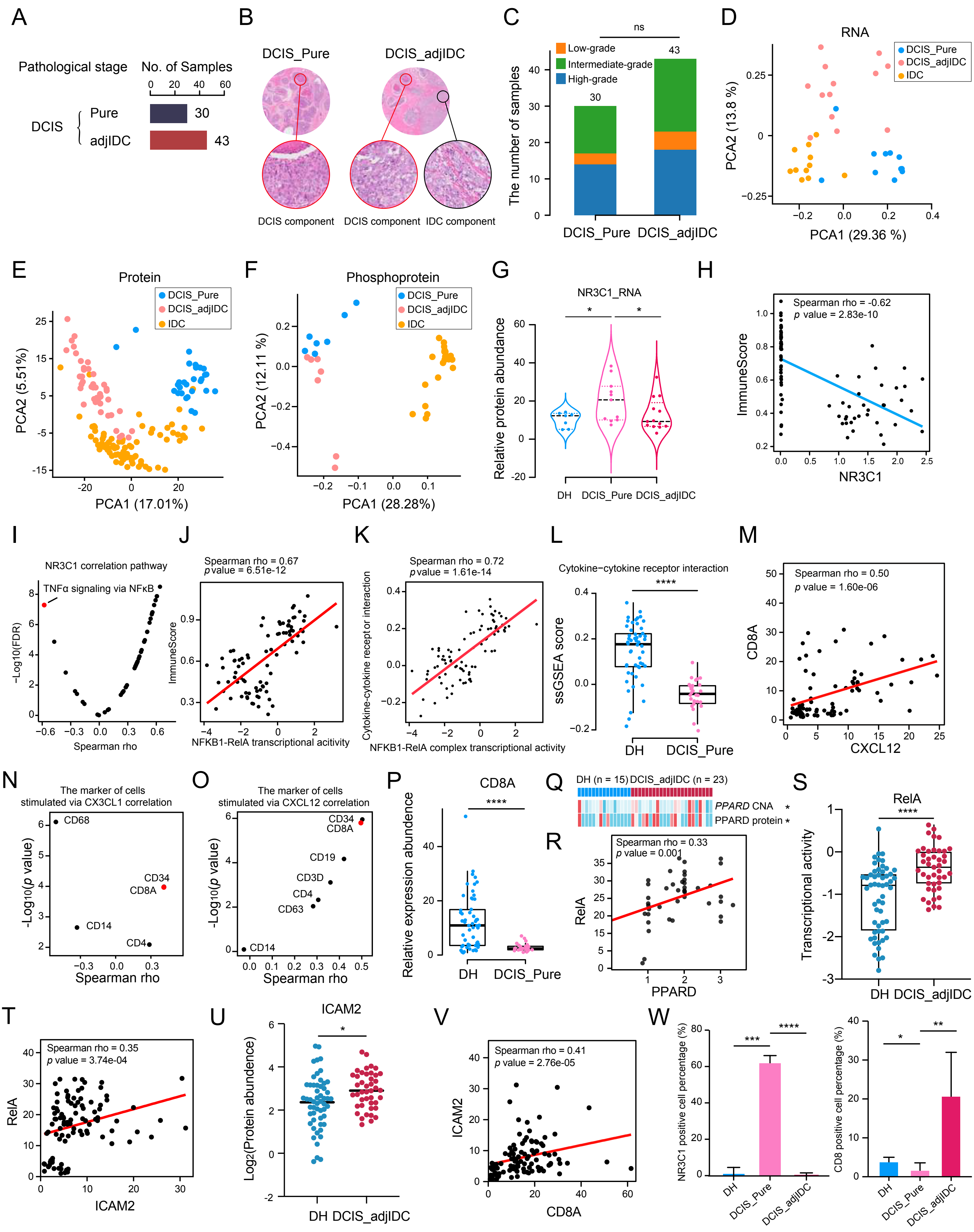

Figure S4. 6q21 Amplification-Associated NR3C1 Overexpression was Involved in Immune Escape of Tumor Cells in DCIS_Pure, related to Figure 3

(A) Two subtypes of DCIS in our cohort: DCIS_Pure (n = 30) and DCIS_adjIDC (n = 43).

(B) Representative H&E-stained slide of DCIS_Pure (no histologic evidence of invasion) and DCIS_adjIDC (DCIS regions of invasive cancers). The red circle indicates the sampling area.

(C) The histogram showing the proportion of pathological grades of DCIS in DCIS_Pure and DCIS_adjIDC cohorts.

(D-F) Principal component analysis (PCA) of transcriptomic (D), proteomic (E), and phosphoproteomic (F) data among DCIS_Pure, DCIS_adjIDC, and IDC.

(G) The transcription level of NR3C1 was upregulated in DCIS_Pure. Student's *t*-test, $^{*}p < 0.05$.

(H) Spearman-rank correlation of the protein expression levels of NR3C1 and immune score.

(I) Volcano plot showing the correlation between the NR3C1 expression level and the pathway scores by ssGSEA. The one highlighted in red represents the pathway of TNF-α signaling via NF-κB. The *p* value was calculated by Spearman's correlation test.

(J) Spearman-rank correlation of the transcriptional activity of the NF-κB1-RelA complex and immune score.

(K) Spearman-rank correlation of the transcriptional activity of the NF-κB1-RelA complex and the pathway score of cytokine-cytokine receptor interaction.

(L) Comparison of the pathway scores of cytokine-cytokine receptor interaction between DH (n = 54) and DCIS_Pure (n = 30). Student's *t*-test, $^{****}p < 0.0001$.

(M) Spearman-rank correlation of the protein expression levels of CXCL12 and CD8A.

(N) Spearman-rank correlation of the protein expression levels of CX3CL1 and the markers of cells. The one highlighted red represents CD8A.

(O) Spearman-rank correlation of the protein expression levels of CXCL12 and the markers of cells. The one highlighted red represents CD8A.

(P) Comparison of the protein expression levels of CD8A between DH (n = 54) and DCIS_Pure (n = 30). Student's *t*-test, $^{****}p < 0.0001$.

(Q) Heatmap showing the *cis*-effect gene PPARD (Student's *t*-test, $p < 0.05$).

(R) Spearman-rank correlation of the protein expression levels of PPARD and RelA.

(S) Boxplot showing the transcriptional activities of RelA in DH (n = 54) and DCIS_adjIDC (n = 43). Student's *t*-test, $^{****}p < 0.0001$.

(T) Spearman-rank correlation of the protein expression levels of ICAM2 and RelA.

(U) The protein expression level of ICAM2 in DH (n = 54) and DCIS_adjIDC (n = 43). Student's *t*-test, $^{*}p < 0.05$.

(V) Spearman-rank correlation of the protein expression levels of ICAM2 and CD8A.

(W) Boxplots showing the quantification of the IHC results. Student's *t*-test, $^{*}p < 0.05$, $^{**}p < 0.01$, $^{***}p < 0.001$, $^{****}p < 0.0001$.

Supplementary Figure 5

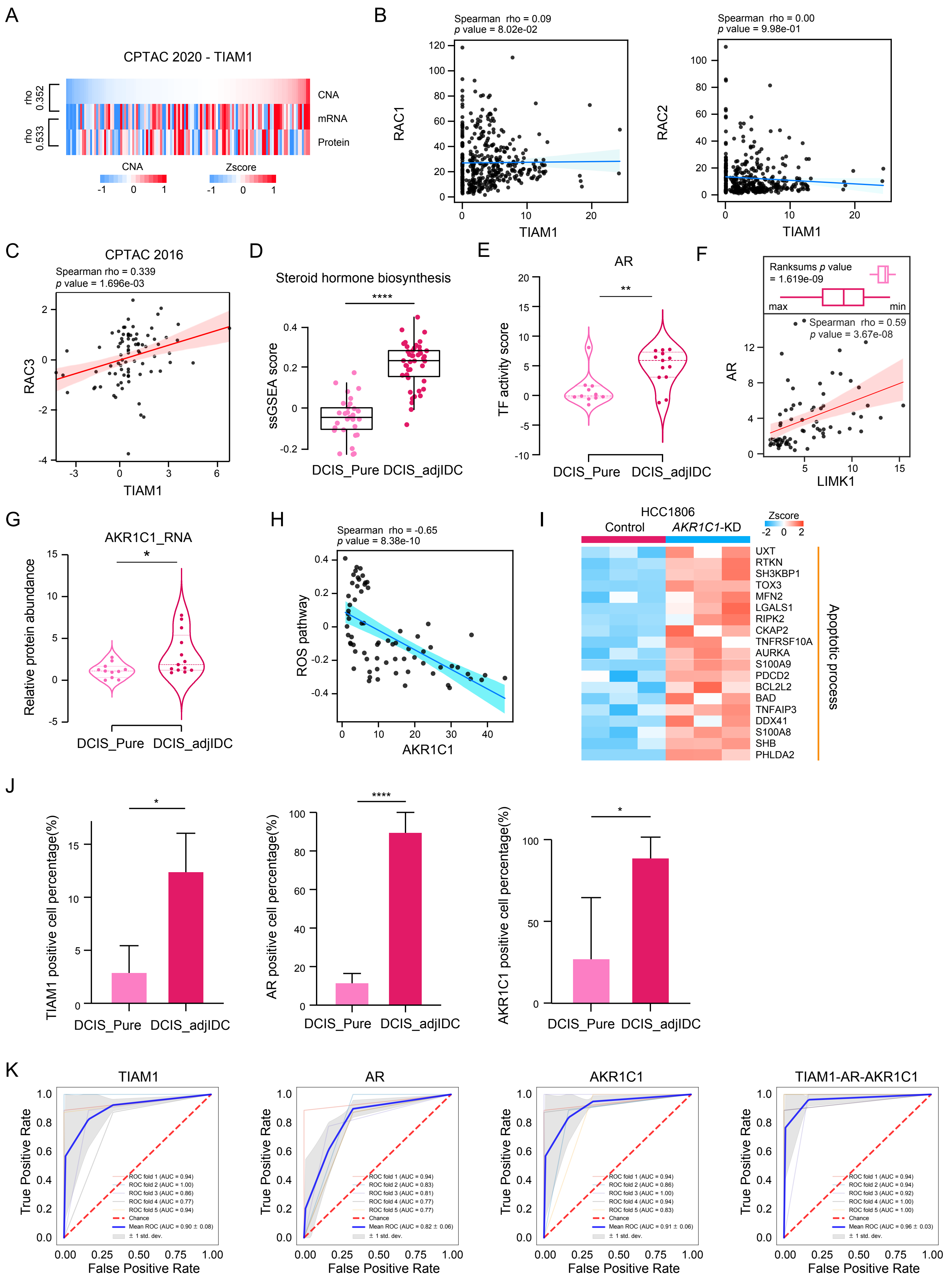

Figure S5. Proteogenomic Analysis Indicated the TIAM1-AR-AKR1C1 Axis Promoted Cell Invasion and Migration in DCIS_adjIDC, related to Figure 5

(A) The *cis*-effect gene TIAM1 in CPTAC 2020 cohort.

(B) Spearman-rank correlation of the protein expression levels of TIAM1 and RAC1 (left). Spearman-rank correlation of the protein expression levels of TIAM1 and RAC2 (right).

(C) Spearman-rank correlation of the protein expression levels of TIAM1 and RAC3 in CPTAC 2016 cohort.

(D) Comparison of the pathway scores of steroid hormone biosynthesis between DCIS_Pure (n = 30) and DCIS_adjIDC (n = 43). Student's *t*-test, ****$p < 0.0001$.

(E) Violin diagram illustrating the transcription activity of AR in DCIS_Pure (n = 30) and DCIS_adjIDC (n = 43). Student's *t*-test, **$p < 0.01$.

(F) Boxplot showing the protein expression levels of LIMK1 between DCIS_Pure (n = 30) and DCIS_adjIDC (n = 43) (upper). Spearman-rank correlation of the protein expression levels of LIMK1 and AR (bottom).

(G) Violin diagram illustrating the transcription level of *AKR1C1* in DCIS_Pure (n = 30) and DCIS_adjIDC (n = 43). Student's *t*-test, *$p < 0.05$.

(H) Spearman-rank correlation of the protein expression levels of AKR1C1 and the ROS pathway scores by ssGSEA.

(I) Heatmap showing the expression levels of apoptosis-related proteins in *AKR1C1*-knockdown HCC1806 cells and wild-type cells (Student's *t*-test, $p < 0.05$).

(J) Boxplots showing the quantification of the IHC results. Student's *t*-test, *$p < 0.05$, ****$p < 0.0001$.

(K) The 5-fold cross-validation area under the receiver operating characteristic (AUC) of TIAM1, AR, AKR1C1, and the TIAM1-AR-AKR1C1 panel.

Supplementary Figure 6

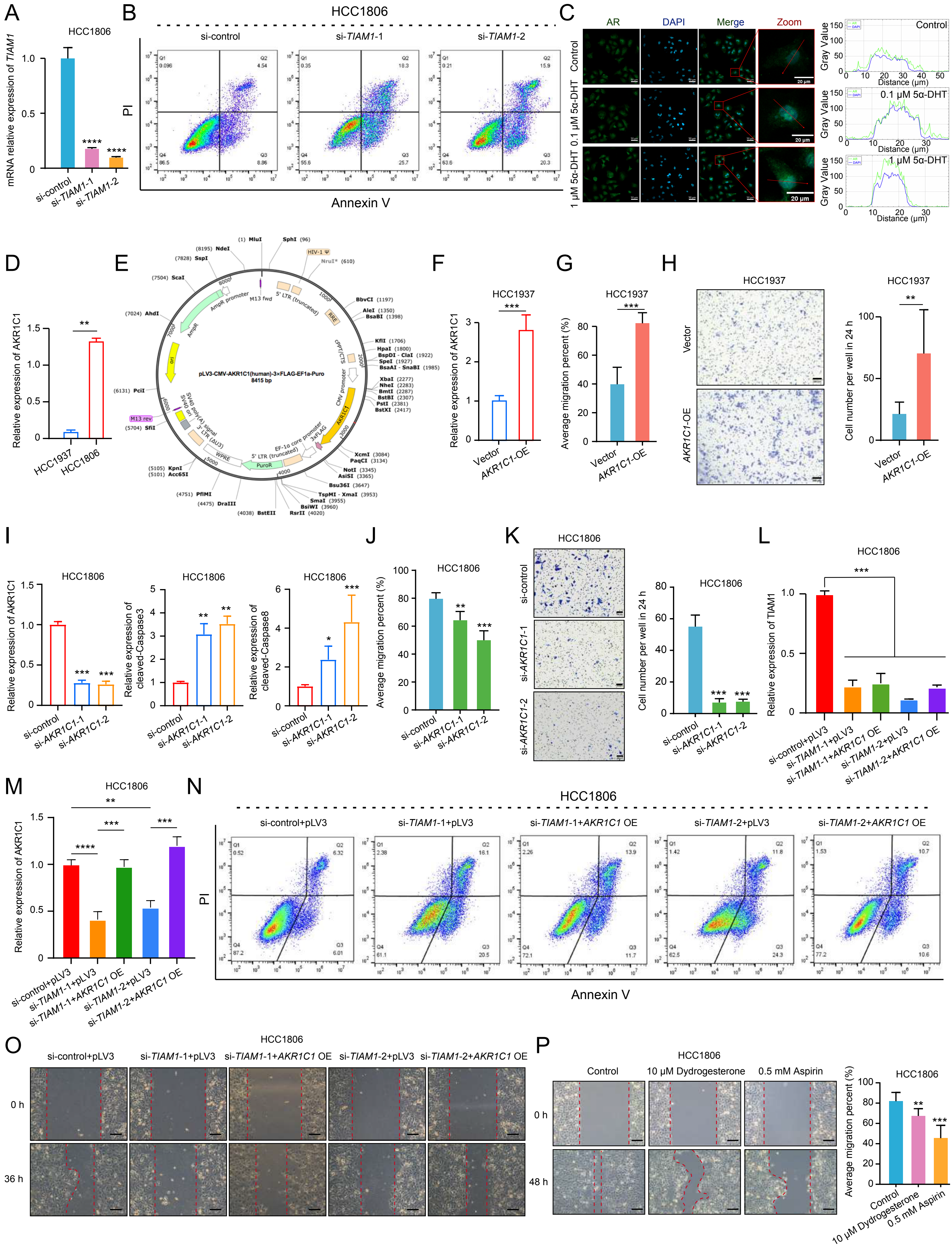

Figure S6. AKR1C1 is a Potential Targetable Protein in BC, related to Figure 6

(A) Real-time PCR analysis of *TIAM1* in *TIAM1*-knockdown HCC1806 cells. Student's *t*-test, ****$p < 0.0001$.

(B) Flow cytometry analysis with Annexin V-PI staining was performed to evaluate the percentage of apoptotic cells in *TIAM1*-knockdown HCC1806 cells.

(C) Representative image showing that 5α-DHT promoted AR nuclear translocation in HCC1806 cells (left). Plots of pixel intensity along the red line from top, middle, and bottom rows of images to the left of each plot (right). Scale bars = 20 μm.

(D) The relative expression of AKR1C1 in HCC1937 and HCC1806 cells was detected by western blotting. Student's *t*-test, **$p < 0.01$.

(E) A schematic with the essential plasmid data.

(F) The relative expression of AKR1C1 in *AKR1C1*-overexpression HCC1937 cells was detected by western blotting. Student's *t*-test, ***$p < 0.001$.

(G) The average migration percent of *AKR1C1*-overexpression HCC1937 cells was quantified. Student's *t*-test, ***$p < 0.001$.

(H) Representative sights of control or *AKR1C1*-overexpression HCC1937 cells in a transwell assay (left). The average number of cells in each sight was calculated (right). Scale bars = 100 μm.

(I) The quantitative data of the protein expression of AKR1C1 and the apoptosis-related proteins in HCC1806 cells expressing the control siRNA or *AKR1C1* siRNA vector. Student's *t*-test, *$p < 0.05$, **$p < 0.01$, ***$p < 0.001$.

(J) The average migration percent of *AKR1C1*-knockdown HCC1806 cells was quantified. Student's *t*-test, **$p < 0.01$, ***$p < 0.001$.

(K) Representative sights under the microscope of migrated HCC1806 cells on the lower side of the well and that of cells with transient knockdown of AKR1C1 via siRNA (left). The average number of cells in each sight was calculated (right). Scale bars = 100 μm.

(L) Control vector or *AKR1C1* overexpression vector was expressed in *TIAM1*-knockdown HCC1806 cells, and the quantitative data of the protein expression of TIAM1. Student's *t*-test, ***$p < 0.001$.

(M) Control vector or *AKR1C1* overexpression vector was expressed in *TIAM1*-knockdown HCC1806 cells, and the quantitative data of the protein expression of AKR1C1. Student's *t*-test, ** $p < 0.01$, ***$p < 0.001$, ****$p < 0.0001$.

(N) Control vector or *AKR1C1* overexpression vector was expressed in *TIAM1*-knockdown HCC1806 cells, and flow cytometry analysis with Annexin V-PI staining was performed to evaluate the percentage of apoptotic cells.

(O) Wound healing assays were performed on *TIAM1*-knockdown HCC1806 cells expressing control vector or *AKR1C1* overexpression vector. Scale bars = 100 μm.

(P) Wound healing assays were performed on HCC1806 cells or that treatment with dydrogesterone (10 μM) or aspirin (0.5 mM) (left). Scale bars = 100 μm. The average migration percent was calculated (right). Student's *t*-test, **$p < 0.01$, ***$p < 0.001$.

Supplementary Figure 7

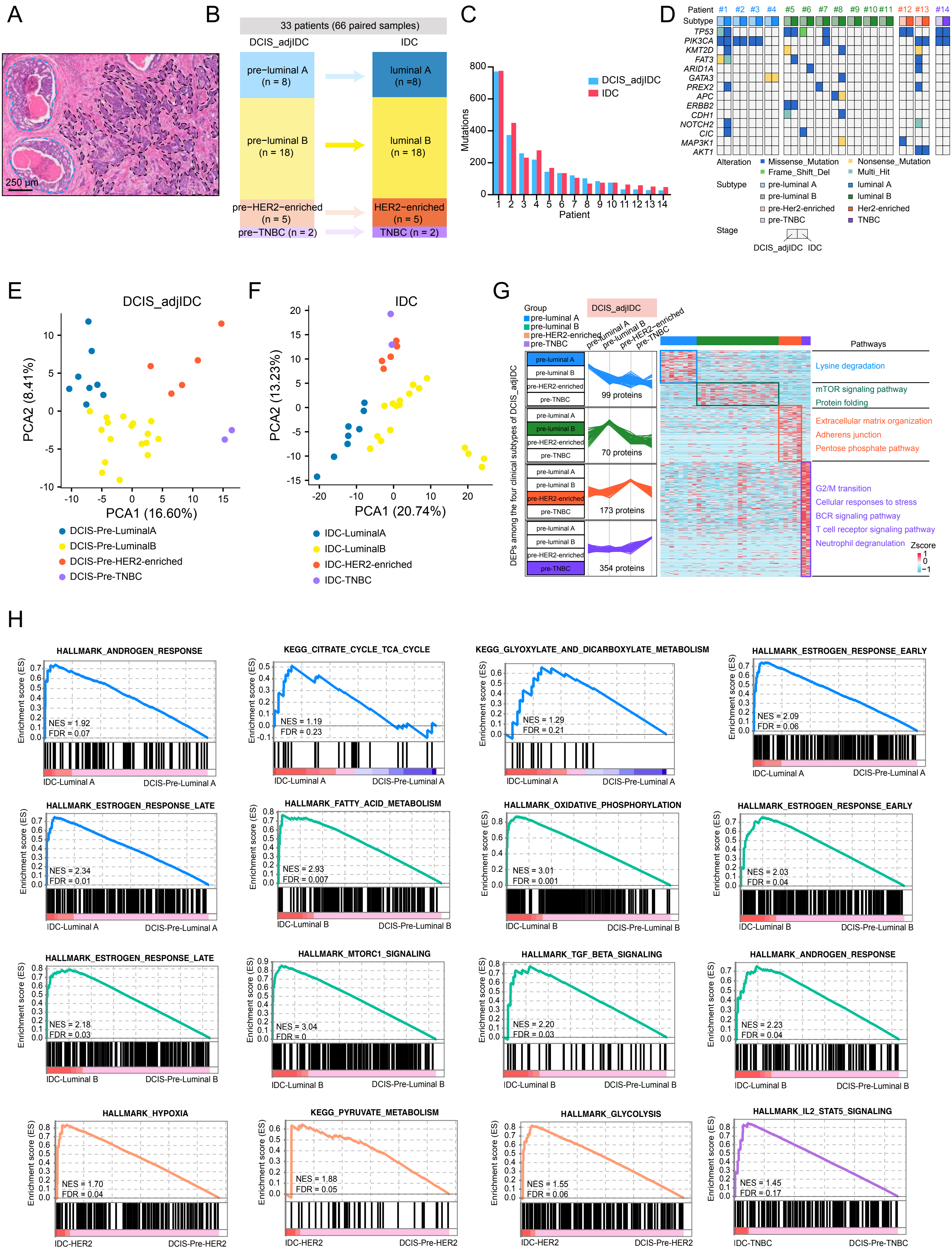

Figure S7. The Progression routes of transition from DCIS to four clinical subtypes of IDC, related to Figure 7

(A) Representative hematoxylin and eosin (H&E)-stained slide of IDC patient which synchronously diagnosed with DCIS and IDC components. The blue dotted circle indicates the sampling areas of IDC samples circled by the pathologist, and the black dotted circle indicates the sampling areas of DCIS_adjIDC samples circled by the pathologist. Scale bars = 250 μm.

(B) Paired DCIS and IDC samples.

(C) Bar plot of mutation numbers detected in DCIS_adjIDC and IDC of 14 DCIS-IDC patients.

(D) Comparison of mutations of the known breast cancer genes in 28 paired DCIS_adjIDC and IDC samples from 14 patients.

(E) PCA of proteomic data among IDC-luminal A, IDC-luminal B, IDC-HER2-enriched, and IDC-TNBC.

(F) PCA of proteomic data among DCIS-Pre-luminal A (n = 8), DCIS-Pre-luminal B (n = 18), DCIS-Pre-HER2-enriched (n = 5), and DCIS-Pre-TNBC (n = 2).

(G) The heatmap showing the proteome difference among the four clinical subtypes of IDC paired DCIS. The top panel shows the clinical annotation ER, PR and HER2 status in clinical data and its related protein expression in proteome data. The bottom panel shows the differently expressed proteins (DEPs) among four clinical subtypes of IDC. The left panel shows 99, 70, 173, and 354 proteins that were significantly overrepresented in pre-luminal A (n = 8), pre-luminal B (n = 18), pre-HER2-enriched (n = 5), and pre-TNBC (n = 2), respectively (Fold change > 2, Student's *t*-test, $p < 0.05$). The right panel shows the significantly enriched gene set in four clinical subtypes based on DEPs and the expression of the proteins involved in each gene set among four subtypes.

(H) GSEA was performed on four clinical subtypes of IDC and paired DCIS samples. The dot size represents the NES, and the dot color represents the significance. NES, normalized enrichment score.

Supplementary Figure 8

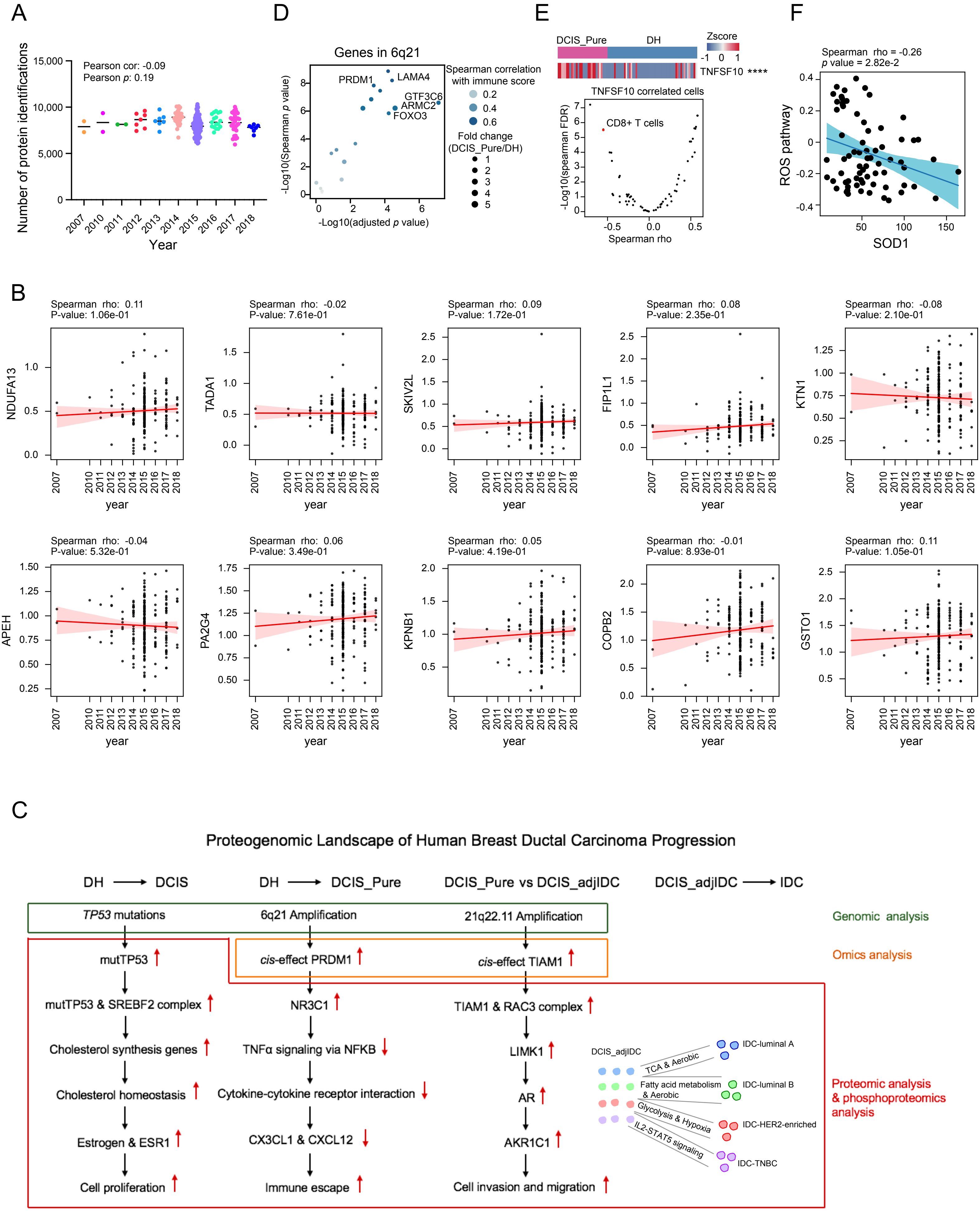

Figure S8 A brief summary of the signature in BRDC progression used omics analysis in this study.

(A) The protein identification number of the samples spanning from 2007 to 2018.

(B) The protein expression levels of housekeeping genes of the samples spanning from 2007 to 2018.

(C) A brief summary of the signature in BRDC progression used omics analysis.

(D) Comparison of the protein expression of the genes in 6q21 between DCIS_Pure (n = 9) and DH (n = 15), and their correlation with immune score.

(E) Comparison of the protein expression of the target gene of the FOXO3 between DCIS_Pure (n = 30) and DH (n = 54) (Student's *t*-test, ****p < 0.0001) (upper). The correlation of the protein expression of TNFSF10 and the immune score of cells by xCell (bottom).

(F) Spearman-rank correlation of the protein expression levels of SOD1 and the ROS pathway scores by ssGSEA.
